# Thioglycosides Act as Metabolic Inhibitors of Bacterial Glycan Biosynthesis

**DOI:** 10.1021/acsinfecdis.3c00324

**Published:** 2023-09-12

**Authors:** Isabella de la Luz Quintana, Ankita Paul, Aniqa Chowdhury, Karen D. Moulton, Suvarn S. Kulkarni, Danielle H. Dube

**Affiliations:** † Department of Chemistry & Biochemistry, Bowdoin College, 6600 College Station, Brunswick, Maine 04011, United States; ‡ Department of Chemistry, Indian Institute of Technology Bombay, Powai, Mumbai 400-076, India

**Keywords:** glycan, azide, metabolic labeling, bioorthogonal chemistry

## Abstract

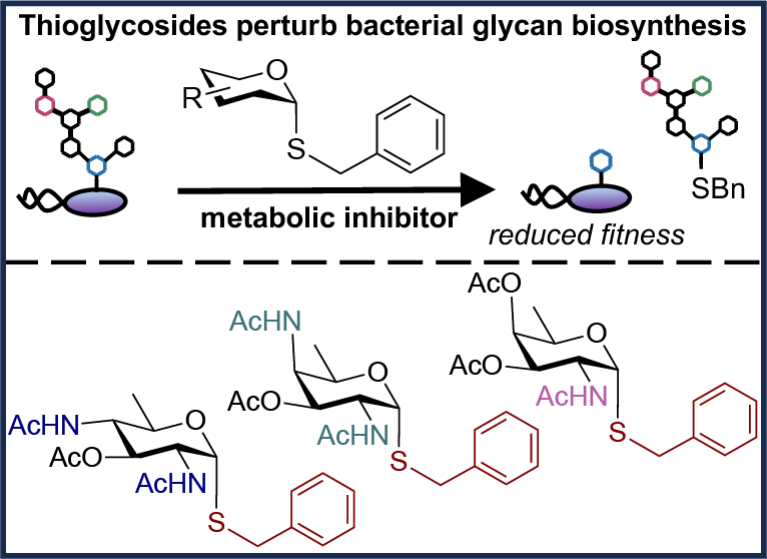

Glycans that coat the surface of bacteria are compelling antibiotic targets because they contain distinct monosaccharides that are linked to pathogenesis and are absent in human cells. Disrupting glycan biosynthesis presents a path to inhibiting the ability of a bacterium to infect the host. We previously demonstrated that O-glycosides act as metabolic inhibitors and disrupt bacterial glycan biosynthesis. Inspired by a recent study which showed that thioglycosides (S-glycosides) are 10 times more effective than O-glycosides at inhibiting glycan biosynthesis in mammalian cells, we crafted a panel of S-glycosides based on rare bacterial monosaccharides. The novel thioglycosides altered glycan biosynthesis and fitness in pathogenic bacteria but had no notable effect on glycosylation or growth in beneficial bacteria or mammalian cells. In contrast to findings in mammalian cells, S-glycosides and O-glycosides exhibited comparable potency in bacteria. However, S-glycosides exhibited enhanced selectivity relative to O-glycosides. These novel metabolic inhibitors will allow selective perturbation of the bacterial glycocalyx for functional studies and set the stage to expand our antibiotic arsenal.

Bacteria cover their cells with a carbohydrate-based coat of armor termed the glycocalyx. Proper construction of cell envelope glycans that comprise the glycocalyx, including peptidoglycan, lipopolysaccharide (LPS), capsular polysaccharide (CPS), and glycosylated proteins (Figure 1A), is critical for bacterial fitness and survival.^1^ Among their roles, glycans mediate host cell binding,^2^ evade immune cells,^3^ contribute to biofilm formation,^4,5^ aid with protein folding, and provide structural rigidity.^6^  As a testament to their functional and clinical importance, bacterial glycans are the target of blockbuster antibiotics including penicillin^7^ and vancomycin^8^ and antibiotics of last resort such as polymyxin.^9^ Due to the emergence and spread of resistance to existing antibiotics, there is an urgent need to identify novel molecules that perturb bacterial glycan biosynthesis.

**Figure 1:**
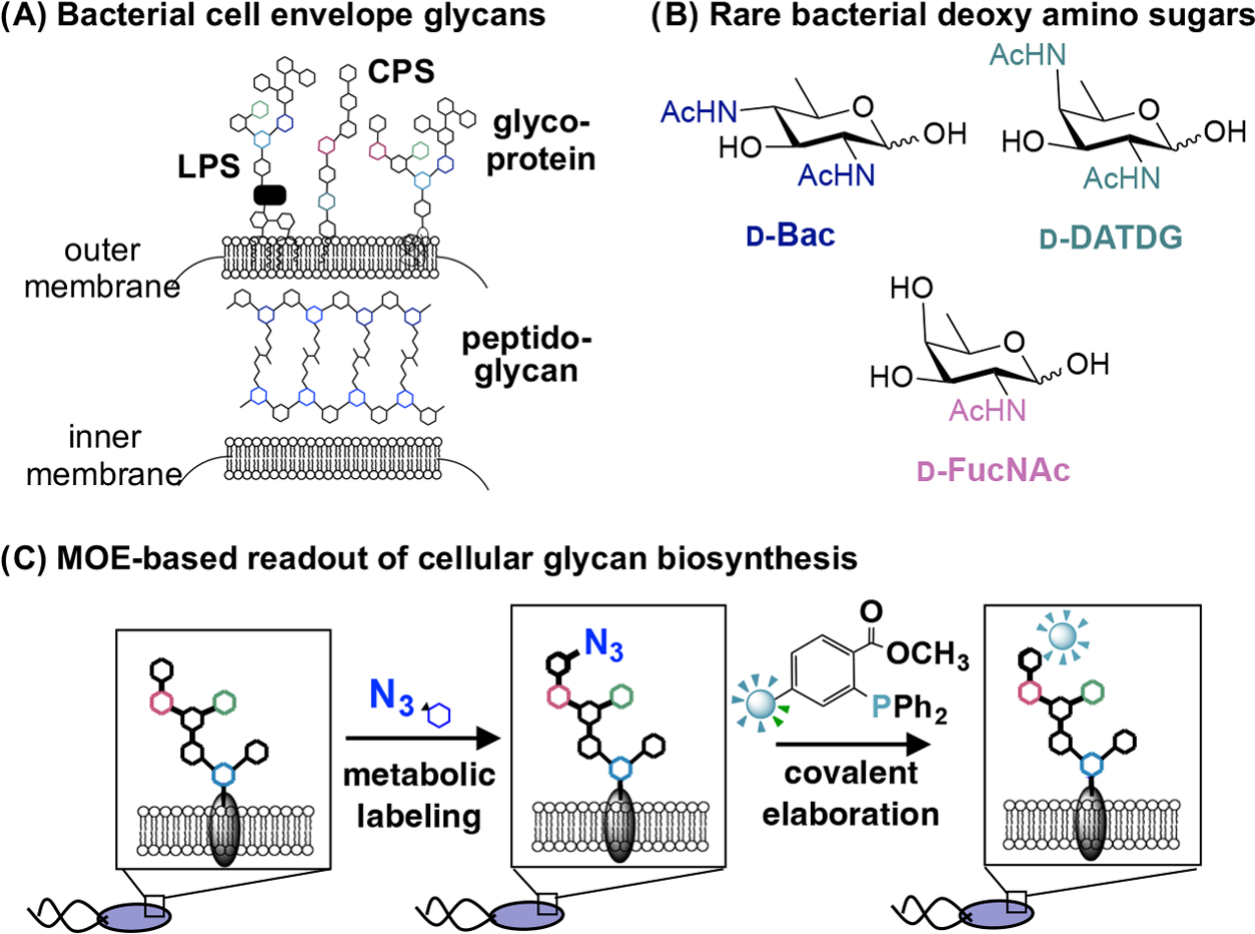
Sampling of exclusively bacterial (A) glycans, (B) monosaccharides synthesized by Gram-negative pathogens, and (C) an MOE-based method to report on bacterial glycan biosynthesis.

Bacterial glycans are compelling molecular targets due to their unique structures.^10,11^ While mammalian and bacterial cells are both coated with a glycocalyx, their structures are markedly different. Glycans on mammalian cells are composed of only 10 monosaccharides, whereas ≥700 monosaccharides constitute bacterial glycans.^12^ These monosaccharides include rare deoxy amino sugars that are expressed by a subset of medically significant bacterial pathogens. For example, di-*N*-acetyl D-bacillosamine (D-Bac),^13^ D-2,4-diacetamido-2,4,6-trideoxy galactose (D-DATDG), and *N*-acetyl D-fucosamine (D-FucNAc)^14,15^ are a sampling of exclusively bacterial sugars incorporated into higher-order glycans on the priority pathogens *Helicobacter pylori*,^16,17^*Campylobacter jejuni*,^18^ and *Pseudomonas aeruginosa*(19) (Figure 1B). Due to the presence of these monosaccharides on selected pathogens and their absence in mammalian cells, these structures are potential targets of selective interference.^20^

Genetic perturbation of glycan biosynthesis attenuates bacterial virulence. For example, *C. jejuni* protein glycosylation mutants led to reduced adherence and invasion of host cells.^21^ In *P. aeruginosa*, pilin glycosylation is a virulence factor that led to establishment and maintenance of infection in the lung.^22^ In *H. pylori*, insertional inactivation of the general protein glycosylation system led to less motility, diminished biofilm formation, and reduced adhesion to host cells relative to *H. pylori* with intact protein glycosylation.^23^ The essential role of bacterial glycans in pathogenesis underscores the need for small-molecule perturbation strategies.

There have been some recent successes with the development of small-molecule inhibitors of bacterial glycosylation enzymes. In 2014, Logan and co-workers performed a high-throughput screen to identify inhibitors of pseudaminic acid biosynthesis, a monosaccharide critical for flagellin glycosylation in *C. jejuni* and *H. pylori*.^24^ In 2017, De Schutter and colleagues used a fragment-based and high-throughput screening strategy to identify a potent small-molecule inhibitor of PglD, a UDP-amino-sugar acetyltransferase involved in D-Bac biosynthesis.^25^ In 2017, Xu and colleagues screened nonsubstrate-like molecules to identify a covalent inhibitor of LgtC, an α-1,4-galactosyltransferase involved in bacterial lipooligosaccharide biosynthesis.^26^ These inhibitors of glycosylation enzymes are potent at low concentrations but must overcome the obstacle of passing through the bacterial cell envelope to gain access to their enzyme targets. Moreover, in vitro screening requires knowledge of and access to glycan processing enzymes and their substrates, as well as robust assays; challenges accessing this information and these materials can stymie the process.

Cell-based assays offer an attractive alternative to screening glycosylation enzymes *in vitro*, as they embed cell entry as a criterion to identify “hits” and circumvent the need for full pathway characterization to screen inhibitors. In 2018, Zhang et al. used a cell-based screen to identify LPS biosynthesis and transport inhibitors, ultimately revealing an LPS biogenesis inhibitor.^27^ In Williams et al., we reported a metabolic oligosaccharide engineering (MOE)^11,28^ cell-based screen to identify inhibitors of bacterial glycan biosynthesis (Figure 1C).^29^ In this approach, we employed an azide-containing monosaccharide analogue that is processed by permissive glycan biosynthesis pathways to serve as a reporter for glycan biosynthesis in bacteria. Then, azide-selective bioorthogonal chemistries, including Staudinger ligation with phosphine probes and click chemistry with cyclooctyne probes,^30^ were used to detect intact versus disrupted glycan biosynthesis in cells treated with putative glycosylation inhibitors. This screening method, coupled to the synthesis of substrate analogues based on rare bacterial monosaccharide scaffolds, led to the discovery of O-glycoside metabolic inhibitors that disrupt bacterial glycan biosynthesis and recapitulate fitness defects observed by genetic disruption.^29^

The previously reported metabolic inhibitors **1**–**3** are O-glycosides that act as decoy substrates (Figure 2B).^29^ Briefly, substrate decoys compete with endogenous acceptor substrates for glycosyltransferase-mediated addition of monosaccharides.^31^ Treatment of cells with a substrate decoy leads to the buildup of glycans on substrate decoys rather than on endogenous substrates, causing concomitant truncation of newly synthesized glycans on the bacterial cell surface (Figure 2A). O-Glycoside-based substrate decoys have been employed to truncate cell surface glycans in a range of systems, from mammalian to bacterial cells^29^ yet are only partially effective even at millimolar concentrations.^32−35^ Recently, Matta, Neelamegham, and co-workers reported that thioglycosides (S-glycosides) are 10x more effective than O-glycosides at inhibiting glycan biosynthesis in mammalian cells.^32^ This enhanced inhibition is due in part to the resistance of S-glycosides to hydrolysis by glycosidases within mammalian cells.^32^ Motivated by this successful precedent, we sought to develop S-glycosides based on rare bacterial monosaccharide substrates and assess their efficacy as metabolic inhibitors of bacterial glycan biosynthesis.

**Figure 2:**
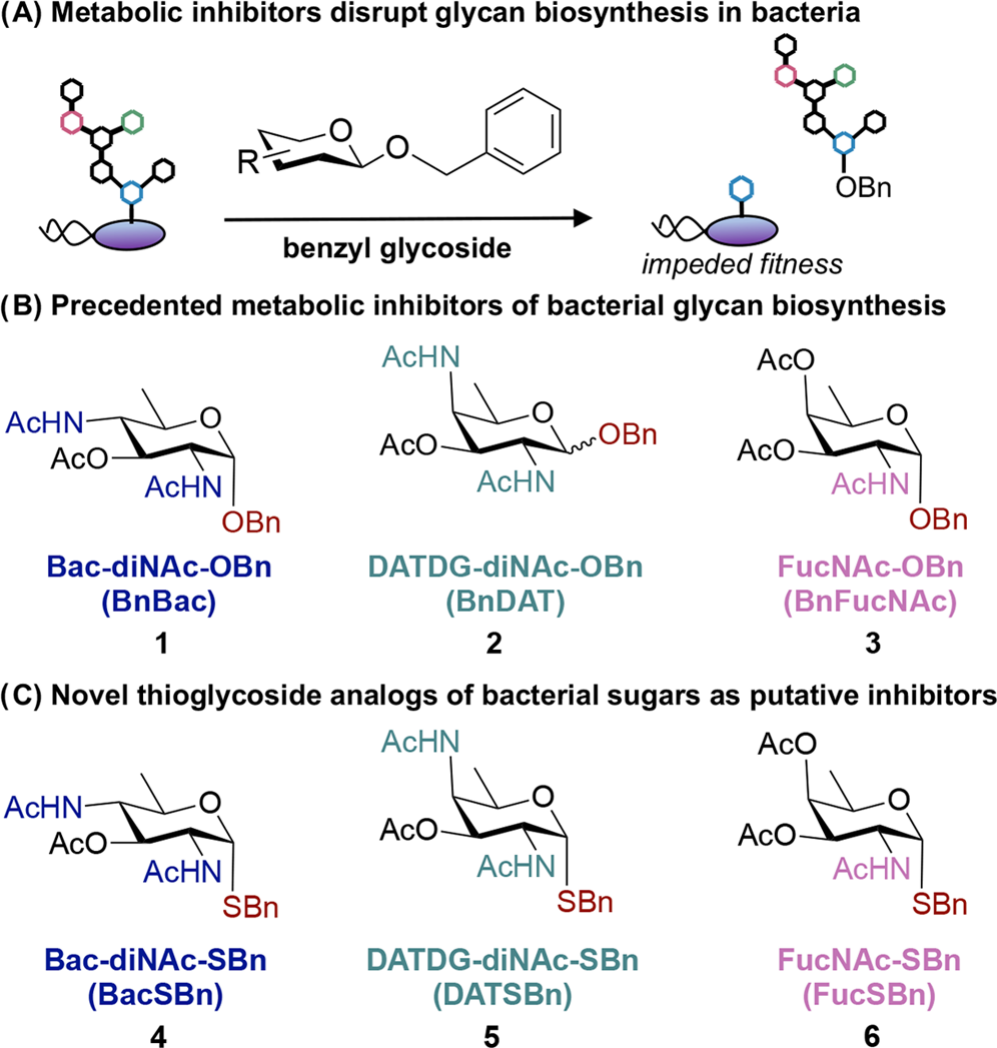
Metabolic inhibitors disrupt glycan biosynthesis. (A) Schematic of benzyl glycosides acting as metabolic inhibitors to divert glycan biosynthesis onto decoy substrates, ultimately leading to truncated glycans on bacteria. (B) Precedented O-glycoside inhibitors from previous work. (C) Novel S-glycoside analogues of bacterial sugar scaffolds used as putative metabolic inhibitors in this study.

Herein, we report the synthesis of novel S-glycoside analogues of the rare deoxy amino bacterial sugars D-Bac, D-DATDG, and D-FucNAc (Figure 2C; compounds **4**–**6**) and their evaluation as metabolic glycan inhibitors in pathogenic and symbiotic bacteria, as well as human host cells. As described below, the novel thioglycosides metabolically inhibit bacterial glycoprotein biosynthesis in *H. pylori* and precipitate a range of functional defects. By contrast, these analogues led to no detectable changes in glycan biosynthesis in the symbiotic intestinal bacteria *Bacteroides fragilis* and a human gastric adenocarcinoma cell line, both of which lack these rare monosaccharide scaffolds. In contrast to differences in potency in mammalian cells, S-glycosides and O-glycosides exhibited comparable potency in bacteria. However, S-glycosides exhibited enhanced selectivity relative to O-glycosides. These novel metabolic inhibitors set the stage for further probing and characterizing the functional consequence of perturbing glycans with selectivity. This work opens the door to unraveling structure–function relationships of bacterial glycans in the context of complex microbial communities, refining monosaccharide-based interference agents, and developing new glycosylation-based strategies to eradicate pathogenic infections.

## Results and Discussion

### Design and Synthesis of Thioglycosides

Inspired by the recent demonstration that thioglycosides are efficient substrate decoys that inhibit glycan biosynthesis in mammalian cells, we hypothesized that S-glycosides based on rare bacterial monosaccharide substrates would be effective metabolic inhibitors of bacterial glycan biosynthesis. We focused on derivatives of the rare bacterial monosaccharides D-Bac, D-DATDG, and D-FucNAc due to expedient syntheses of these scaffolds,^36,37^ their known utilization by select bacterial pathogens,^17^ and successes with O-glycosides based on these monosaccharides.^29^ As a first design element based on successful precedents with O-benzyl-glycosides to inhibit bacterial glycan biosynthesis (Figure 2B, **1**–**3**),^17,28^ we reasoned that S-benzyl-glycosides would likely be recognized by the requisite glycosyltransferases as decoy substrates. As a second design element, we chose to employ peracetylated analogues, as we and others have demonstrated that transient masking of hydrophilic hydroxyl groups on monosaccharide analogues with hydrophobic acetyl groups facilitates uptake and metabolic labeling in some bacteria.^17,28,38,39^ These design features are parallel to those adopted by Wang et al., who crafted the *N*-acetylglucosamine analogue-peracetylated *N*-acetylglucosamine-S-benzyl glycoside for their studies.^32^ Thus, we designed S-glycoside analogues BacSBn, DATSBn, and FucNSBn (Figure 2C, **4**–**6**) that embody these two design criteria.

**Scheme 1:**
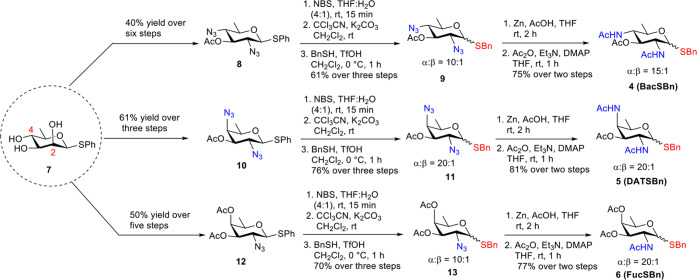
Synthesis of Thiobenzyl Analogues BacSBn **4**, DATSBn **5**, and FucSBn **6**

We synthesized the desired analogues **4**–**6** by adaptation of our previous approaches. In particular, the Kulkarni lab has established an efficient protocol for regioselective displacement of pyranosidic C-2, C-4 bistriflates with desired nucleophiles (azides or nitrites) giving access to a panel of functionalized rare sugar analogues including thioglycosides of bacillosamine, DAT, and fucosamine.^36,37^ Accordingly, the C-2 triflate is preferentially displaced using bulky TBAN_3_ at lower temperature (−30 °C) owing to stereo- and electronic effects, which leaves the C-4 triflate to be displaced by NO_2_^–^ to give an axially oriented C-4 hydroxyl. Here, we have exploited the protocol to install the thiobenzyl (SBn) handle on the known thioglycosides **8**, **10**, and **12**.^29,36^Scheme 1 outlines the combined synthetic route for the three thiobenzyl analogues, BacSBn **4**, DATSBn **5**, and FucSBn **6**.

To begin with the synthesis of DATSBn, we first tried a direct coupling of DAT-thioglycoside **10** with commercially available benzylmercaptan using NIS and TfOH promotion conditions. However, the SBn-coupled compound was not obtained, and the starting thioglycoside **10** was recovered as such. A similar observation was noted while proceeding via glycosyl bromide. Therefore, the thioglycoside was converted to a hemiacetal using NBS, THF/H_2_O (4:1) conditions and subsequently to a trichloroimidate using CCl_3_CN, K_2_CO_3_ in CH_2_Cl_2_. The obtained imidate donor on glycosylation with PhCH_2_SH in the presence of a TfOH promoter at 0 °C delivered the DATSBn-coupled compound **11** (α:β = 10:1) in 76% yield over three steps. The desired SBn-coupled product was confirmed by the presence of an AB quartet at δ 3.72 ppm in the ^1^H NMR spectrum and an inverted peak at δ 34.1 ppm in the ^13^C DEPT-135 NMR for CH_2_ of the thiobenzyl group. Finally, the C-2, C-4 diazide was converted to NHAc by sequential conversion to amines using Zn, AcOH, followed by peracetylation with Ac_2_O to give target DATSBn analogue **5** in 81% yield over two steps. Similar reaction conditions for glycosylation of glycosyl imidate donors from bacillosamine thioglycoside **8** and fucosamine thioglycoside **12** furnished thiobenzyl analogues of the respective sugars **9** (61% yield, α:β = 20:1) and **13** (70% yield, α:β = 10:1). Zinc mediated reduction and peracetylation of the azide to acetamido furnished the protected desired SBn analogues, BacSBn **4** and FucSBn **6**, in 75 and 77% yield, respectively, predominantly as α-anomers with a trace amount of the corresponding β-anomers.

### Thioglycosides Inhibit Glycoprotein Biosynthesis in *H. pylori*

**Figure 3:**
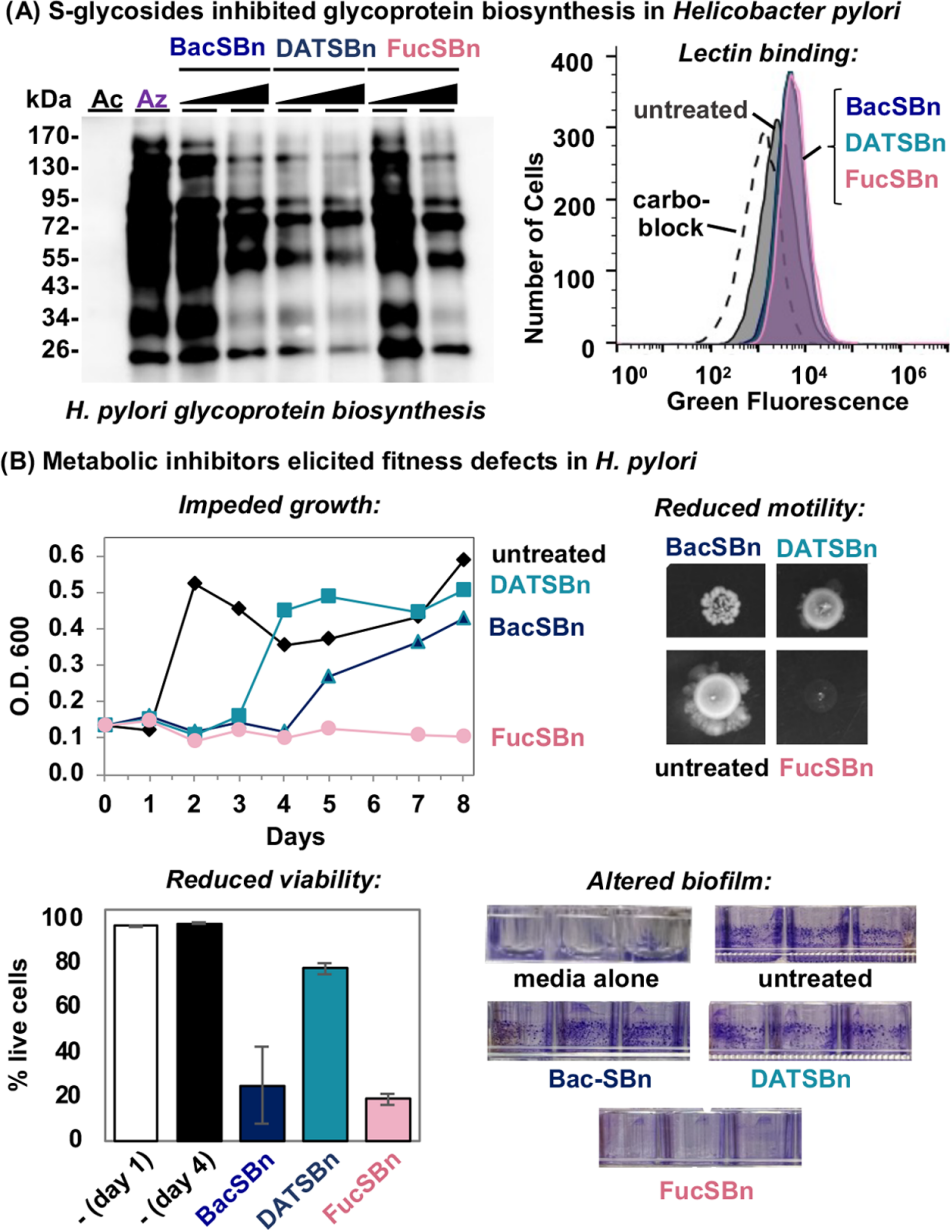
Novel S-glycosides inhibit glycan biosynthesis and decrease fitness in *Helicobacter pylori*. (A) Western blot analysis (left) reveals diminished glycoprotein biosynthesis in *H. pylori* upon treatment with BacSBn **4**, DATBn **5**, and FucSBn **6**. The short side of the triangle represents the 1 mM S-glycoside treatment, and the tall part represents the 2 mM S-glycoside treatment. Flow cytometry analysis (right) reveals increased binding of ConA lectin in *H. pylori* treated with S-glycosides, consistent with perturbed cell surface glycan architecture. By contrast, pretreatment of ConA with 400 mM mannose (carbo-block) prior to probing untreated *H. pylori* led to decreased binding. (B) Measurement of growth, viability, motility, and biofilm demonstrates a range of fitness defects in *H. pylori* treated with S-glycosides, with FucSBn leading to the most marked effects relative to untreated (−) controls. The data shown are representative of replicate experiments (*n* = 3).

To ascertain whether the newly developed S-glycoside analogues of rare bacterial D-sugars (Figure 2C, **4**–**6**) are effective metabolic glycan inhibitors, we evaluated their impact on glycoprotein biosynthesis in *H. pylori* using an established MOE-based assay.^29^ Briefly, this cell-based assay hinges upon the metabolic incorporation of the azide-containing sugar-peracetylated *N*-azidoacetylglucosamine (Ac_4_GlcNAz) into a suite of glycoproteins synthesized by *H. pylori*’s general glycosylation system.^38,40^ Here, we assessed the ability of each of the thioglycoside analogues to inhibit azide-labeled glycoprotein biosynthesis in *H. pylori*. Following our previously reported methods, metabolic labeling experiments were performed with Ac_4_GlcNAz as a positive control, with the azide-free sugar-peracetylated *N*-acetylglucosamine (Ac_4_GlcNAc) as a negative control or with Ac_4_GlcNAz in the presence of increasing concentrations (1–2 mM) of putative inhibitors **4**–**6** as experimental samples. After metabolic labeling for 4 days, proteins were harvested from lysed cells and reacted with a phosphine probe containing a FLAG peptide (Phos-FLAG) via Staudinger ligation to detect azide-labeled glycoproteins.^41,42^ Consistent with previous reports, Western blot analysis with anti-FLAG antibody revealed that *H. pylori* treated with Ac_4_GlcNAz synthesized the full complement of azide-labeled glycoproteins, whereas treatment with Ac_4_GlcNAc led to no detectable azide-dependent signal (Figure 3A).^29,38,40^ These samples represent the highest and lowest amounts of signal, respectively, that we could expect to see in any given sample. Experimental samples treated with BacSBn **4** and FucSBn **6** elicited a concentration-dependent diminishment in the azide-dependent glycoprotein profile, with subtle effects at 1 mM and substantial diminishment at 2 mM treatments (Figure 3A, left). By contrast, DATSBn **5** appeared to abrogate the azide-dependent signal at both concentrations tested (Figure 3A, left). Coomassie staining of electrophoresed samples confirmed that all samples contain protein (Figure S1A), indicating that abrogation in the azide-dependent signal was not due to large differences in protein loading. Altogether, these data indicate that the three novel S-glycosides inhibit glycoprotein biosynthesis in *H. pylori*.

We next turned to carbohydrate-binding lectins as a complementary means to assess the effect of thioglycosides on *H. pylori* surface glycans. In particular, we assessed the binding of the lectin *Concanavalin A* (ConA) to untreated *H. pylori* versus *H. pylori* treated with 2 mM thioglycosides **4**–**6**. Flow cytometry analysis revealed that ConA bound to untreated *H. pylori* at modest levels, and ConA binding was diminished by pretreatment of ConA with high concentrations of its monosaccharide ligand mannose (Figure 3A, right). Relative to untreated *H. pylori*, cells that were treated with BacSBn **4**, DATSBn **5**, and FucSBn **6** exhibited an increase in ConA binding (Figure 3A, right; Figure S1B), consistent with altered cell envelope glycan architecture. This result is in line with previous reports of small molecules or genetic disruption of glycoprotein biosynthesis causing increased lectin binding.^23,29^

Once we established that thiobenzyl glycosides disrupt glycan biosynthesis in *H. pylori*, we explored the effect of these compounds on fitness attributes. In particular, we scored fitness attributes that are critical for *H. pylori* to colonize and infect the host. Briefly, *H. pylori* was treated with S-glycosides at their lowest effective concentration (2 mM BacSBn **4** and FucSBn **6**, 1 mM DATSBn **5**) or left untreated; then relative strain fitness was scored. Monitoring growth by optical density at 600 nm (OD_600_) revealed that untreated cells reached a stationary phase after 2 days, while cells treated with DATSBn **5** and BacSBn **4** had lagging growth relative to wild type but ultimately reached the stationary phase (Figure 3B). By contrast, cells treated with FucSBn **6** maintained a low OD_600_ over the course of 8 days, suggesting that this compound significantly diminished *H. pylori* growth relative to untreated samples. Viability was measured by BacLight live–dead assay and indicated that all three S-glycosides reduced viability relative to untreated *H. pylori*, with BacSBn **4** and FucSBn **6** causing the most pronounced cell death (Figures 3B and S1C). Similarly, **4**–**6** significantly reduced motility on soft agar plates^43^ relative to untreated cells (Figures 3B and S1C). Finally, untreated *H. pylori* produced a prominent biofilm that was detected by crystal violet staining;^44^ of the treated samples, only treatment of FucSBn **6** led to a substantial perturbation of biofilm formation (Figures 3B and S1C). Holistically, these data indicate that FucSBn **6** causes the most dramatic fitness effects.

Taken together, the results of these studies indicate that the novel thioglycosides act as metabolic inhibitors that impair glycoprotein biosynthesis in *H. pylori* and precipitate a range of functional defects. These results support the importance of *H. pylori*’s general protein glycosylation system in a variety of fitness attributes and offer important probes for understanding how glycan structure impacts function. The slightly different magnitudes of effects observed with **4**, **5**, and **6** may be due to where in the pathway these molecules intercept, possibly leading to relatively more or less elaborated glycans on cells as a result. Structural studies of glycans from treated versus untreated cells will shed light on the nuanced ways in which these S-glycosides inhibit glycan structures, thus opening the door to incisive structure–function relationships.

### S-Glycosides Do Not Disrupt Commensal Bacteria or Human Cells

**Figure 4:**
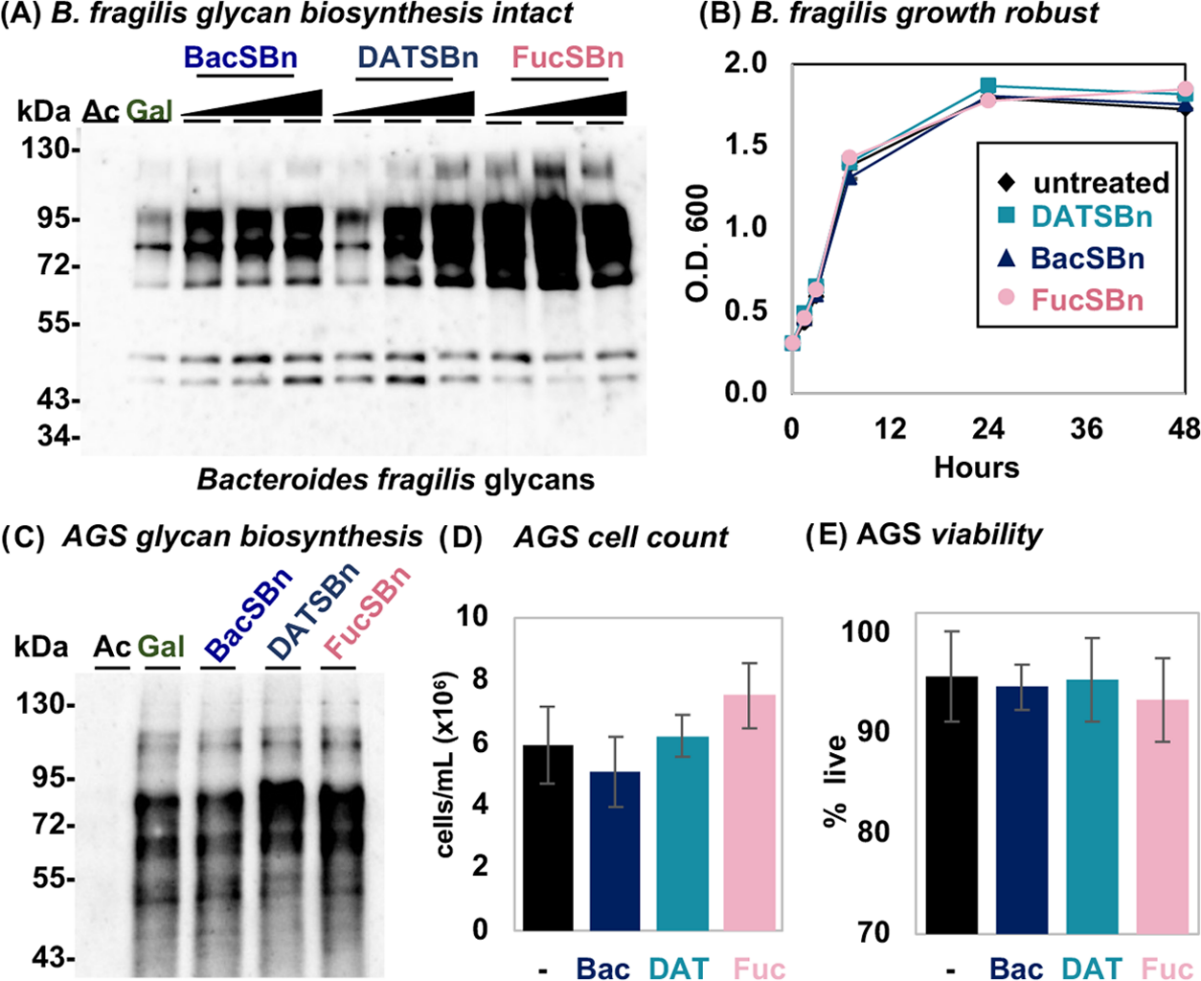
Glycan biosynthesis and growth remain intact in *B. fragilis* and mammalian cells following treatment with S-glycosides. (A) Western blot analysis shows intact biosynthesis of polysaccharide A in *B. fragilis* treated with 0.1–2 mM thioglycosides BacSBn **4**, DATSBn **5**, and FucSBn **6** that is comparable to levels in samples treated only with Ac_4_GalNAz. The increasing size of the shaded triangle corresponds to increasing concentrations of S-glycosides, with the short side of the triangle corresponding to 0.1 mM and the tallest part representing 2 mM. (B) *B. fragilis* exhibited robust growth across all treatments. (C) Western blot analysis of Ac_4_GalNAz-labeled O-linked glycoproteins in AGS samples indicates that treatment with 10 μM thioglycosides BacSBn **4**, DATSBn **5**, and FucSBn **6** has no apparent effect on glycoprotein biosynthesis. Density (D) and viability (E) of AGS cells are not significantly impacted by thioglycoside treatment relative to untreated controls (−). These data are representative of replicate experiments (*n* ≥ 2).

Once we established that S-glycosides **4**–**6** impede glycan biosynthesis in the pathogen *H. pylori*, we next explored the effect of these metabolic inhibitors on commensal gut bacteria. We focused on *Bacteroides fragilis*, as these bacteria are major constituents of the human gut that play critical roles in protection from pathogens and polysaccharide metabolism. Following Kasper and co-workers’ metabolic labeling method to incorporate azides into *B. fragilis* capsular polysaccharide A,^39^ bacteria were treated with peracetylated *N*-azidoacetylgalactosamine (Ac_4_GalNAz) and the effect of S-glycosides **4**–**6** on subsequent CPS biosynthesis was monitored. Consistent with literature reports,^39^ surface glycans were robustly azide-labeled upon supplementation of *B. fragilis* with Ac_4_GalNAz (Figure 4A). Treatment of *B. fragilis* with S-glycosides had no apparent diminishment of capsular polysaccharide A biosynthesis, as evidenced by the sustained robust azide-dependent signal observed in all treatments (Figures 4A and S2A). If anything, the treatment of *B. fragilis* with FucSBn **6** led to a subtle increase in CPS biosynthesis. *B. fragilis* supplemented with S-glycosides **4**–**6** grew robustly relative to an untreated control (Figure 4B). These data indicate that these S-glycosides have a negligible effect on *B. fragilis* glycan biosynthesis and growth. In contrast, our previous work found that the O-glycoside BnFucNAc **3** interfered with CPS biosynthesis in *B. fragilis*.^29^ These results suggest that S-glycosides based on rare bacterial monosaccharides exhibited an enhanced selectivity relative to that of O-glycosides, thus setting the stage for glycan-based interference of pathogenic bacteria in a selective manner.

Curious about the effect of S-glycosides based on rare bacterial sugars on host cells, we set out to evaluate the effect of these analogues in the well-studied human gastric adenocarcinoma (AGS) cell line, a cell line that is an established model for epithelial cells that line the gastrointestinal tract.^45^ Treatment of AGS cells with the positive control sugar Ac_4_GalNAz led to an array of azide-labeled glycans in lysates and on cells (Figures 4C and S2B), consistent with incorporation of this substrate into cell surface mucin-type O-linked glycoproteins.^46^ Similar to treatment with the positive control sugar alone, treatment of AGS cells with Ac_4_GalNAz and S-glycosides **4**–**6** led to a robust azide-labeled glycan fingerprint (Figure 4C). These results suggest that the S-glycosides tested in this study have a minimal effect on the biosynthesis of the O-linked glycoprotein in AGS cells. Scoring AGS fitness of untreated cells versus cells treated with S-glycosides **4**–**6** indicated robust cell density and viability across all samples (Figure 4D,E). These findings suggest relative selectivity for thioglycoside-based metabolic decoys based on rare bacterial sugars, which is consistent with expectations based on monosaccharide utilization across different cell types. To the best of our knowledge, this is the first evidence that metabolic decoys based on bacterial glycans leave host glycans intact.

## Discussion

Given the importance of glycan biosynthesis in bacterial fitness, survival, and pathogenesis, expanding the repertoire of tools to disrupt these structures is critical. In particular, small-molecule inhibitors of glycan biosynthesis offer a means to query structure–function relationships and indicate potential avenues for novel glycan-based interference strategies. Due to the profound structural complexity and diversity of bacterial glycans, coupled to a frequent lack of in-depth structural information about glycan biosynthesis pathways and enzymes, we turned to a substrate-based approach to design novel inhibitors. We built upon successful precedents from Esko,^33−35^ Kim,^47^ Matta, Neelamegham,^32^ and others who used substrate decoys to divert glycan biosynthesis in mammalian cells, as well as our own work with peracetylated benzyl glycoside analogues of rare bacterial monosaccharides to metabolically inhibit bacterial glycan biosynthesis.^29^ Here, inspired by the 10-fold enhanced efficacy of thiobenzyl glycosides compared to benzyl glycosides in mammalian cells reported by Wang et al.,^32^ we produced a series of thiobenzyl glycoside analogues of rare bacterial monosaccharides to augment the existing toolkit.

Our data indicate that the novel S-glycosides act as metabolic glycan inhibitors in *H. pylori*, yet they have no appreciable effect on glycan biosynthesis in *B. fragilis* or human cells. In particular, FucSBn **6** impeded glycoprotein biosynthesis and precipitated defects in growth, motility, and biofilm formation assays in *H. pylori* (Figure 3) yet left *B. fragilis* and mammalian cell glycan biosynthesis and growth intact (Figure 4). These observed species-selective effects are likely due to differences in utilization of the rare bacterial monosaccharides Bac, DAT, and FucNAc by these cell types. Indeed, *H. pylori* is known to metabolically incorporate azide-containing analogues of these three monosaccharides, while neither *B. fragilis* nor mammalian cells incorporate Bac, DAT, and FucNAc probes into their cellular glycans.^17^ Selectivity is a desirable feature for teasing out the role of bacterial glycans in more complex environments, including within gut microbial communities and animal infection models.^11^ Thus, the novel S-glycoside inhibitors set the stage for selective glycan perturbation studies.

Different effects were observed among the three thioglycosides. Specifically, the choice of monosaccharide scaffolds appeared to influence the extent and pattern of inhibition and fitness defects in *H. pylori* (Figure 3). FucSBn **6** was the only inhibitor that elicited profound fitness defects across all three fitness assays in *H. pylori* (Figure 3B). Metabolic inhibitors based on different monosaccharide scaffolds may elicit different effects by inhibiting glycan biosynthesis at discrete points during construction of the higher-order glycan by glycosyltransferases. The profound inhibition and fitness defects caused by FucSBn **6** suggest that FucNAc may be a lynchpin within the elaborated glycan structure without which cells fare poorly. Access to structural information about how each of the thioglycosides impacts glycan biosynthesis will be critical to fully understand the compound-specific differences observed.

The experiments conducted in this project do not shed light on the mechanism of the inhibition of these compounds. While it is established in mammalian systems that inhibitors of this design act as substrate decoys that divert glycan biosynthesis onto mock substrates, their precise mode of action has not been established in bacterial systems. Molecular-level evidence of the buildup of glycans on thioglycosides **4**–**6** within cells would confirm this proposed mechanism. Cummings, Neelamagham, Wang, and others have developed and applied mass spectrometry-based approaches to detect elaborated glycans on decoy scaffolds, facilitating the structural characterization of mammalian glycans.^32,48−50^ These approaches could be translated to the study of elaborated glycans on decoy scaffolds in bacteria. If this proposed mechanism is correct, novel thioglycosides could analogously serve as readouts of bacterial glycan biosynthesis, ultimately enabling glycomic analyses that yield structural information.

The original impetus for this study was to compare the efficacy of bacterial S-glycosides **4**–**6** to that of previously tested bacterial O-glycosides **1**–**3**. In contrast to the 10-fold difference in efficacy observed with S- versus O-glycosides based on GlcNAc in mammalian cells,^32^ S- and O-glycosides based on rare bacterial monosaccharides exhibited comparable potency in bacteria, with mM concentrations required for efficacy.^29^ Wang et al. posited that S-glycosides are more potent in mammalian cells due to their resistance to hydrolysis by mammalian hexosaminidases, leading to enhanced stability and subsequent inhibitory activity.^32^ Our data indicate Wang et al.’s proposed mechanism of O-glycoside hydrolysis and relative S-glycoside stability is not as relevant in bacterial cells as it is in mammalian systems. Hexosaminidase activity might be more abundant in mammalian cells than in bacterial cells. Alternatively, bacterial hexosaminidases may cleave thioglycosides relatively efficiently, consistent with recent findings by Withers and co-workers.^51^ Moreover, there may be other cellular factors at play in bacterial cells, such as efficiency of compound uptake across the bacterial cell envelope,^52^ that have a larger impact on overall efficacy of inhibitor than their relative resistance to hydrolysis. These findings raise questions about levels of glycosyl hydrolase activity in bacteria and their mechanisms of action,^53^ as well as mechanisms of sugar uptake across the cell envelope. Chemical tools that address these remaining unknowns will further refine our understanding of the parameters dictating bacterial glycan biosynthesis, stability, and metabolism.

Given the absence of Bac, DAT, and FucNAc from human cells and their variable expression across bacteria, these structures have the potential to form the basis of new glycosylation-based strategies to eradicate pathogenic infections. Selective perturbation of bacterial glycans offers a means to hypersensitize bacteria to existing antibiotics^54,55^ and tailor the bacterial glycocalyx for the modulation of the host immune response.^11,56,57^ One limitation of this suite of compounds is the millimolar concentration required for inhibition, which puts these agents outside of a therapeutic efficacy window. As demonstrated by Wang et al., the identity of the aglycone in substrate decoys influences their relative activity.^32^ Thus, the potential exists to develop substrate-based metabolic inhibitors of bacterial monosaccharides bearing different aglycones as a means to enhance potency. Overall, S-glycosides are promising novel compounds that act with specificity and selectivity to modulate bacterial glycosylation and fitness.

## Conclusions

Bacterial glycans are antibiotic targets and vaccine candidates with enormous untapped potential. This work describes novel metabolic inhibitors that disrupt bacterial glycan biosynthesis and fitness in pathogenic bacteria. The narrow effects of S-glycosides based on rare amino deoxy sugars are consistent with the apparent rare distribution of these epitopes and set the stage to further probe and perturb these structures. Broadly, this work expands the toolkit to interfere with bacterial glycans and gain critical insight into structure–function relationships.

## Methods

### Materials and Chemical Synthesis

Organic chemicals and anti-FLAG antibodies were purchased from Sigma-Aldrich. *H. pylori* strain G27^58^ was a gift of Manuel Amieva (Stanford University). *B. fragilis* (ATCC 23745) and AGS cells (ATCC CRL-1739) were purchased from ATCC and grown according to the supplier’s instructions. Ac_4_GlcNAc, Ac_4_GlcNAz, Ac_4_GalNAz, and Phos-FLAG were synthesized as previously described.^17,59,60^ BacSBn 4, DATSBn 5, and FucSBn 6 were synthesized by using standard organic chemistry procedures and characterized by standard techniques including ^1^H and ^13^C NMR spectroscopy and mass spectrometry. Analogues 4–6 were purified using flash silica gel chromatography.

### Metabolic Labeling

*H. pylori* cells were grown in rich liquid media supplemented with 0.5 mM^38^ Ac_4_GlcNAz, with 0.5 mM Ac_4_GlcNAz and 1–2 mM BacSBn 4, DATSBn 5, or FucSBn 6, or with 0.5 mM of the azide-free control Ac_4_GlcNAc for 4 days under microaerophilic conditions (14% CO_2_, 37 °C). *B. fragilis* were metabolically labeled with 0.5 mM Ac_4_GalNAz, with 0.5 mM Ac_4_GalNAz and 0.5–2 mM BacSBn 4, DATSBn 5, or FucSBn 6, or with 0.5 mM of the azide-free control Ac_4_GlcNAc for 2 days under anaerobic conditions (created by an Oxoid EZ anaerobe Gaspak in an airtight container; 37 °C). AGS cells were grown in Ham’s F12 Glutamax supplemented with 5 μM Ac_4_GalNAz, with 5 μM Ac_4_GalNAz and 10 μM mM BacSBn 4, DATSBn 5, or FucSBn 6, or with 5 μM azide-free control Ac_4_GlcNAc for 3 days in 5% CO_2_ at 37 °C. Cells were then harvested, rinsed with phosphate-buffered saline (PBS), and prepared for Western blot as described below.

### Western Blot

Following metabolic labeling, cells were lysed and resultant protein lysates were standardized (BioRad’s DC protein concentration assay) to a protein concentration of ∼2.5 mg mL^–1^ prior to reaction with 250 μM Phos-FLAG^42^ overnight at room temperature. Reacted lysates were loaded onto a 12% Tris–HCl SDS-PAGE gel, separated by electrophoresis, and transferred to nitrocellulose paper. Anti-FLAG-HRP was employed to visualize FLAG-tagged proteins via chemiluminescence.

### Lectin Binding

*H. pylori* were treated with 2 mM BacSBn 4, DATSBn 5, or FucSBn 6 or left untreated for 3 days and then were probed with Alexa Fluor 488-conjugated *Concanavalin A* (ConA). As a negative control, ConA was preincubated with 400 mM mannose (carbo-block) prior to binding to untreated *H. pylori*. Cells were analyzed by flow cytometry on a BD Accuri C6^+^ (BD Biosciences) instrument, with 10,000 live cells gated for each replicate experiment. Data were analyzed by using FlowJo software (Ashland, OR).

### Growth Curves

Bacterial growth was monitored during log phase and until bacteria reached stationary phase—over the course of 8 days for *H. pylori* and over 2 days for *B. fragilis*. Cells were inoculated into rich liquid media at a starting OD_600_ of ∼0.1 and cultured in the absence of S-glycoside or the presence of 2 mM BacSBn 4, 1 mM DATSBn 5, or 2 mM FucSBn 6 at 37 °C with gentle shaking under microaerophilic or anaerobic conditions for *H. pylori* and *B. fragilis*, respectively. The OD_600_ was measured at the indicated time points by using a SPECTROStar Nano 96-well plate reader (Thermo Fisher Scientific).

### *H. pylori* Viability Measurements

*H. pylori* was standardized to an OD_600_ of 0.4 in rich media and incubated for 4 days in the absence of S-glycoside or presence of 2 mM BacSBn 4, 1 mM DATSBn 5, or 2 mM FucSBn 6. Cells were analyzed prior to incubation and after 4 days of incubation using the LIVE/DEAD BacLight Bacterial Viability and Counting Kit (Invitrogen) according to manufacturer’s instructions. Following staining with propidium iodide and SYTO 9 dyes included in the kit, cells were analyzed by flow cytometry using a BD Accuri C6^+^ (BD Biosciences, San Jose, California) instrument, with 10,000 live cells gated for each replicate. The number of live and dead *H. pylori* cells were counted using FlowJo software to determine the percentage of live *H. pylori* (% live = 100*[(# live cells)/(# live cells + # dead cells)]).

### Motility Assays

*H. pylori* were treated with S-glycosides or left untreated, and then their ability to swarm was monitored over the course of 13 days. *H. pylori* cultures were standardized to an OD_600_ between 0.3 and 0.4 in rich media and then incubated with no S-glycoside or with 2 mM BacSBn 4, 1 mM DATSBn 5, or 2 mM FucSBn 6 under microaerophilic conditions. Cells from each culture were concentrated by centrifugation and resuspended in rich media, and then 10 μL concentrated culture was plated onto soft agar plates supplemented with 4% agar and 10% fetal bovine serum. Plates were incubated at 37 °C and 14% CO_2_, and the colony diameter was measured for 13 days and imaged on day 13.

### Biofilm Formation Assays

The ability of *H. pylori* to form a biofilm in the absence or presence of thioglycosides was assessed following O’Toole’s literature protocol.^44^ Bacteria were standardized to an OD_600_ of 0.3 to 0.4 in rich liquid media in the absence of S-glycoside or with 2 mM BacSBn 4, 1 mM DATSBn 5, or 2 mM FucSBn 6, and samples were added in triplicate to the side wells of a 96-well plate. The bacteria were incubated for 5 days at 37 °C and 14% CO_2_. After incubation, the medium was carefully removed, and the biofilm was stained with 0.15% crystal violet. Side-view images of the triplicate wells were taken after staining to visualize biofilm production. The stained wells were then solubilized in 30% acetic acid in water, and the absorbance of the solution was quantified at 550 nm using a SPECTROstar Nano plate reader (Thermo Fisher Scientific).

### AGS Cell Count and Viability

AGS cells were seeded at a density of 1 × 10^5^ cells/mL in 1 mL of Ham’s F12 Glutamax medium and cultured for 3 days in the absence or presence of 10 μM S-glycosides in a 48-well tissue culture plate. After 3 days, the media were aspirated, and 0.25% Trypsin and EDTA was added to each well for 5 min at 37 °C. This reaction was stopped by the addition of AGS media. To assess cell viability in the absence or presence of an inhibitor, Gibco Trypan blue stain (0.4%) was added to the cell suspension at a 1:1 ratio, and the cells were counted using a hemocytometer (Countess 3 Invitrogen). This analysis yielded the number of cells/mL and the % live cells.

## Supporting Information Available

Full experimental details, compound characterization data, and protein loading controls, mean fluorescence intensities of samples from flow cytometry analyses, and quantification of motility and biofilm (Figures S1 and S2) (PDF)

## Abbreviations

S-glycoside: thioglycoside
LPS: lipopolysaccharide
CPS: capsular polysaccharide
D-Bac: di-*N*-acetyl D-bacillosamine
D-DATDG: D-2,4-diacetamido-2,4,6-trideoxy galactose
D-FucNAc: *N*-acetyl d-fucosamine
PglD: UDP-amino-sugar acetyltransferase
LgtC: α-1,4,-galactosyltransferase
UDP: uridine diphosphate
MOE: metabolic oligosaccharide engineering
*H. pylori*: *Helicobacter pylori*
*B. fragilis*: *Bacteroides fragilis*
Ac_4_GlcNAc: peracetylated *N*-acetylglucosamine
Ac_4_GlcNAz: peracetylated *N*-azidoacetylglucosamine
Ac_4_GalNAz: peracetylated *N*-azidoacetylgalactosamine
SBn: thiobenzyl
NO_2_^–^: nitrite
TBAN_3_: tetrabutyl ammonium azide
NIS: *N*-iodosuccinimide
TfOH: triflic acid
NBS: *N*-bromosuccinimide
THF: tetrahydrofuran
H_2_O: water
CCl_3_CN: trichloroacetonitrile
CH_2_Cl_2_: methylene chloride
NHAc: *N*-acetyl
AcOH: acetic acid
Phos-FLAG: phosphine–FLAG conjugate
ConA: concanavalin A
OD_600_: optical density at 600 nm
K_2_CO_3_: potassium carbonate
AGS: gastric adenocarcinoma
ATCC: American Type Culture Collection
PBS: phosphate-buffered saline
NMR: nuclear magnetic resonance
HRP: horse radish peroxidase
EDTA: ethylenediaminetetraacetic acid
