# Supplementary material for: Thioglycosides Act as Metabolic Inhibitors of Bacterial Glycan Biosynthesis
Source: ACS Infect Dis. 2023 Sep 12;9(10):2025–35. doi: 10.1021/acsinfecdis.3c00324 (PMC10580310; doi:10.1021/acsinfecdis.3c00324)
Supplement: Supplementary file 1 — id3c00324_si_001.pdf [file id3c00324_si_001.pdf]

Supporting Information

Thioglycosides act as metabolic inhibitors of bacterial glycan biosynthesis

Isabella de la Luz Quintana,[1#] Ankita Paul,[2#] Aniqa Chowdhury,[1] Karen D. Moulton,[1] Suvarn S. Kulkarni,[2*] and Danielle H. Dube[1*]

[1]Department of Chemistry & Biochemistry, Bowdoin College,
6600 College Station, Brunswick, Maine 04011
[2]Department of Chemistry, Indian Institute of Technology Bombay,
Powai, Mumbai 400076, India

Corresponding author: ddube@bowdoin.edu, suvarn@chem.iitb.ac.in

Table of Contents

Chemistry……………………………………………… *s*-3
General……………………………………………… *s*-3
Compound **9**……………………………………………… *s*-3
Compound **4**……………………………………………… *s*-4
Compound **11**……………………………………………… *s*-5
Compound **5**……………………………………………… *s*-6
Compound **13**……………………………………………… *s*-7
Compound **6**……………………………………………… *s*-7

Biology……………………………………………… *s*-8
General……………………………………………… *s*-8
Bacterial and cell culture growth conditions……………………………… *s*-8
Metabolic labeling of *H. pylori*……………………………………… *s*-9
SDS-PAGE and Western blot analysis of azide-labeled glycans…………… *s*-9
Lectin binding flow cytometry experiments with *H. pylori*………………… *s*-10
Growth curve for *H. pylori*……………………………………… *s*-10
H. pylori viability measurements……………………………………… *s*-11
Motility assay……………………………………………… *s*-11
Biofilm formation assay……………………………………… *s*-11
Metabolic labeling of *B. fragilis*……………………………………… *s*-12
Growth curve for *B. fragilis*……………………………………… *s*-12
Metabolic labeling of AGS cells……………………………………… *s*-12
AGS cell count and viability……………………………………… *s*-12

Supplemental Figures……………………………………… *s*-14
Figure S1……………………………………………… *s*-14
Figure S2……………………………………………… *s*-15

Supplemental References……………………………………………………………………*s*-16

NMRs and MSs...………………………………………………………...……………………*s*-17

Chemistry

General:

All reactions were conducted under the dry nitrogen atmosphere. Solvents (CH_2Cl_2 >99%, THF 99.5%, acetonitrile 99.8%, DMF 99.5%) were purchased in capped bottles and dried under sodium or CaH_2. All other solvents and reagents were used without further purification. Glassware was oven dried before use. Reactions under heating condition were performed using paraffin oil bath. TLC was performed on pre-coated Aluminium plates of Silica Gel 60 F254 (0.25 mm, E. Merck). Developed TLC plates were visualized under a short-wave UV lamp and by heating plates that were dipped in ammonium molybdate/cerium (IV) sulphate solution. Silica gel column chromatography was performed using Silica Gel (100-200 mesh, as well as 230-400 mesh) and employed a solvent polarity correlated with TLC mobility. We have used 3 Angstrom powdered molecular sieves in our study. The powdered MS were weighed in a dried pear-shaped flask and activated by periodic heating of flask by using flame over a period of 15 minutes. NMR experiments were conducted on 500 and 400 MHz instrument using $CDCl_3$ (D, 99.8%), CD_3OD (D, 99.8%) or D_2O (D, 99.9%) as solvents. Chemical shifts are relative to the deuterated solvent peaks and are in parts per million (ppm). Structural assignments were made with additional information from gCOSY and gHSQC experiments. gCOSY was used to confirm proton assignments and gHSQC experiment was done to confirm proton carbon correlation. In the 1H NMR spectrum, data are reported as follows: chemical shift (δ ppm), multiplicity (s: singlet, d: doublet, dd: doublet of doublet, t: triplet, q: quartet, m: multiplet, bs: broad singlet and ABq: AB quartet), coupling constant (J in Hz), integration and respective assigned proton(s). Mass spectra were acquired in the ESI-TOF mode. Specific rotation experiments were measured at 589 nm (Na) and 25 °C. IR spectra were recorded on an FT-IR spectrometer.

Compound 9:

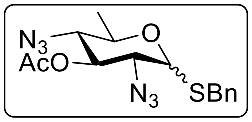

NBS (0.118 g, 0.663 mmol) was added to the stirred solution of compound **8** (0.077 g, 0.221 mmol) in 3 mL THF/H_2O (5:1) at 0 °C. After 15 min, the reaction mixture was diluted with EtOAc and washed with aqueous $Na_2S_2O_3$ solution. The separated organic layer was dried over anhydrous

Na_2SO_4, filtered, and concentrated. The crude compound was washed with hexane to remove the non-polar impurity and taken to next step.

Crude imidate donor (0.088 g, 0.221 mmol), benzyl mercaptan (0.5 mL, 0.442 mmol) and molecular sieves (3Å, 100 mg) were dissolved in dry CH_2Cl_2 (3 mL). Then, TfOH (6 µl, 0.066 mmol) was added dropwise to the solution at 0 ºC. After 2 h, with completion of reaction, mixture was quenched by Et_3N and molecular sieves were filtered out through Celite. Then crude was concentrated and purified by flash column chromatography to afford compound **9** (0.048 g, 61%) in *α*:*β* (10:1) as yellow viscous liquid.

$[\alpha]_D^{25}$ +7.80 (c = 0.1, $CHCl_3$).

IR (cm^{-1}, $CHCl_3$) *ν* 2927, 2110, 1724, 1463, 1039, 758.

^{1}H NMR (400 MHz, $CDCl_3$) *δ* 7.34-7.27 (m, 5H, ArH), 5.28 (t, 1H, *J* = 9.6, H-3), 5.19 (d, 1H, *J* = 5.6, H-1), 4.09-4.05 (m, 1H, H-5), 3.86 (q, 1H, *J* = 10.4, H-4), 3.75, 3.72 (ABq, 2H, *J* = 13.6 Hz, CH_2S), 3.14 (t, 1H, *J* = 9.6 Hz, H-2), 2.18 (s, 3H, OAc), 1.30 (d, 3H, *J* = 6 Hz, H-6).

^{13}C NMR (100 MHz, $CDCl_3$) *δ* 169.59, 137.10, 129.01, 128.61, 127.34, 81.76, 72.22, 66.79, 66.44, 61.87, 34.18, 20.72, 18.16.

HRMS (ESI-TOF) (m/z): $[M+H]^+$ calcd. for $C_{15}H_{19}N_6O_3S$ 363.1103; found, 363.1102.

Compound 4:

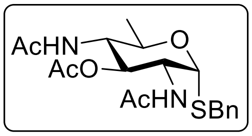

To compound **9** (0.031 g, 0.085 mmol) in THF (2 mL), activated Zn dust (53 mg) was added followed by dropwise addition of AcOH (0.3 mL) at rt. Mixture was allowed to stir at same temperature for 8 h. After complete conversion of azide to amine, zinc was filtered through celite pad, concentrated and dried under high *vacuum* for 30 min.

Crude di-amine compound was dissolved in THF (1 mL). To the clear solution, Ac_2O (0.03 mL, 0.336 mmol), Et_3N (0.03 mL) and DMAP (1 mg, 0.008 mmol) were added sequentially at 0 °C, mixture was allowed to stir at room temperature for 2 h. After completion of reaction, solvents were removed in *vacuo* and the crude product was purified by column chromatography over silica gel (70% ethyl acetate: pet ether) to furnish desired substrate **4** as yellow viscous liquid (0.025 g, 75%, *α*:*β* =15:1).

[α]$_D^{25}$ +35.50 (c = 0.2, $CHCl_3$).

IR (cm^{-1}, $CHCl_3$) *v* 3031, 2524, 1722, 1364, 1227, 1045, 753.

^{1}H NMR (400 MHz, $CDCl_3$) *δ* 7.31-7.25 (m, 5H, ArH), 6.00-5.98 (m, 2H, NH), 5.37 (d, 1H, J = 5.2 Hz, H-1), 4.88 (d, 1H, J = 10.8 Hz, H-3), 4.54-4.47 (m, 1H, H-5), 4.05-3.96 (m, 2H, H-2, H-4), 3.79, 3.77 (ABq, 2H, *J* = 12.8 Hz, CH_2S), 2.01 (s, 3H, OAc), 1.90 (s, 3H, NHAc), 1.86 (s, 3H, NHAc), 1.16 (d, 3H, *J* = 5.6 Hz, H-6).

^{13}C NMR (100 MHz, $CDCl_3$) *δ* 172.24, 170.04, 169.97, 137.55, 128.85, 128.70, 127.45, 84.08, 71.76, 68.19, 55.04, 55.23, 35.27, 23.16, 23.04, 20.85, 17.73.

HRMS (ESI-TOF) (m/z): $[M+Na]^+$ calcd. for $C_{19}H_{26}N_2NaO_5S$ 417.1467; found, 417.1467.

Compound 11:

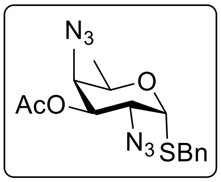

NBS (0.573 g, 3.219 mmol) was added to the stirred solution of compound **10** (0.373 g, 1.073 mmol) in 4 mL THF/H_2O (5:1) at 0 °C. After 15 min, the reaction mixture was diluted with EtOAc and washed with aqueous $Na_2S_2O_3$ solution. The separated organic layer was dried over anhydrous Na_2SO_4, filtered, and concentrated. The crude compound was washed with hexane to remove the non-polar impurity and taken to next step.

Crude imidate donor (0.429 g, 1.073 mmol), benzyl mercaptan (0.2 mL, 2.146 mmol) and molecular sieves (3Å, 200 mg) were dissolved in dry CH_2Cl_2 (5 mL). Then, TfOH (28 µl, 0.322 mmol) was added dropwise to the solution at 0 ºC. After 2 h, with completion of reaction, mixture was quenched by Et_3N and molecular sieves were filtered out through Celite. Then crude was concentrated and purified by flash column chromatography to afford compound **11** (0.291 g, 76%, *α*:*β* =20:1) as sticky colourless gum.

[α]$_D^{25}$ +4.60 (c = 0.1, $CHCl_3$).

IR (cm^{-1}, $CHCl_3$) *v* 2924, 2111, 1720, 1582, 1271, 1034, 745, 694.

^{1}H NMR (400 MHz, $CDCl_3$) *δ* 7.34-7.27 (m, 5H, ArH), 5.23 (d, 1H, *J* = 5.6 Hz, H-1), 5.18 (dd, 1H, *J* = 10.7 Hz, 3.6 Hz, H-3), 4.42-4.40 (m, 1H, H-5), 4.25 (dd, 1H, *J* = 10.8 Hz, 5.6 Hz, H-2), 3.94 (dd, 1H, *J* = 3.6 Hz, 1.2 Hz, H-4), 3.75, 3.69 (ABq, 2H, *J* = 13.6 Hz, CH_2S), 2.19 (s, 3H, OAc), 1.24 (d, 3H, *J* = 6.4 Hz, H-6).

^{13}C NMR (100 MHz, $CDCl_3$) δ 169.97, 137.25, 129.02, 128.60, 127.29, 82.06, 73.07, 65.15, 63.74, 57.67, 34.10, 20.57, 17.08.
HRMS (ESI-TOF) (m/z): $[M]^+$ calcd. for $C_{15}H_{18}N_6O_3S$ 362.1260; found, 362.1260.

Compound 5:

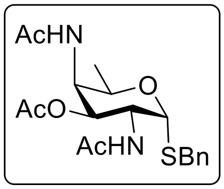

To compound **11** (0.150 g, 0.414 mmol) in THF (5 mL), activated Zn dust (258 mg) was added followed by dropwise addition of AcOH (1.5 mL) at rt. Mixture was allowed to stir at same temperature for 8 h. After complete conversion of azide to amine, zinc was filtered through celite pad, concentrated and dried under high *vacuum* for 30 min.

Crude di-amine compound was dissolved in THF (3 mL). To the clear solution, Ac_2O (0.2 mL, 1.656 mmol), Et_3N (0.2 mL) and DMAP (5 mg, 0.1 mmol) were added sequentially at 0 °C, mixture was allowed to stir at room temperature for 4 h. After completion of reaction, solvents were removed in *vacuo* and the crude product was purified by column chromatography over silica gel (50% ethyl acetate: pet ether) to furnish desired substrate **5** as brown viscous liquid (0.132 g, 81% yield , α:β =20:1).
$[\alpha]_D^{25}$ +41.70 (c = 0.4, $CHCl_3$).
IR (cm^{-1}, $CHCl_3$) ν 3378, 2946, 2835, 1723, 1652, 1252, 1110, 1024, 920.
^1H NMR (400 MHz, $CDCl_3$) δ 7.32-7.27 (m, 5H, ArH), 6.55 (d, 1H, J = 10.0 Hz, NH), 6.31 (d, 1H, J = 8.4 Hz, NH), 5.41 (s, 1H, H-1), 5.00 (dd, 1H, J = 11.6 Hz, 4.0 Hz, H-3), 4.50-4.44 (m, 3H, H-2, H-4, H-5), 3.77 (s, 2H, CH_2S), 2.08 (s, 3H, OAc), 2.00 (s, 3H, NHAc), 1.91 (s, 3H, NHAc), 1.07 (d, 3H, J = 6.4 Hz, H-6).
^{13}C NMR (100 MHz, $CDCl_3$) δ 171.38, 170.50, 137.57, 128.79, 128.65, 127.38, 84.51, 69.57, 66.04, 50.75, 48.48, 35.38, 23.20, 22.64, 20.93, 16.41.
HRMS (ESI-TOF) (m/z): $[M+Na]^+$ calcd. for $C_{19}H_{26}N_2NaO_5S$ 417.1457; found, 417.1456.

Compound 13:

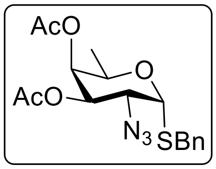

NBS (0.121 g, 0.681 mmol) was added to the stirred solution of compound **12** (0.083 g, 0.227 mmol) in 3 mL THF/H_2O (5:1) at 0 °C. After 15 min, the reaction mixture was diluted with EtOAc and washed with aqueous $Na_2S_2O_3$ solution. The separated organic layer was dried over anhydrous Na_2SO_4, filtered, and concentrated. The crude compound was washed with hexane to remove the non-polar impurity and taken to next step.

Crude imidate donor (0.094 g, 0.227 mmol), benzyl mercaptan (0.05 mL, 0.454 mmol) and molecular sieves (3Å, 100 mg) were dissolved in dry CH_2Cl_2 (3 mL). Then, TfOH (6 µl, 0.068 mmol) was added dropwise to the solution at 0 ºC. After 2 h, with completion of reaction, mixture was quenched by Et_3N and molecular sieves were filtered out through Celite. Then crude was concentrated and purified by flash column chromatography (20% ethyl acetate: pet ether) to afford compound **13** (0.059 g, 70%, α:β = 10:1) as yellow sticky gum.

$[\alpha]_D^{25}$ +3.10 (c = 0.2, $CHCl_3$).

IR (cm^{-1}, $CHCl_3$) ν 3070, 2858, 2110, 1750, 1561, 1440, 1049, 823, 702.

^{1}H NMR (400 MHz, $CDCl_3$) δ 7.36-7.26 (m, 5H, ArH), 5.29-5.28 (m, 2H, H-1, H-4), 5.14 (dd, 1H, J = 10.8 Hz, 3.2 Hz, H-3), 4.48-4.37 (m, 1H, H-5), 4.20- 4.13 (m, 1H, H-2), 3.78, 3.72 (ABq, 2H, J = 13.6 Hz, CH_2S), 2.17 (s, 3H, OAc), 2.04 (s, 3H, OAc), 1.10 (d, 3H, J = 6.0 Hz, H-6).

^{13}C NMR (100 MHz, $CDCl_3$) δ 170.32, 169.63, 137.36, 128.99, 128.58, 127.25, 82.25, 70.82, 70.44, 65.27, 57.75, 34.19, 20.62, 20.61, 15.84.

HRMS (ESI-TOF) (m/z): $[M+H]^+$ calcd. for $C_{17}H_{22}N_3O_5S$ 380.1274; found, 380.1273.

Compound 6:

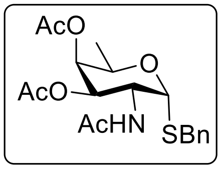

To compound **13** (0.060 g, 0.158 mmol) in THF (3 mL), activated Zn dust (98 mg) was added followed by dropwise addition of AcOH (0.5 mL) at rt. Mixture was allowed to stir at same

temperature for 8 h. After complete conversion of azide to amine, zinc was filtered through celite pad, concentrated and dried under high *vacuum* for 30 min.

Crude amine compound was dissolved in THF (1 mL). To the clear solution, Ac_2O (0.04 mL, 0.474 mmol), Et_3N (0.04 mL) and DMAP (2 mg, 0.016 mmol) were added sequentially at 0 °C, mixture was allowed to stir at room temperature for 2 h. After completion of reaction, solvents were removed in *vacuo* and the crude product was purified by column chromatography over silica gel (40% ethyl acetate: pet ether) to furnish desired substrate **6** as yellow viscous liquid (0.048 g, 77%, α:β =20:1).

$[\alpha]_D^{25}$ +17.07 (c = 0.1, $CHCl_3$).

IR (cm^{-1}, $CHCl_3$) ν 2945, 2553, 2071, 1725, 1246, 1025, 920.

^{1}H NMR (400 MHz, $CDCl_3$) δ 7.31-7.27 (m, 5H, ArH), 5.71 (bs, 1H, NH), 5.38 (d, 1H, J = 5.2 Hz, H-1), 5.19 (d, 1H, J = 2.8 Hz, H-4), 5.04 (dd, 1H, J = 12 Hz, 3.6 Hz, H-3), 4.75-4.69 (m, 1H, H-5), 4.39 (q, 1H, J = 6.4 Hz, H-2), 3.77, 3.75 (ABq, 2H, J = 13.2 Hz, CH_2S), 2.167 (s, 3H, OAc), 1.98 (s, 3H, OAc), 1.87 (s, 3H, NHAc), 1.07 (d, 3H, J = 5.6 Hz, H-6).

^{13}C NMR (100 MHz, $CDCl_3$) δ 171.07, 170.67, 170.12, 137.65, 128.83, 128.64, 127.34, 84.48, 70.39, 69.12, 65.86, 47.98, 35.24, 23.18, 20.75, 20.69, 15.92.

HRMS (ESI-TOF) (m/z): $[M+H]^+$ calcd. for $C_{19}H_{26}NO_3S$ 396.1474; found, 396.1473.

Biology

General. All biological reagents were obtained from commercial suppliers (e.g., Millipore Sigma, Fisher, ThermoScientific, Bio-Rad, Click Chemistry Tools) and used without further purification. *Helicobacter pylori* strain G27[1] was a gift from Manuel Amieva (Stanford University). *B. fragilis* (ATCC 23745) and *Homo sapiens* gastric adenocarcinoma (AGS, ATCC number: CRL-1739) cells were purchased from ATCC and grown according to the supplier's instructions.

Bacterial and cell culture growth conditions. *Helicobacter pylori* strain G27 was streaked from frozen stocks onto horse blood agar plates (4% Columbia agar, 5% horse blood, 10 mg/ml vancomycin, 5 μg/ml cefsulodin, 0.3 μg/ml polymyxin B, 5 μg/ml trimethoprim, and 8 μg/ml amphotericin B) and grown for 3-4 days under microaerophilic conditions (14% CO_2, 37 °C). Liquid cultures of *H. pylori* were grown in brucella broth (Brucella broth pH 7.0; 10% fetal bovine

serum, 10 mg/ml vancomycin). *Bacteroides fragilis* strain ATCC 23745 was grown on brain-heart infusion plates (1.5% Bacto agar, 3.7% brain-heart infusion media, 0.5% yeast extract, 15 µg/mL hematin porcine) for 2 days under anaerobic conditions (generated by a Thermo Scientific (Waltham, Massachusetts) Oxoid AnaeroGen Sachet in an airtight container, 37 °C). Liquid cultures of *B. fragilis* were grown in BHI broth (3.7% brain-heart infusion media). *Homo sapiens* gastric adenocarcinoma (AGS, ATCC number: CRL-1739) cells were cultured in AGS media (Ham's F-12K Glutamax with 10% Fetal Bovine Serum) in a T-75 flask for 3 days at 5% CO_2, 37 °C. Upon roughly 95% confluence, cells were rinsed with phosphate buffered saline (PBS) then trypsinized (0.25% trypsin-EDTA) for 5 mins at 5% CO_2, 37 °C. Once detached, cells were quenched 1:5 with AGS media before subsequent metabolic labeling and viability screening.

Metabolic labeling of *H. pylori*. *H. pylori* from a frozen stock was streaked onto agar plates using a sterile tip applicator and then incubated in brucella broth under microaerophilic conditions (see *Bacterial and cell growth conditions*). After 3-4 days of growth on plates, *H. pylori* were inoculated at an OD_{600} of 0.1-0.4 in liquid media supplemented with 0.5 mM of Ac_4GlcNAc, 0.5 mM of Ac_4GlcNAz, or 0.5 mM Ac_4GlcNAz and varying concentrations (0.5 mM - 2 mM) of thioglycosides **4-6**. After metabolic labeling for 4 days in liquid media under microaerophilic conditions (14% CO_2, 37 °C), *H. pylori* were centrifuged at 3500 rpm using an Eppendorf 5804R centrifuge and washed three times with PBS.

SDS-PAGE and Western blot analysis of azide-labeled glycans. To probe for azide-labeled glycans produced by cells, metabolically labeled and rinsed cells were lysed in lysis buffer (20 mM Tris-HCl, pH 7.4, 1% Igepal, 150 mM NaCl, 1 mM EDTA) containing protease inhibitor cocktail (Sigma Aldrich, St. Louis, MO) for 30 minutes at -20 °C. Lysates were pelleted at 10,000 x *g* using an Eppendorf microcentrifuge. *B. fragilis* lysates underwent additional lysing steps in which they were freeze thawed and sonicated in an ultrasonic bath (1.9 L, FisherScientific) for 20 minutes at room temperature. The protein concentrations of supernatants were measured using the DC Protein Assay (Bio-Rad, Hercules, CA) and standardized to equal concentrations (~2.5 mg/mL). Standardized samples were subsequently treated 1:1 with 500 µM Phos-FLAG and reacted at room temperature with shaking overnight, then analyzed by SDS-PAGE and Western blot. In preparation for electrophoresis, reacted samples were combined in a 1:1 ratio with 2X SDS reducing loading buffer and boiled at 95 °C for 5-10 minutes. EZ-Run protein ladder (15 µg, Fisher

Scientific) and 15 µg or 37.5 µg of samples, respectively, for Western blot analysis and Coomassie staining were loaded onto two separate gels (12% Tris-HCl SDS-PAGE gels with a 4% stacking layer). Gels were electrophoresed at 200V for 60 minutes in 1X SDS running buffer (H_2O, 3.47 mM SDS, 24.71 mM Tris base, 191.95 mM glycine) in a Mini PROTEAN Tetra Cell (Bio-Rad, Hercules, CA). After electrophoresis, proteins were transferred to a nitrocellulose membrane (Bio-Rad – Amersham, GE Healthcare Life Sciences) at 100 V for 1 hour or stained with Coomassie (Stain: 45% deionized water, 45% Methanol, 10% acetic acid, 0.25% Coomassie brilliant blue/Destain: 50% deionized water, 40% methanol, 10% acetic acid) to visualize equal protein loading. Immunoblots were blocked for 1 hour with 5% non-fat dried milk in 0.05% TBS-T buffer (5 mM Tris-HCl, 0.05% Tween-20 (BioRad), pH 7.4). Anti-FLAG-HRP (Sigma Aldrich; 1:1000 dilution in blocking buffer) was employed to visualize FLAG-tagged proteins via chemiluminescence (SuperSignal West Pico Chemiluminescent Substrate) with the G:BOX Chemi XRQ gel documentation system (Syngene).

Lectin binding flow cytometry experiments with *H. pylori*. *H. pylori* were cultured for 3 days in rich liquid media supplemented with 2 mM of thioglycosides **4-6** or without any additional supplement (untreated), then thoroughly washed with 1X PBS prior to incubation with Alexa Fluor 488-conjugated *Concanavilin A* (ConA) lectin (15 µg/ml in 1X PBS; Thermo Fischer, Waltham, MA; ex: 488/em: 519) for 45 mins at 37 °C in 14% CO_2. As a control, ConA was pre-incubated with 400 mM mannose for 60 mins at 37 °C prior to binding to untreated *H. pylori*. Cells were then washed three times with 1X PBS and analyzed by flow cytometry using a BD Accuri C6 (BD Biosciences) instrument, with 10,000 live cells gated for each replicate experiment. Labeling was performed in triplicate and is reported as number of cells versus fluorescence intensity in histogram plots. Alternatively, flow cytometry data are reported as the mean fluorescence intensity (MFI) of a population of cells from replicate experiments, as calculated using FlowJo software (Ashland, OR).

Growth curve for *H. pylori*. *H. pylori* strain G27 was grown on horse blood agar plates (4% Columbia agar, 5% horse blood, 10 mg/ml vancomycin, 5 µg/ml cefsulodin, 0.3 µg/ml polymyxin B, 5 µg/ml trimethoprim, and 8 µg/ml amphotericin B) for 3-4 days under microaerophilic conditions (14% CO_2, 37°C). Cells were inoculated into liquid brucella broth to a starting OD_{600} of approximately 0.1. Liquid cultures (3.5 mL) in the absence or presence of thioglycosides **4-6** (1-2

mM) were incubated under microaerophilic conditions with gentle shaking. The OD_{600} of each culture was measured daily using a SPECTROStar Nano 96 well plate reader (ThermoFisher Scientific) for 8 days.

***H. pylori* viability measurements.** The viability of untreated or thioglycoside treated *H. pylori* was assessed over the course of 4 days by scoring percent of live cells. Bacterial cells were standardized to an OD_{600} of 0.4 in brucella broth and incubated for 4 days in the absence or presence of 1-2 mM of S-glycosides **4-6**. Viability was scored on days 0 and 4 using the LIVE/DEAD BacLight Bacterial Viability and Counting Kit (Invitrogen). To establish controls, *H. pylori* cells were resuspended in either 0.85% NaCl or 70% Isopropanol, incubated for 5 min at 37 °C, and rinsed with 0.85% NaCl before staining with LIVE/DEAD BacLight Bacterial Viability solution (Invitrogen), consisting of propidium iodide and SYTO 9. The cells were incubated in the dark for 15 minutes then analyzed by flow cytometry using a BD Accuri C6 (BD Biosciences, San Jose, California) instrument, with 10,000 live cells gated for each replicate. Inhibitor treated cells were scored as live or dead by using gates established with live and dead controls. The number of live and dead *H. pylori* cells were counted using FlowJo software (Ashland, OR) to determine the percentage of live *H. pylori* (% live = 100*[(# live cells)/(# live cells + # dead cells)]).

Motility assay. *H. pylori* cultures were standardized to an OD_{600} between 0.3 to 0.4 in brucella broth, then incubated in the absence or presence of thioglycosides **4-6** (1-2 mM) under microaerophilic conditions. Cells from each culture were concentrated by centrifugation, resuspended in 100 µL brucella broth, then 10 µL concentrated culture was plated onto soft agar (brucella broth with 10% (v/v) fetal bovine serum, 6 µg/ml vancomycin, and 0.4% Difco agar). Plates were incubated in microaerophilic conditions, measuring colony diameter daily for 13 days.

Biofilm formation assay. The ability of *H. pylori* to form biofilm in the absence or presence of thioglycosides was assessed following O'Toole's literature protocol.[2] Bacteria were standardized to an OD_{600} of 0.3 to 0.4 in rich liquid media in the absence or presence of thioglycosides **4-6** (1-2 mM), and samples were added in triplicate to the side wells of a 96-well plate. The bacteria were incubated for 2-5 days depending on the doubling rate of the organism. After incubation, media was carefully removed, and biofilm was stained with 0.15% crystal violet to visualize. Pictures were taken of the stained wells for visual qualification of biofilm production. The stained wells

were then solubilized in 30% acetic acid in water, and the absorbance of the solution was quantified at 550 nm using a SPECTROstar Nano plate reader (ThermoFisher Scientific).

Metabolic labeling of *B. fragilis.* *B. fragilis* from a frozen stock was streaked onto agar plates using a sterile tip applicator and then incubated in liquid broth under anaerobic conditions (see *Bacterial and cell growth conditions*). After overnight growth on plates, *B. fragilis* were inoculated at an OD_{600} of 0.1-0.4 in liquid media supplemented with 0.5 mM of $Ac_4GlcNAc$, 0.5 mM of $Ac_4GalNAz$, or 0.5 mM $Ac_4GalNAz$ and varying concentrations (0.5 - 2 mM) of thioglycosides **4-6**. After metabolic labeling for 2 days in liquid media under anaerobic conditions (created by a Thermo Scientic Oxoid AnaeroGen Sachet in an airtight container; 37°C), *B. fragilis* were centrifuged at 3500 rpm using a Sorvall Legend RT^+ centrifuge (Thermo Scientific, Waltham, MA) and washed three times with PBS.

Growth curve for *B. fragilis.* *B. fragilis* strain ATCC 23745 was grown on brain heart infusion plates (1.5% Bacto agar, 3.7% brain-heart infusion media, 0.5% yeast extract, 15 µg/mL hematin porcine) for 2 days under anaerobic conditions (generated by a Thermo Scientific Oxoid AnaeroGen Sachet in an airtight container, 37°C). Cells were inoculated into liquid BHI broth to a starting OD_{600} of approximately 0.1. Liquid cultures (3.5 mL) in the absence or presence of 2 mM Bac-SBn **4**, DAT-SBn **5**, and Fuc-SBn **6** were incubated with shaking under anaerobic conditions. The OD_{600} of each culture was measured at the indicated time points over approximately 2 days using a SPECTROStar Nano 96 well plate reader (ThermoFisher Scientific).

Metabolic labeling of AGS cells. AGS cells were seeded at a density of $4x10^5$ cells/mL in 2 mL of liquid culture in a 6-well tissue culture plate. Cells were treated with a negative control (5 µM $Ac_4GlcNAc$) or metabolically labeled with 5 µM $Ac_4GalNAz$, in the absence or presence of 10 µM thioglycosides **4-6** for 3 days at 5% CO_2, 37°C. Following metabolic labeling, mammalian cells were probed for glycosylation inhibition via western blot.

AGS cell count and viability. AGS cells were seeded at a density of $1x10^5$ cells/mL in 1 mL of liquid culture and cultured for 3 days in the absence or presence of thioglycosides **4-6** (10 µM) in a 48-well tissue culture plate. After 3 days, the supernatants were aspirated, and 0.25% Trypsin and EDTA was added to each well for 5 minutes at 37 °C. This reaction was stopped by the addition

of AGS media. To assess cell viability in the absence or presence of inhibitor, Gibco Trypan blue stain (0.4%) was added to the cell suspension at a 1:1 concentration, and the cells were counted using a hemocytometer (Countess 3 Invitrogen).

Supplemental Figures

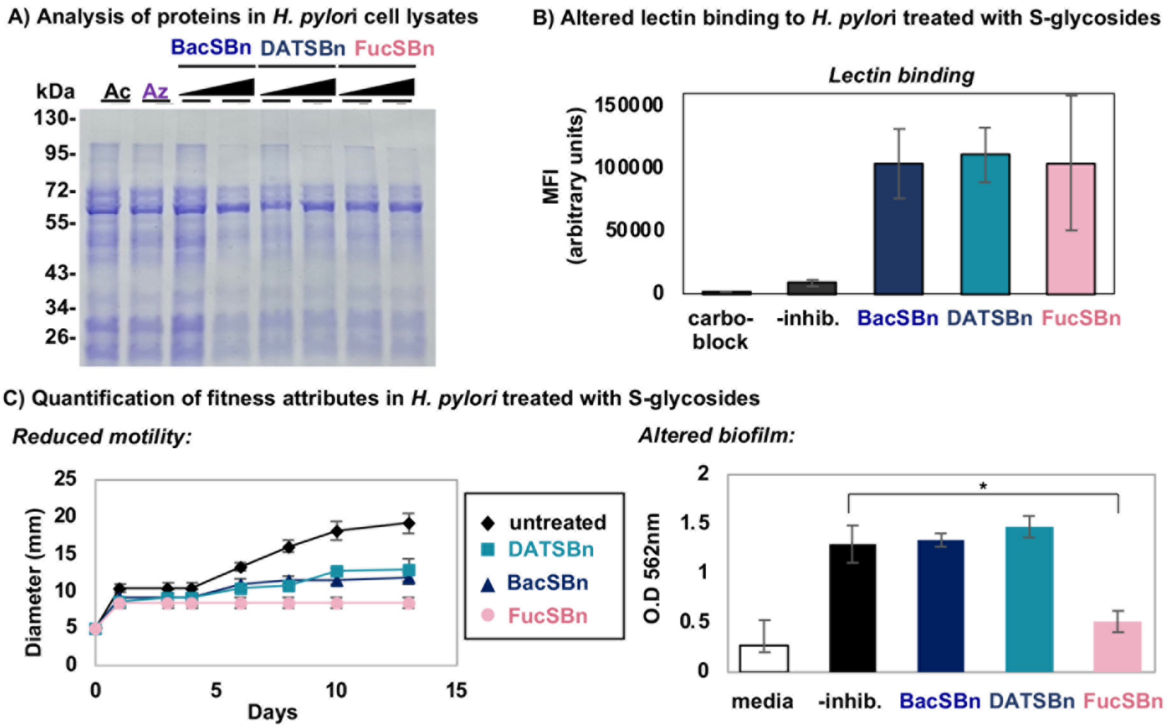

Figure S1. Protein loading and quantification of lectin binding and fitness attributes for samples presented in Figure 3. A) Coomassie staining of electrophoresed samples from Figure 3A revealed that Western samples contained protein. B) Mean fluorescence intensities from flow cytometry analyses from triplicate samples analyzed in Figure 3A reveal shifts in green fluorescence intensity, corresponding to ConA lectin binding, for thioglycoside-treated samples relative to the untreated control (-inhib.). Data shown are representative of replicate experiments. C) Quantification of fitness attributes in *H. pylori* treated with thioglycosides indicates that motility and biofilm are impacted. Measurement of colony diameter daily for 13 days shows a substantial reduction in motility for treated samples related to untreated controls (left). Absorbance-based quantification of biofilm formation (via measuring the OD_{562}) shows that treatment of *H. pylori* with FucSBn **6** led to a significant reduction in biofilm formation relative to untreated controls (-inhib.). Asterisks indicate a statistically significant difference between mean fluorescence intensity of that sample relative to the negative control sample determined by ANOVA and Tukey's test ($p < 0.05$).

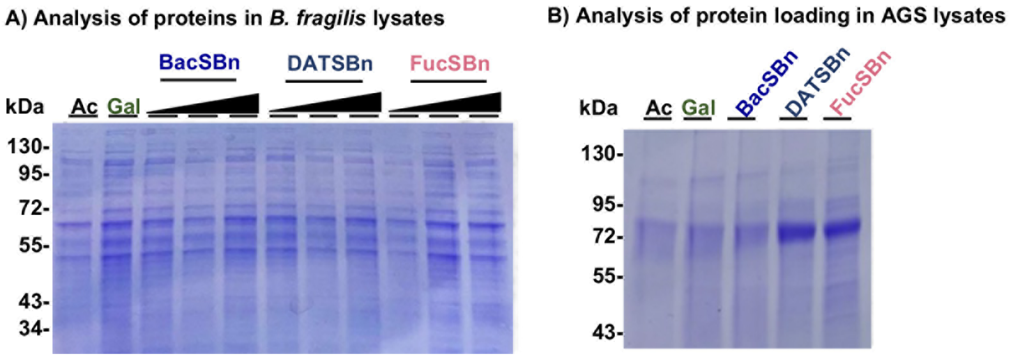

Figure S2. Protein loading for samples presented in Figure 4A and C, respectively. A) Coomassie staining of electrophoresed samples from Figure 4A revealed that Western samples contained roughly equivalent protein levels. B) Coomassie staining of electrophoresed samples from Figure 4C revealed that western samples contained roughly equivalent protein levels.

<u>Supplemental References</u>

1. Baltrus, D. A.; Amieva, M. R.; Covacci, A.; Lowe, T. M.; Merrell, D. S.; Ottemann, K. M.; Stein, M.; Salama, N. R.; Guillemin, K., *The complete genome sequence of Helicobacter pylori* strain G27. *J. Bacteriol.* **2009,** *191* (1), 447-448.
2. O'Toole, G. A., Microtiter dish biofilm formation assay. *Journal of Visualized Experiments* **2011,** (47), e2437.

SSK-23-AP-1260-1H

Current Data Parameters
NAME SSK-23-AP-1260-1H
EXPNO 1
PROCNO 1

F2 - Acquisition Parameters
Date_ 20230329
Time 20.41 h
INSTRUM Avance
PROBHD Z163739_0237 (
PULPROG zg30
TD 51724
SOLVENT CDCl3
NS 18
DS 0
SWH 8620.689 Hz
FIDRES 0.333334 Hz
AQ 2.9999919 sec
RG 52
DW 58.000 usec
DE 13.14 usec
TE 297.5 K
D1 1.00000000 sec
TD0 1
SFO1 400.3024719 MHz
NUC1 1H
P0 2.67 usec
P1 8.00 usec
PLW1 21.00099945 W

F2 - Processing parameters
SI 65536
SF 400.3000000 MHz
WDW EM
SSB 0
LB 0.30 Hz
GB 0
PC 1.00

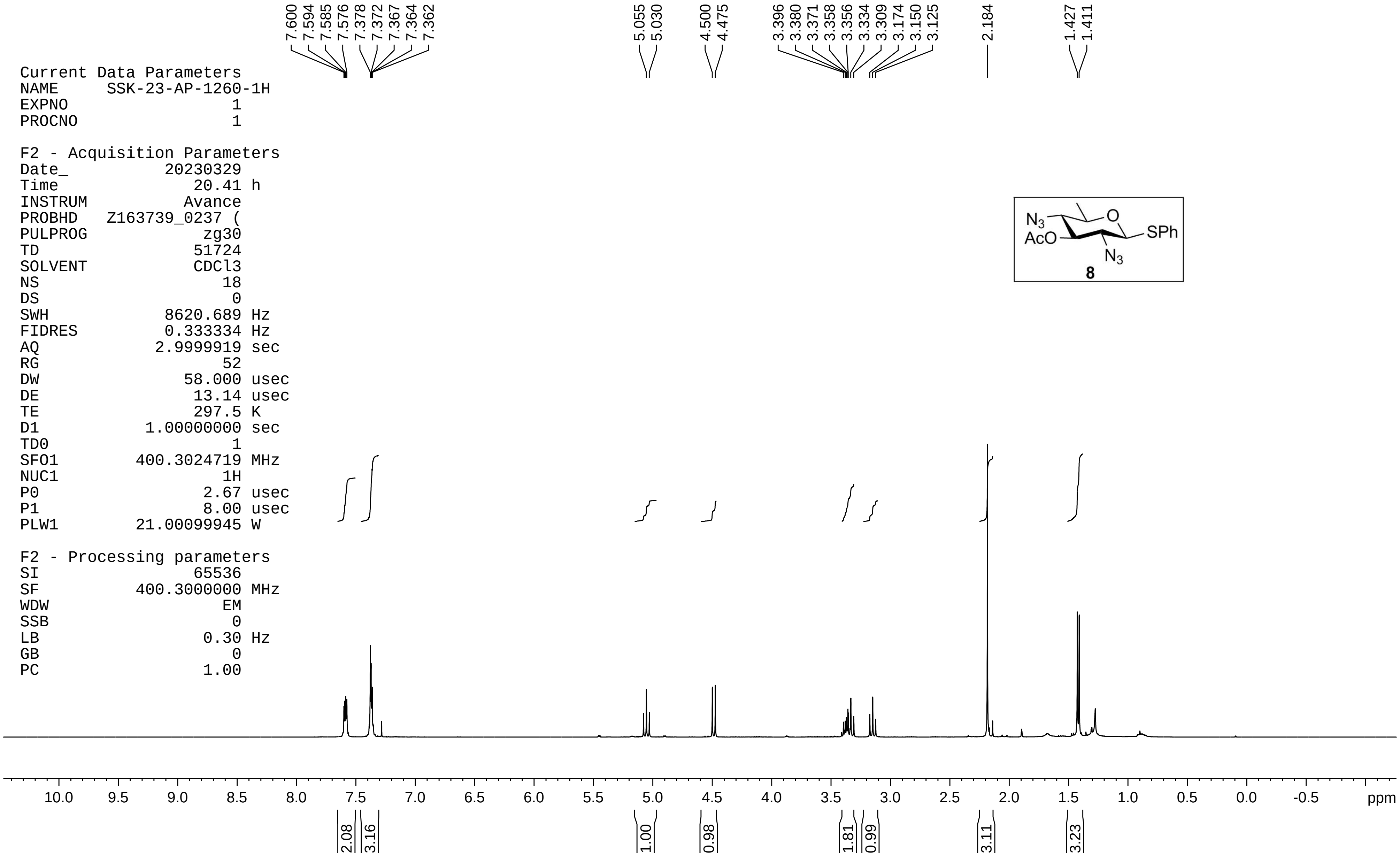

SSK-23-AP-1260-13C

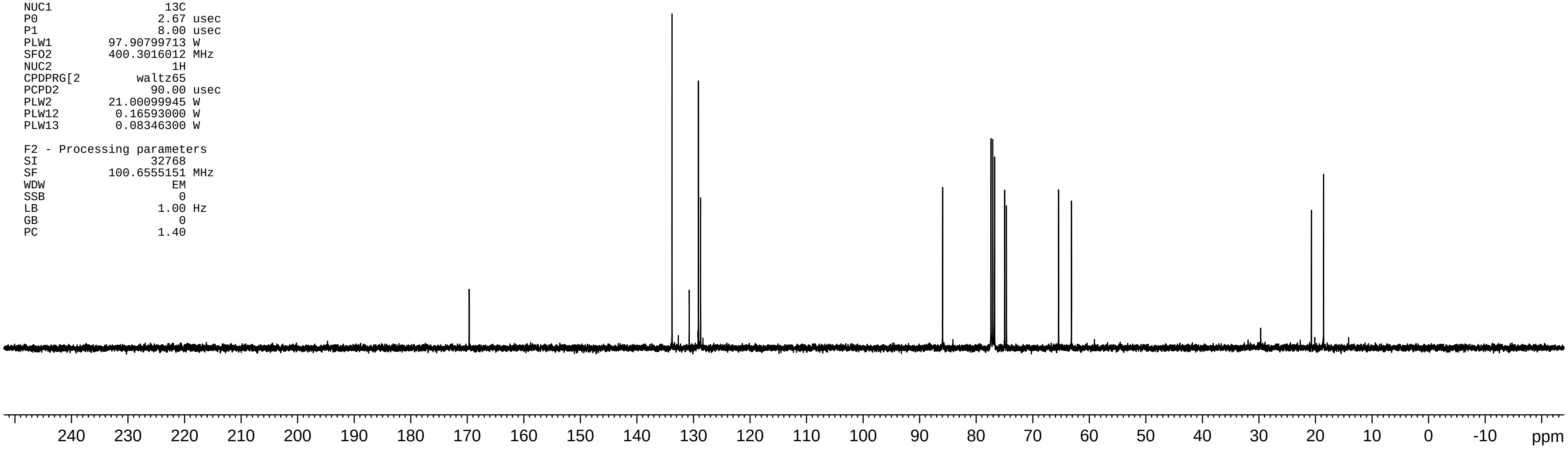

Current Data Parameters
NAME SSK-23-AP-1260-13C
EXPNO 3
PROCNO 1

F2 - Acquisition Parameters
Date_ 20230329
Time 20.44 h
INSTRUM Avance
PROBHD Z163739_0237 (
PULPROG zgpg30
TD 65536
SOLVENT CDCl3
NS 70
DS 0
SWH 27777.777 Hz
FIDRES 0.847710 Hz
AQ 1.1796480 sec
RG 101
DW 18.000 usec
DE 6.50 usec
TE 298.0 K
D1 1.00000000 sec
D11 0.03000000 sec
TD0 1
SFO1 100.6669898 MHz
NUC1 13C
P0 2.67 usec
P1 8.00 usec
PLW1 97.90799713 W
SFO2 400.3016012 MHz
NUC2 1H
CPDPRG[2 waltz65
PCPD2 90.00 usec
PLW2 21.00099945 W
PLW12 0.16593000 W
PLW13 0.08346300 W

F2 - Processing parameters
SI 32768
SF 100.6555151 MHz
WDW EM
SSB 0
LB 1.00 Hz
GB 0
PC 1.40

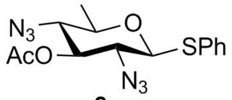

8

SSK-23-AP-1054-1H

```
Current Data Parameters
NAME     SSK-23-AP-1054-1H
EXPNO                    1
PROCNO                   1

F2 - Acquisition Parameters
Date_           20220419
Time               14.27 h
INSTRUM           Avance
PROBHD   Z824601_0063 (
PULPROG             zg30
TD                 51724
SOLVENT            CDCl3
NS                    14
DS                     0
SWH             8620.689 Hz
FIDRES          0.333334 Hz
AQ             2.9999919 sec
RG               54.7368
DW                58.000 usec
DE                 11.94 usec
TE                 297.2 K
D1            1.00000000 sec
TD0                    1
SFO1         400.3024719 MHz
NUC1                  1H
P0                  5.00 usec
P1                 15.00 usec
PLW1          9.20670033 W

F2 - Processing parameters
SI                 65536
SF           400.3000000 MHz
WDW                   EM
SSB                    0
LB                  0.30 Hz
GB                     0
PC                  1.00
```

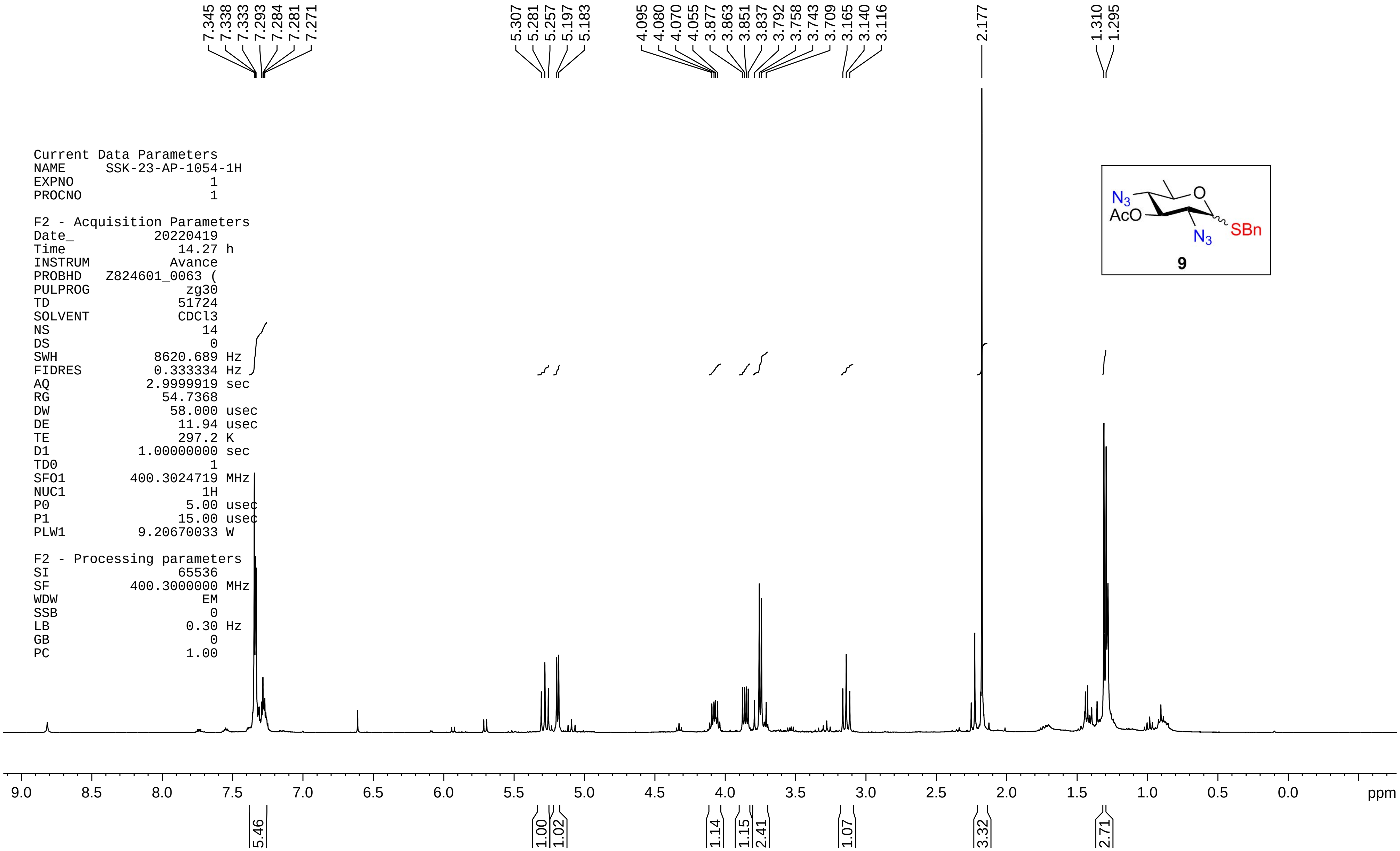

9

SSK-23-AP-1054-13C

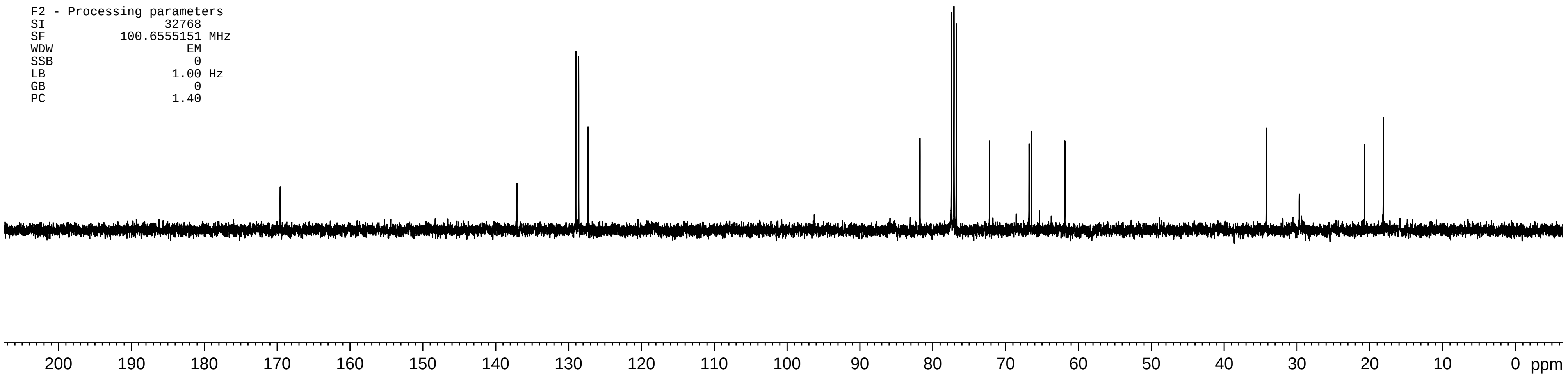

Current Data Parameters

NAME	SSK-23-AP-1054-13C
EXPNO	6
PROCNO	1

F2 - Acquisition Parameters

Date_	20220419
Time	14.34 h
INSTRUM	Avance
PROBHD	Z824601_0063 (
PULPROG	zgpg30
TD	65536
SOLVENT	CDCl3
NS	11
DS	0
SWH	27777.777 Hz
FIDRES	0.847710 Hz
AQ	1.1796480 sec
RG	8.125
DW	18.000 usec
DE	6.50 usec
TE	297.2 K
D1	1.00000000 sec
D11	0.03000000 sec
TD0	1
SFO1	100.6669898 MHz
NUC1	13C
P0	3.33 usec
P1	10.00 usec
PLW1	43.41400146 W
SFO2	400.3016012 MHz
NUC2	1H
CPDPRG[2	waltz65
PCPD2	90.00 usec
PLW2	9.20670033 W
PLW12	0.25573999 W
PLW13	0.12864000 W

F2 - Processing parameters

SI	32768
SF	100.6555151 MHz
WDW	EM
SSB	0
LB	1.00 Hz
GB	0
PC	1.40

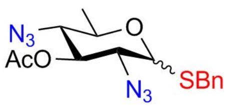

9

SSK-23-AP-1054-DEPT

```
Current Data Parameters
NAME      SSK-23-AP-1054-DEPT
EXPNO                     8
PROCNO                    1

F2 - Acquisition Parameters
Date_            20220419
Time                14.35 h
INSTRUM            Avance
PROBHD   Z824601_0063 (
PULPROG        deptsp135
TD                  65536
SOLVENT             CDCl3
NS                     10
DS                      0
SWH             27777.777 Hz
FIDRES           0.847710 Hz
AQ              1.1796480 sec
RG                    101
DW                 18.000 usec
DE                   6.50 usec
TE                  297.4 K
CNST2         145.0000000
D1             1.00000000 sec
D2             0.00344828 sec
D12            0.00002000 sec
TD0                     1
SFO1          100.6669898 MHz
NUC1                  13C
P1                  10.00 usec
P13               2000.00 usec
PLW0                    0 W
PLW1          43.41400146 W
SPNAM[5]     Crp60comp.4
SPOAL5              0.500
SPOFFS5                 0 Hz
SPW5           6.63320017 W
SFO2          400.3016012 MHz
NUC2                   1H
CPDPRG[2           waltz65
P3                  15.00 usec
P4                  30.00 usec
PCPD2               90.00 usec
PLW2           9.20670033 W
PLW12          0.25573999 W

F2 - Processing parameters
SI                  32768
SF            100.6555151 MHz
WDW                    EM
SSB                     0
LB                   1.00 Hz
GB                      0
PC                   1.40
```

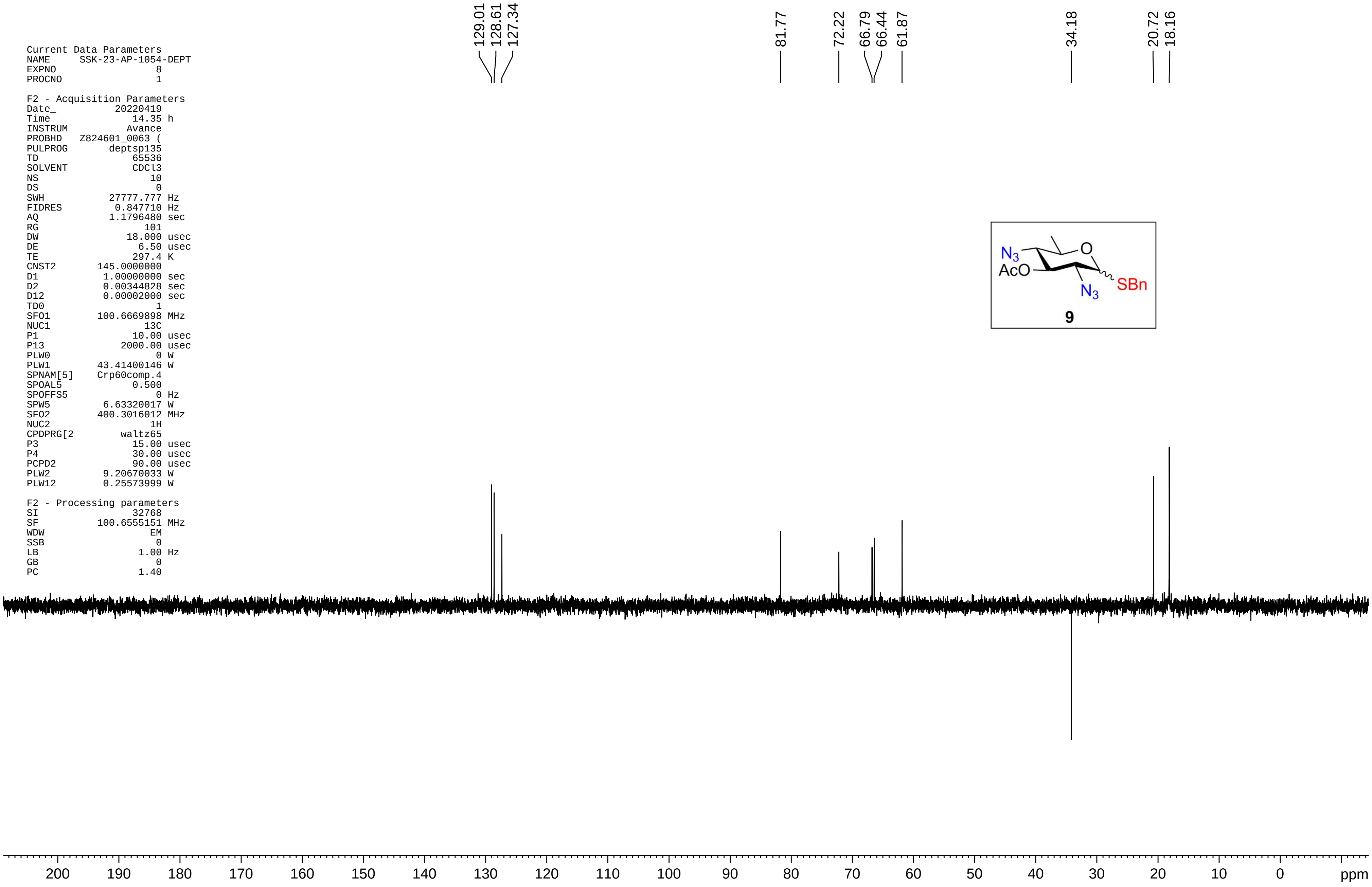

9

SSK-23-AP-1054-COSY

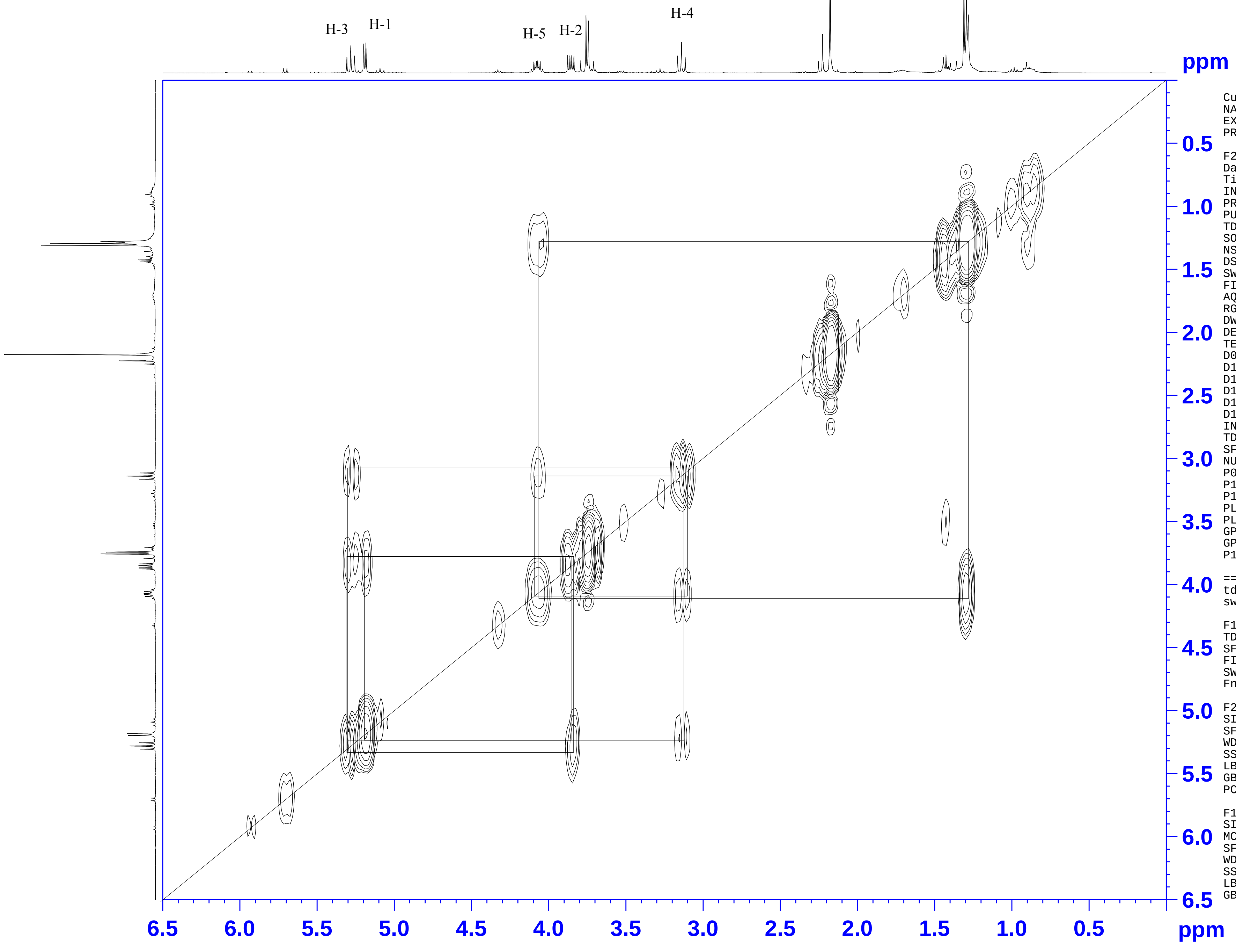

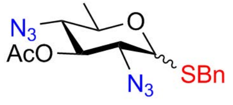

9

```
Current Data Parameters
NAME      SSK-23-AP-1054-COSY
EXPNO                       3
PROCNO                      1

F2 - Acquisition Parameters
Date_              20220419
Time                  14.33 h
INSTRUM              Avance
PROBHD    Z824601_0063 (
PULPROG          cosygpppqf
TD                     2048
SOLVENT               CDCl3
NS                        4
DS                        0
SWH                8620.689 Hz
FIDRES             8.418642 Hz
AQ                0.1187840 sec
RG                      101
DW                   58.000 usec
DE                     6.50 usec
TE                    297.2 K
D0               0.00000300 sec
D1               1.00000000 sec
D11              0.03000000 sec
D12              0.00002000 sec
D13              0.00000400 sec
D16              0.00020000 sec
IN0              0.00011355 sec
TDav                      1
SFO1            400.3024018 MHz
NUC1                     1H
P0                    15.00 usec
P1                    15.00 usec
P17                 2500.00 usec
PLW1             9.20670033 W
PLW10            2.30170012 W
GPNAM[1]        SMSQ10.100
GPZ1                  10.00 %
P16                 1000.00 usec

======== F1 INDIRECT DIMENSION ========
td1        128
sw_F1      22.000000

F1 - Acquisition parameters
TD                       67
SFO1               400.3024 MHz
FIDRES           262.886353 Hz
SW                   22.000 ppm
FnMODE                   QF

F2 - Processing parameters
SI                     1024
SF              400.3000000 MHz
WDW                   QSINE
SSB                       0
LB                        0 Hz
GB                        0
PC                     1.40

F1 - Processing parameters
SI                     1024
MC2                      QF
SF              400.3000000 MHz
WDW                   QSINE
SSB                       0
LB                        0 Hz
GB                        0
```

SSK-23-AP-1056-1H

Current Data Parameters
NAME SSK-23-AP-1056-1H
EXPNO 10
PROCNO 1

F2 - Acquisition Parameters
Date_ 20220420
Time 20.22 h
INSTRUM Avance Neo 400
PROBHD Z163739_0226 (
PULPROG zg30
TD 51724
SOLVENT CDCl3
NS 7
DS 0
SWH 8620.689 Hz
FIDRES 0.333334 Hz
AQ 2.9999919 sec
RG 32
DW 58.000 usec
DE 13.14 usec
TE 298.9 K
D1 1.00000000 sec
TD0 1
SFO1 400.1324708 MHz
NUC1 1H
P0 2.67 usec
P1 8.00 usec
PLW1 25.07999992 W

F2 - Processing parameters
SI 65536
SF 400.1300000 MHz
WDW EM
SSB 0
LB 0.30 Hz
GB 0
PC 1.00

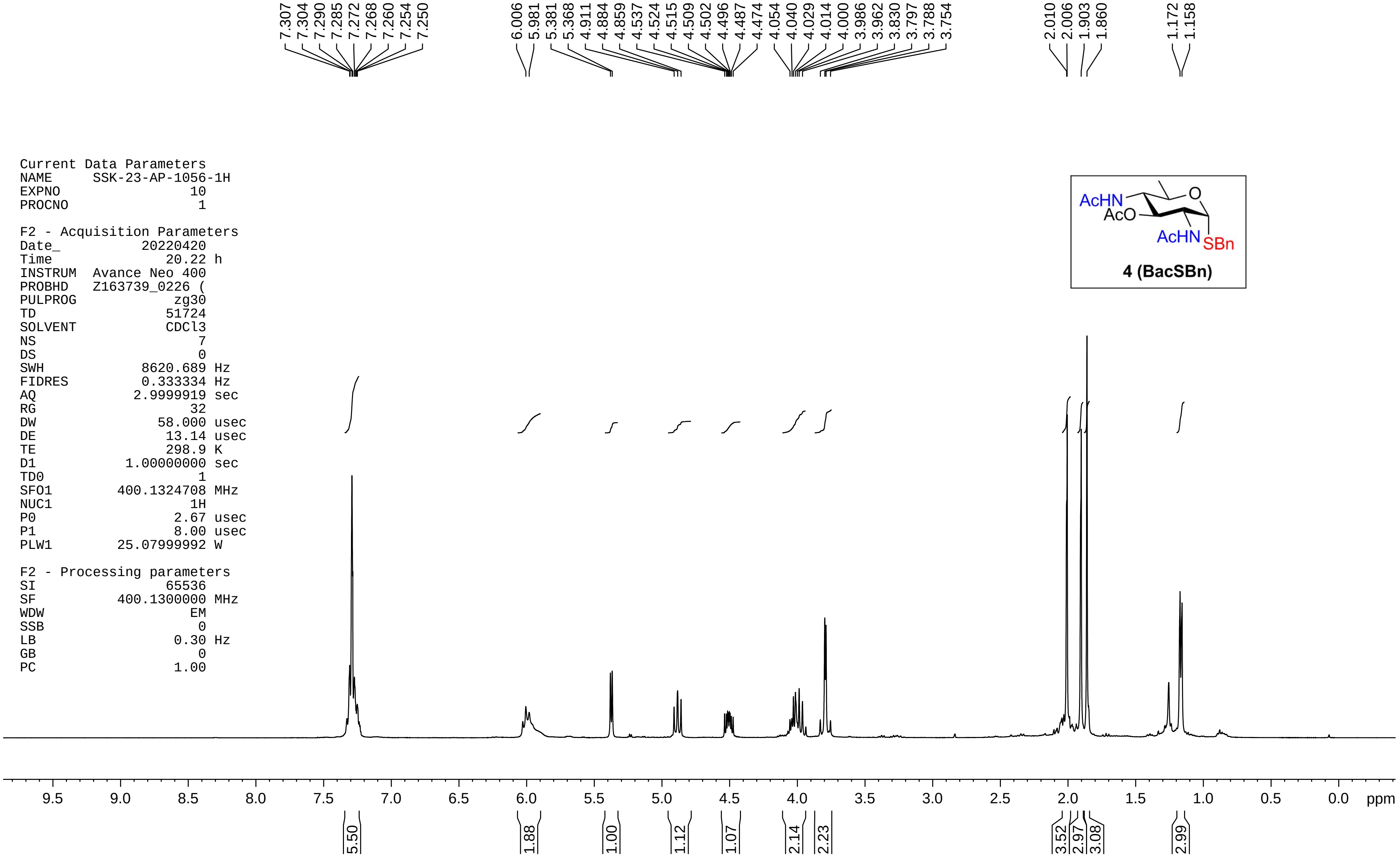

4 (BacSBn)

SSK-23-AP-1056-13C

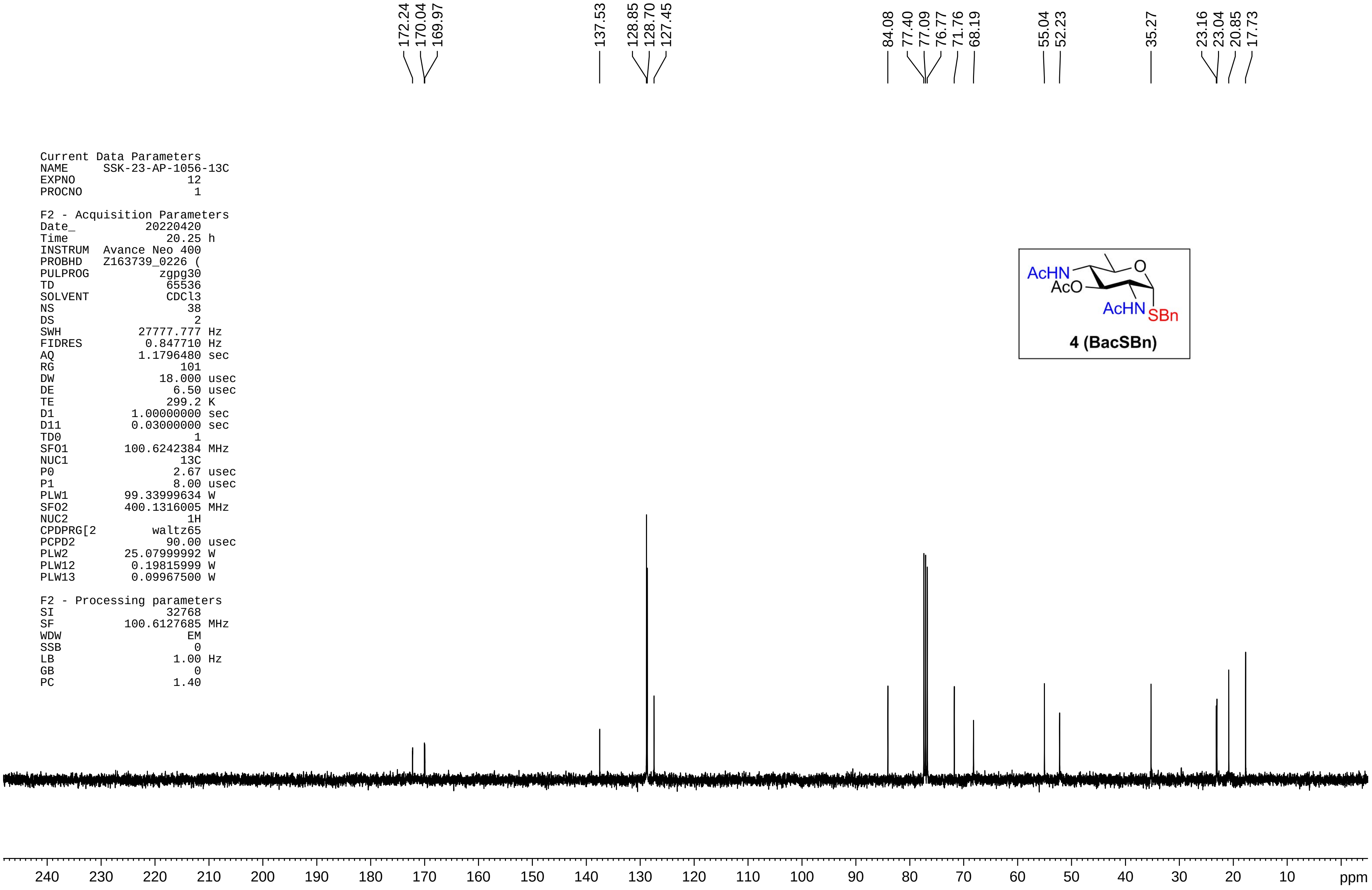


```
Current Data Parameters
NAME      SSK-23-AP-1056-13C
EXPNO                    12
PROCNO                    1

F2 - Acquisition Parameters
Date_           20220420
Time               20.25 h
INSTRUM   Avance Neo 400
PROBHD    Z163739_0226 (
PULPROG           zgpg30
TD                 65536
SOLVENT            CDCl3
NS                    38
DS                     2
SWH            27777.777 Hz
FIDRES          0.847710 Hz
AQ             1.1796480 sec
RG                   101
DW                18.000 usec
DE                  6.50 usec
TE                 299.2 K
D1            1.00000000 sec
D11           0.03000000 sec
TD0                    1
SFO1        100.6242384 MHz
NUC1                 13C
P0                  2.67 usec
P1                  8.00 usec
PLW1         99.33999634 W
SFO2         400.1316005 MHz
NUC2                  1H
CPDPRG[2         waltz65
PCPD2              90.00 usec
PLW2         25.07999992 W
PLW12         0.19815999 W
PLW13         0.09967500 W

F2 - Processing parameters
SI                 32768
SF           100.6127685 MHz
WDW                   EM
SSB                    0
LB                  1.00 Hz
GB                     0
PC                  1.40
```


4 (BacSBn)

SSK-24-AP-1056-DEPT

```
Current Data Parameters
NAME     SSK-24-AP-1056-DEPT
EXPNO                     1
PROCNO                    1

F2 - Acquisition Parameters
Date_          20220421
Time              17.11 h
INSTRUM          Avance
PROBHD   Z824601_0063 (
PULPROG       deptsp135
TD                65536
SOLVENT           CDCl3
NS                   50
DS                    0
SWH           27777.777 Hz
FIDRES         0.847710 Hz
AQ            1.1796480 sec
RG                  101
DW               18.000 usec
DE                 6.50 usec
TE                297.8 K
CNST2       145.0000000
D1           1.00000000 sec
D2           0.00344828 sec
D12          0.00002000 sec
TD0                   1
SFO1        100.6669898 MHz
NUC1                13C
P1                10.00 usec
P13             2000.00 usec
PLW0                  0 W
PLW1        43.41400146 W
SPNAM[5]    Crp60comp.4
SPOAL5            0.500
SPOFFS5               0 Hz
SPW5         6.63320017 W
SFO2        400.3016012 MHz
NUC2                 1H
CPDPRG[2        waltz65
P3                15.00 usec
P4                30.00 usec
PCPD2             90.00 usec
PLW2         9.20670033 W
PLW12        0.25573999 W

F2 - Processing parameters
SI                32768
SF          100.6555151 MHz
WDW                  EM
SSB                   0
LB                 1.00 Hz
GB                    0
PC                 1.40
```

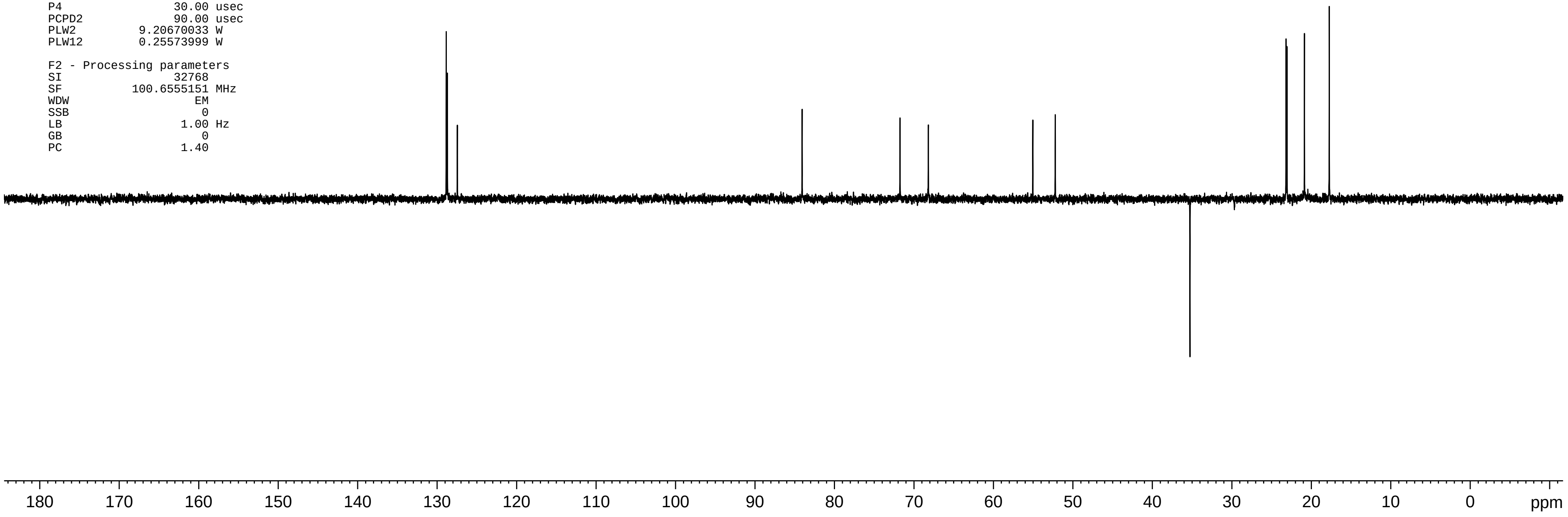

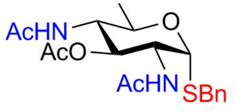

4 (BacSBn)

SSK-24-AP-1056-COSY

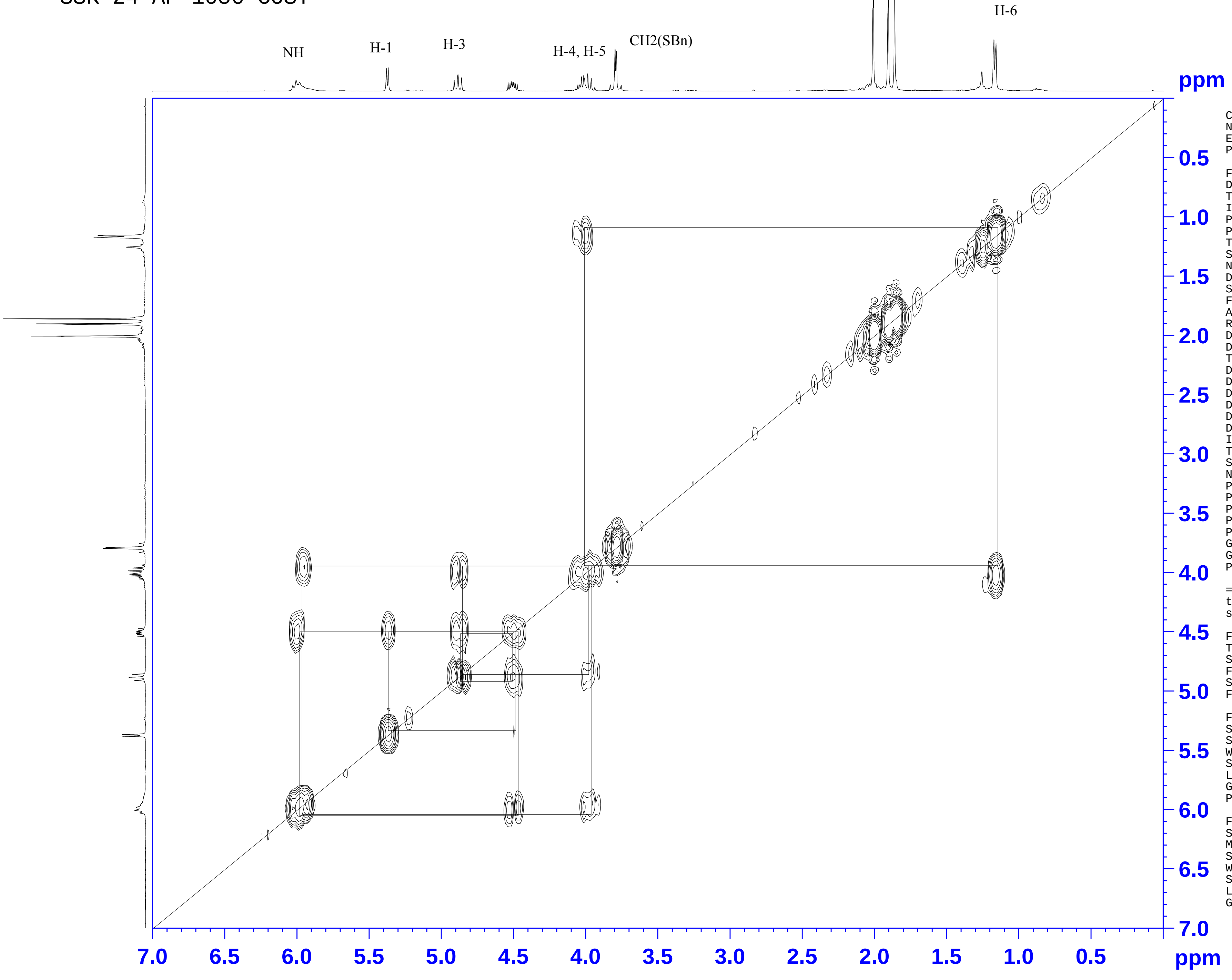

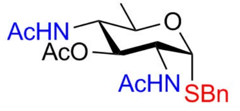

4 (BacSBn)

```
Current Data Parameters
NAME       SSK-24-AP-1056-COSY
EXPNO                      3
PROCNO                     1

F2 - Acquisition Parameters
Date_              20220421
Time                  17.21 h
INSTRUM              Avance
PROBHD    Z824601_0063 (
PULPROG         cosygpppqf
TD                     2048
SOLVENT               CDCl3
NS                        4
DS                        0
SWH                8620.689 Hz
FIDRES             8.418642 Hz
AQ                0.1187840 sec
RG                      101
DW                   58.000 usec
DE                     6.50 usec
TE                    297.6 K
D0               0.00000300 sec
D1               1.00000000 sec
D11              0.03000000 sec
D12              0.00002000 sec
D13              0.00000400 sec
D16              0.00020000 sec
IN0              0.00011355 sec
TDav                      1
SFO1            400.3024018 MHz
NUC1                     1H
P0                    15.00 usec
P1                    15.00 usec
P17                 2500.00 usec
PLW1             9.20670033 W
PLW10            2.30170012 W
GPNAM[1]         SMSQ10.100
GPZ1                  10.00 %
P16                 1000.00 usec

======== F1 INDIRECT DIMENSION ========
td1       128
sw_F1     22.000000

F1 - Acquisition parameters
TD                      128
SFO1               400.3024 MHz
FIDRES           137.604584 Hz
SW                   22.000 ppm
FnMODE                   QF

F2 - Processing parameters
SI                     1024
SF              400.3000000 MHz
WDW                   QSINE
SSB                       0
LB                        0 Hz
GB                        0
PC                     1.40

F1 - Processing parameters
SI                     1024
MC2                      QF
SF              400.3000000 MHz
WDW                   QSINE
SSB                       0
LB                        0 Hz
GB                        0
```

SSK-23-AP-DAT-1H

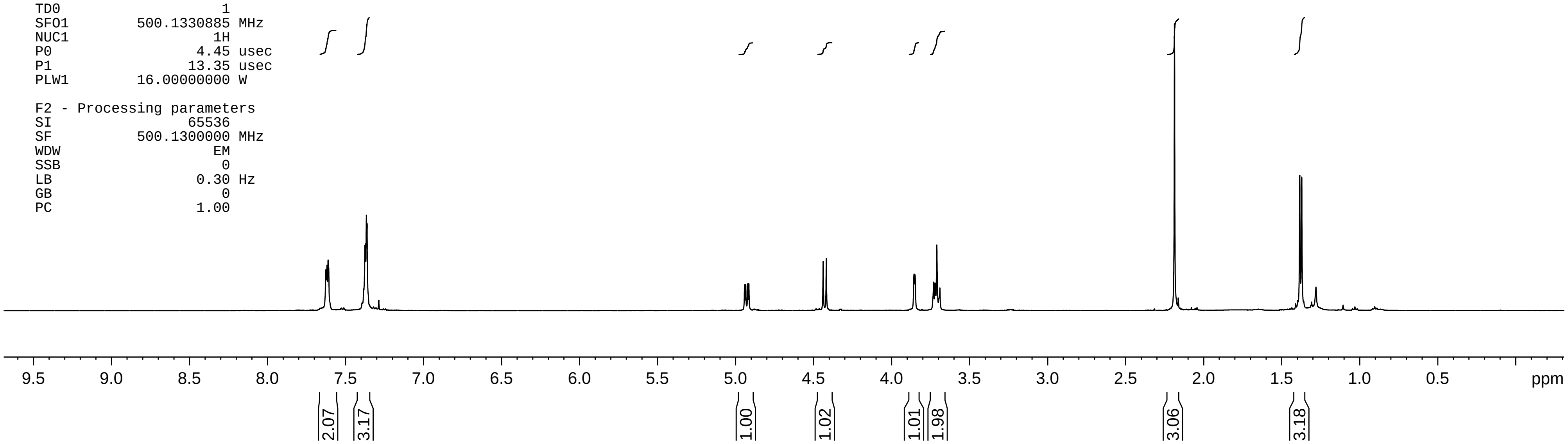


```
Current Data Parameters
NAME     SSK-23-AP-DAT-1H
EXPNO                  16
PROCNO                  1

F2 - Acquisition Parameters
Date_          20230607
Time              16.24 h
INSTRUM           spect
PROBHD   Z119470_0087 (
PULPROG            zg30
TD                65536
SOLVENT           CDCl3
NS                   13
DS                    0
SWH          10000.000 Hz
FIDRES        0.305176 Hz
AQ           3.2767999 sec
RG               48.36
DW              50.000 usec
DE                6.50 usec
TE               298.0 K
D1          1.00000000 sec
TD0                   1
SFO1       500.1330885 MHz
NUC1                 1H
P0                 4.45 usec
P1                13.35 usec
PLW1       16.00000000 W

F2 - Processing parameters
SI                65536
SF         500.1300000 MHz
WDW                  EM
SSB                   0
LB                 0.30 Hz
GB                    0
PC                 1.00
```

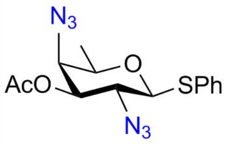

10

SSK-23-AP-DAT-13C

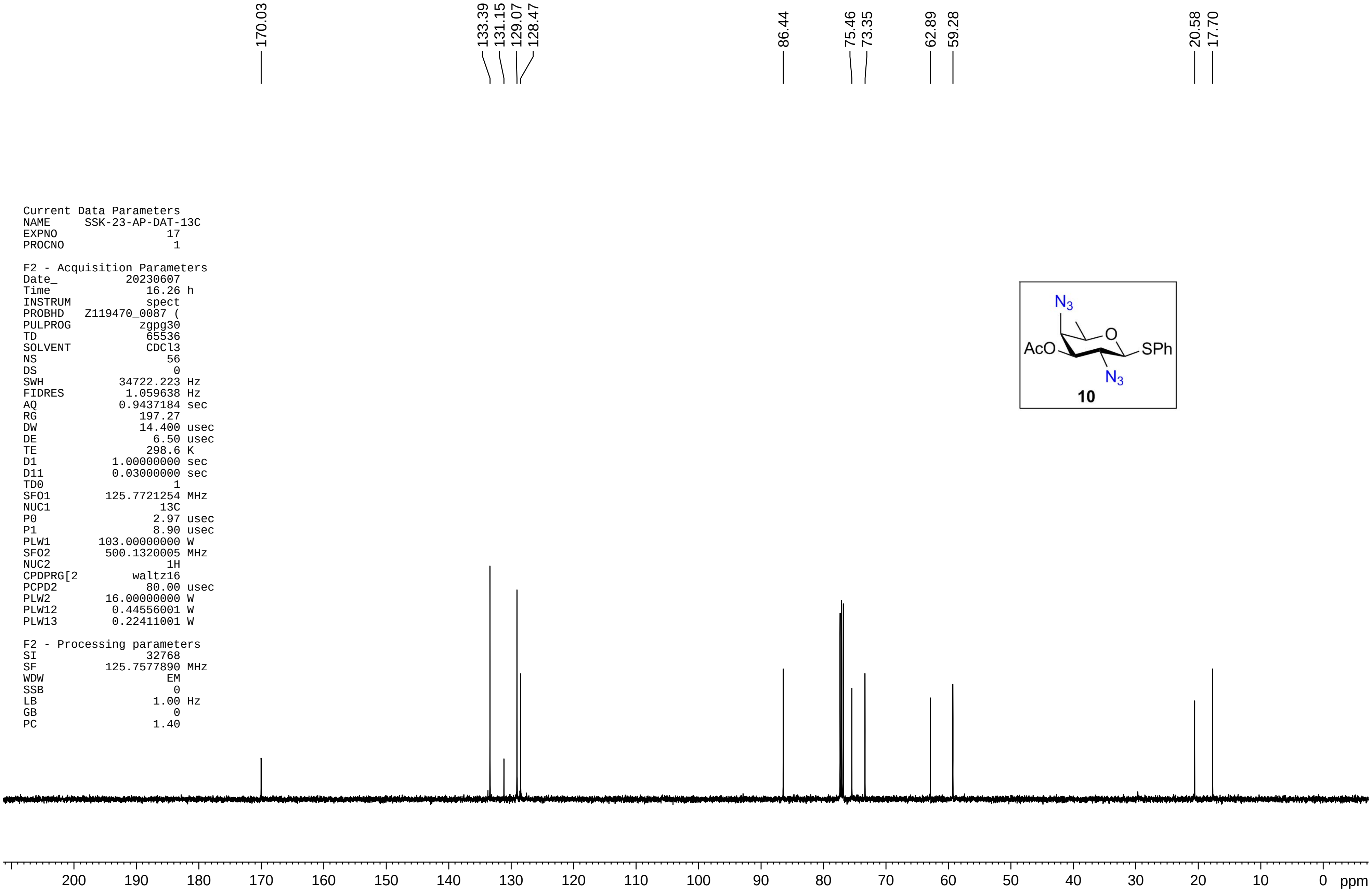


```
Current Data Parameters
NAME     SSK-23-AP-DAT-13C
EXPNO                   17
PROCNO                   1

F2 - Acquisition Parameters
Date_           20230607
Time               16.26 h
INSTRUM            spect
PROBHD   Z119470_0087 (
PULPROG          zgpg30
TD                 65536
SOLVENT            CDCl3
NS                    56
DS                     0
SWH            34722.223 Hz
FIDRES          1.059638 Hz
AQ             0.9437184 sec
RG                197.27
DW                14.400 usec
DE                  6.50 usec
TE                 298.6 K
D1            1.00000000 sec
D11           0.03000000 sec
TD0                    1
SFO1         125.7721254 MHz
NUC1                 13C
P0                  2.97 usec
P1                  8.90 usec
PLW1        103.00000000 W
SFO2         500.1320005 MHz
NUC2                  1H
CPDPRG[2         waltz16
PCPD2              80.00 usec
PLW2         16.00000000 W
PLW12         0.44556001 W
PLW13         0.22411001 W

F2 - Processing parameters
SI                 32768
SF           125.7577890 MHz
WDW                   EM
SSB                    0
LB                  1.00 Hz
GB                     0
PC                  1.40
```


10

SSK-23-AP-1215-1H

Current Data Parameters

NAME	SSK-23-AP-1215-1H
EXPNO	10
PROCNO	1

F2 - Acquisition Parameters

Date_	20230212
Time	15.06 h
INSTRUM	Avance Neo 400
PROBHD	Z163739_0226 (
PULPROG	zg30
TD	51724
SOLVENT	CDCl3
NS	10
DS	0
SWH	8620.689 Hz
FIDRES	0.333334 Hz
AQ	2.9999919 sec
RG	32
DW	58.000 usec
DE	13.14 usec
TE	295.4 K
D1	1.00000000 sec
TD0	1
SFO1	400.1324708 MHz
NUC1	1H
P0	2.67 usec
P1	8.00 usec
PLW1	25.07999992 W

F2 - Processing parameters

SI	65536
SF	400.1300000 MHz
WDW	EM
SSB	0
LB	0.30 Hz
GB	0
PC	1.00

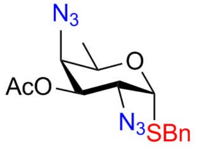

11

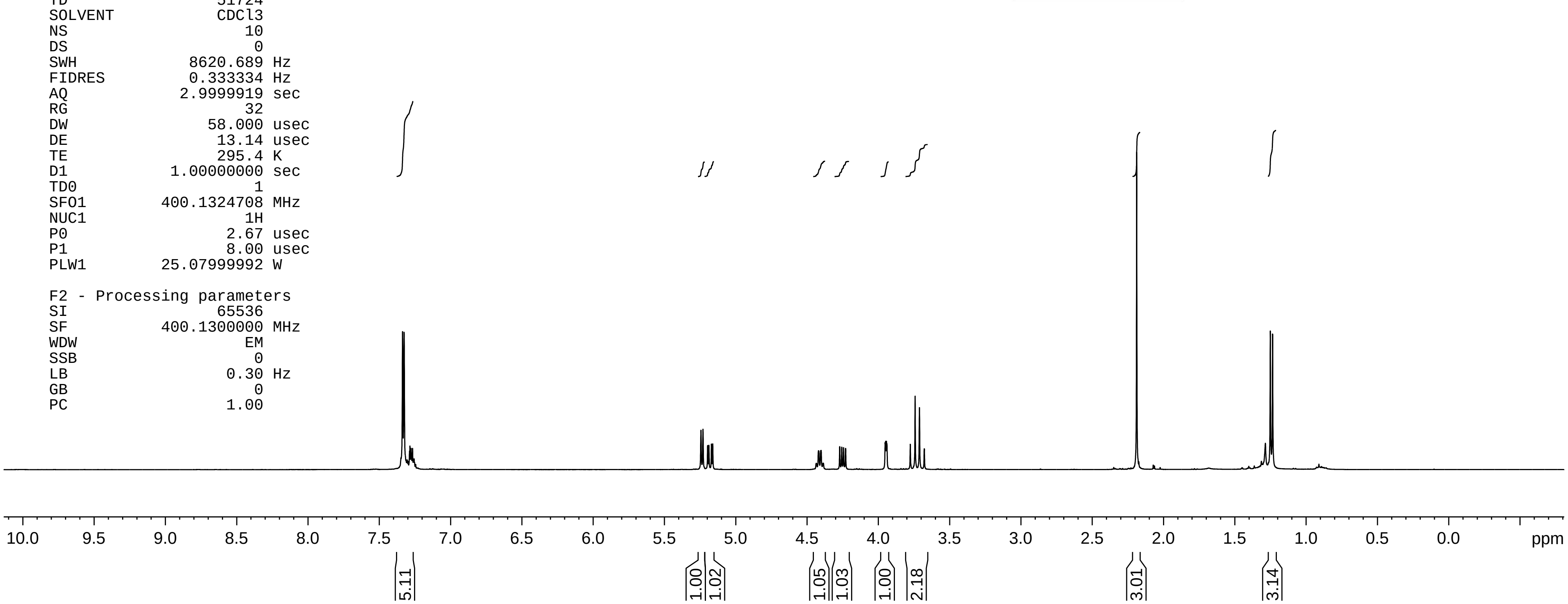

SSK-23-AP-1215-13C

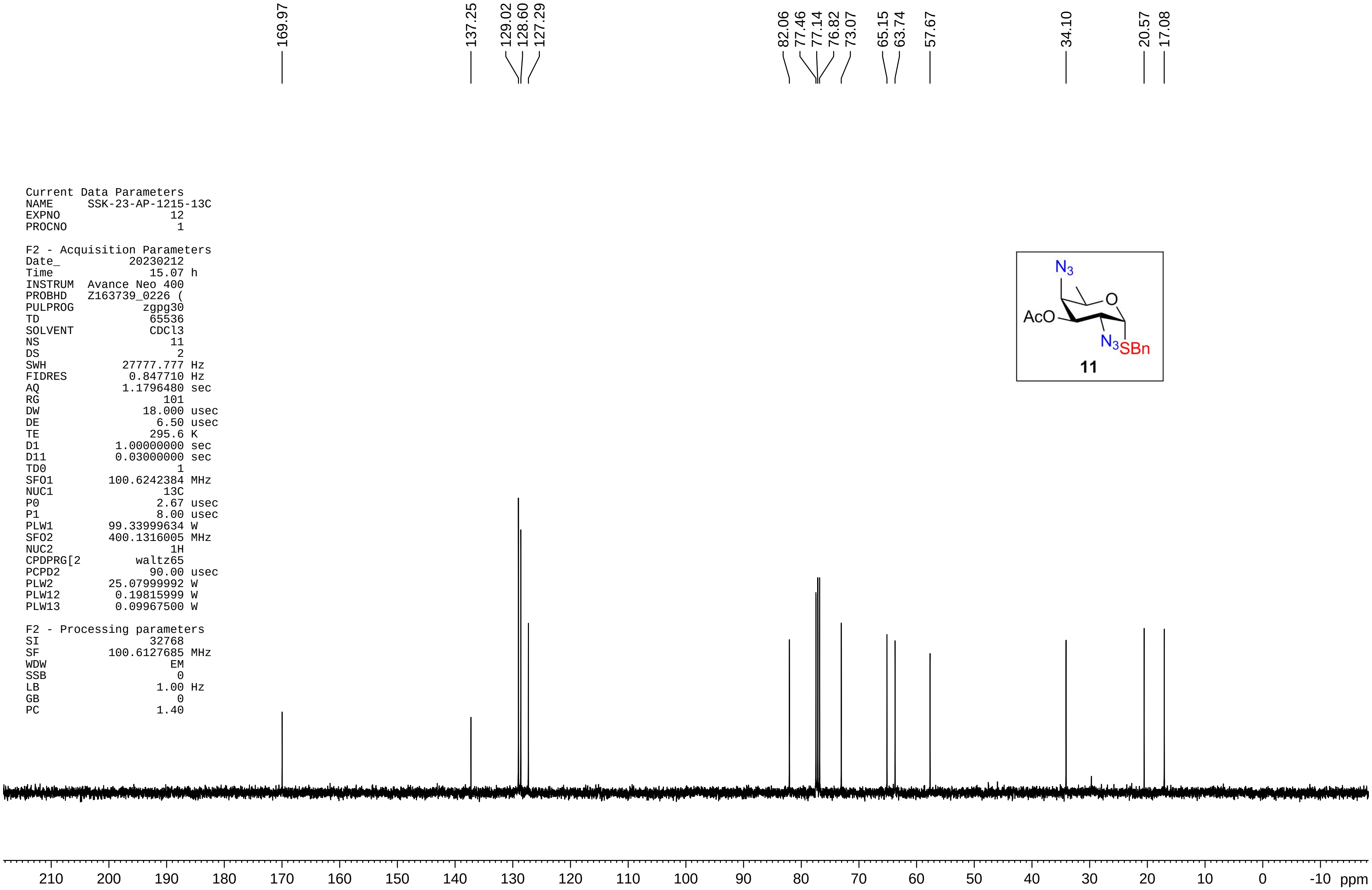


```
Current Data Parameters
NAME      SSK-23-AP-1215-13C
EXPNO                   12
PROCNO                   1

F2 - Acquisition Parameters
Date_           20230212
Time               15.07 h
INSTRUM   Avance Neo 400
PROBHD    Z163739_0226 (
PULPROG           zgpg30
TD                 65536
SOLVENT            CDCl3
NS                    11
DS                     2
SWH            27777.777 Hz
FIDRES          0.847710 Hz
AQ             1.1796480 sec
RG                   101
DW                18.000 usec
DE                  6.50 usec
TE                 295.6 K
D1            1.00000000 sec
D11           0.03000000 sec
TD0                    1
SFO1         100.6242384 MHz
NUC1                 13C
P0                  2.67 usec
P1                  8.00 usec
PLW1         99.33999634 W
SFO2         400.1316005 MHz
NUC2                  1H
CPDPRG[2         waltz65
PCPD2              90.00 usec
PLW2         25.07999992 W
PLW12         0.19815999 W
PLW13         0.09967500 W

F2 - Processing parameters
SI                 32768
SF           100.6127685 MHz
WDW                   EM
SSB                    0
LB                  1.00 Hz
GB                     0
PC                  1.40
```


11

SSK-23-AP-1215-DEPT

```
Current Data Parameters
NAME      SSK-23-AP-1215-DEPT
EXPNO                      14
PROCNO                      1

F2 - Acquisition Parameters
Date_              20230212
Time                  16.45 h
INSTRUM     Avance Neo 400
PROBHD    Z163739_0226 (
PULPROG          deptsp135
TD                    65536
SOLVENT               CDCl3
NS                       16
DS                        4
SWH               27777.777 Hz
FIDRES             0.847710 Hz
AQ                1.1796480 sec
RG                      101
DW                   18.000 usec
DE                     6.50 usec
TE                    295.6 K
CNST2           145.0000000
D1               1.00000000 sec
D2               0.00344828 sec
D12              0.00002000 sec
TD0                       1
SFO1           100.6242384 MHz
NUC1                    13C
P1                     8.00 usec
P13                 2000.00 usec
PLW0                      0 W
PLW1            99.33999634 W
SPNAM[5]       Crp60comp.4
SPOAL5                0.500
SPOFFS5                   0 Hz
SPW5             9.71399975 W
SFO2           400.1316005 MHz
NUC2                     1H
CPDPRG[2            waltz65
P3                     8.00 usec
P4                    16.00 usec
PCPD2                 90.00 usec
PLW2            25.07999992 W
PLW12            0.19815999 W

F2 - Processing parameters
SI                    32768
SF             100.6127685 MHz
WDW                      EM
SSB                       0
LB                     1.00 Hz
GB                        0
PC                     1.40
```

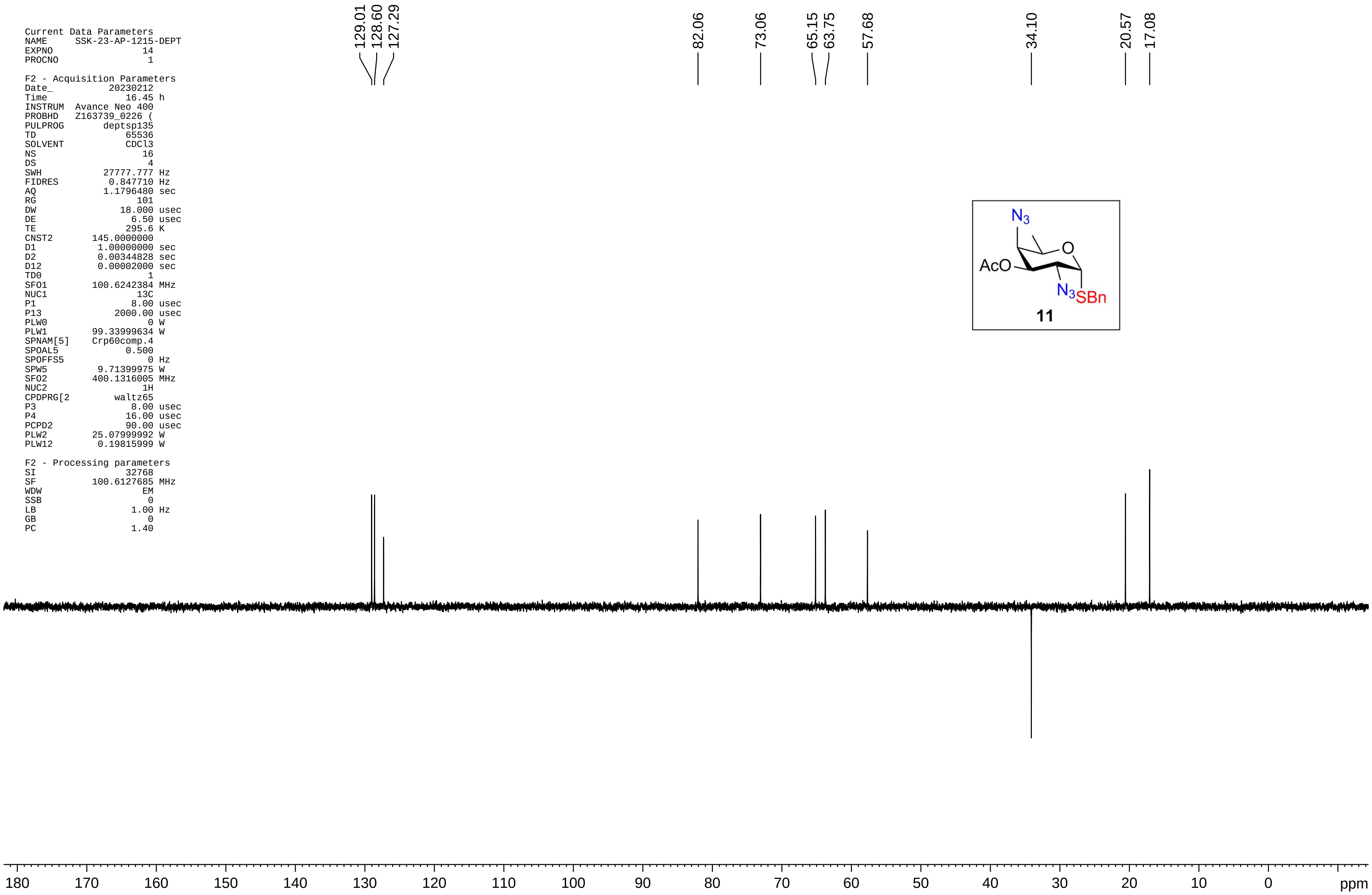

11

SSK-23-AP-1215-COSY

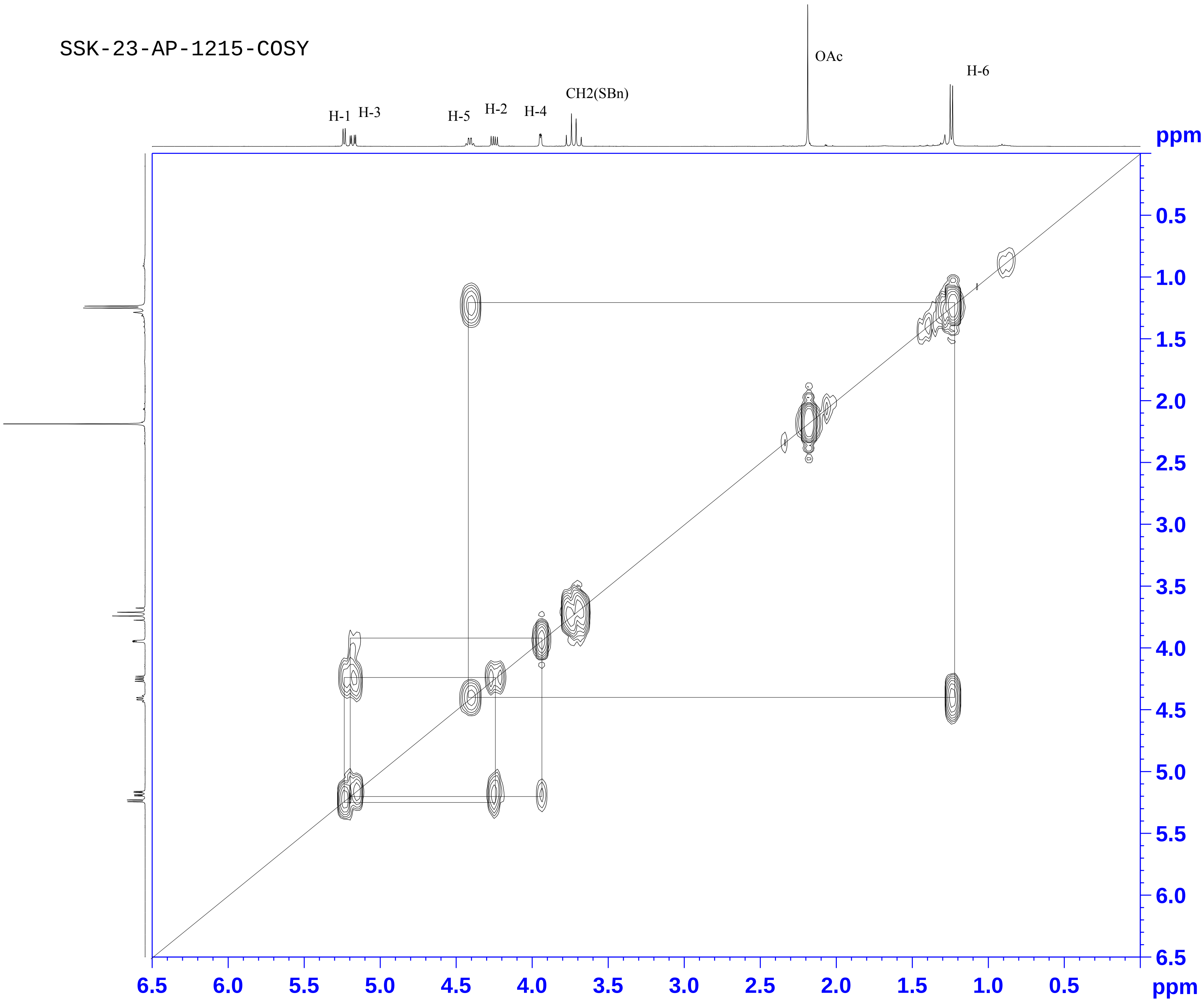

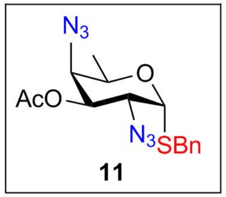


```
Current Data Parameters
NAME        SSK-23-AP-1215-COSY
EXPNO                      10
PROCNO                      1

F2 - Acquisition Parameters
Date_              20230212
Time                  16.20 h
INSTRUM     Avance Neo 400
PROBHD      Z163739_0226 (
PULPROG          cosygpppqf
TD                     2048
SOLVENT               CDCl3
NS                        4
DS                        0
SWH                8620.689 Hz
FIDRES             8.418642 Hz
AQ                0.1187840 sec
RG                       64
DW                   58.000 usec
DE                     6.50 usec
TE                    295.5 K
D0               0.00000300 sec
D1               1.00000000 sec
D11              0.03000000 sec
D12              0.00002000 sec
D13              0.00000400 sec
D16              0.00020000 sec
IN0              0.00011360 sec
TDav                      1
SFO1            400.1324708 MHz
NUC1                     1H
P0                     8.00 usec
P1                     8.00 usec
P17                 2500.00 usec
PLW1            25.07999992 W
PLW10            1.78349996 W
GPNAM[1]         SMSQ10.100
GPZ1                  10.00 %
P16                 1000.00 usec

======== F1 INDIRECT DIMENSION ========
td1         128
sw_F1       21.999996

F1 - Acquisition parameters
TD                      128
SFO1               400.1325 MHz
FIDRES           137.544022 Hz
SW                   22.000 ppm
FnMODE                   QF

F2 - Processing parameters
SI                     1024
SF              400.1300000 MHz
WDW                   QSINE
SSB                       0
LB                        0 Hz
GB                        0
PC                     1.40

F1 - Processing parameters
SI                     1024
MC2                      QF
SF              400.1300000 MHz
WDW                   QSINE
SSB                       0
LB                        0 Hz
GB                        0
```

SSK-23-AP-1215-HSQC

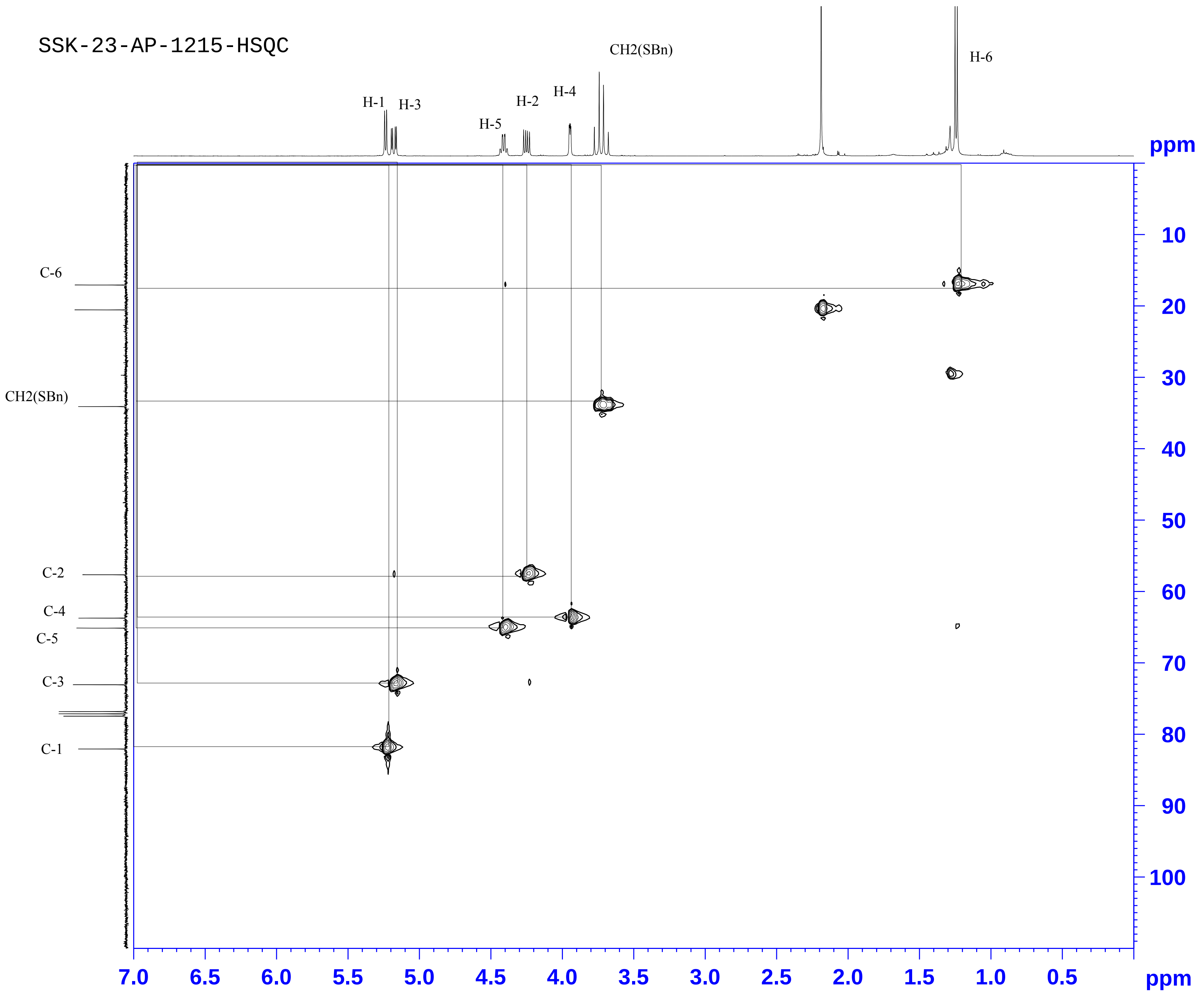

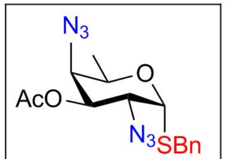

11

```
Current Data Parameters
NAME      SSK-23-AP-1215-HSQC
EXPNO                     12
PROCNO                     1

F2 - Acquisition Parameters
Date_               20230212
Time                   16.40 h
INSTRUM  Avance Neo 400
PROBHD   Z163739_0226 (
PULPROG  hsqcedetgpsisp2.3
TD                      2048
SOLVENT                CDCl3
NS                         4
DS                         0
SWH                 8620.689 Hz
FIDRES              8.418642 Hz
AQ                 0.1187840 sec
RG                       101
DW                    58.000 usec
DE                      6.50 usec
TE                     295.5 K
CNST2          145.0000000
CNST17          -0.5000000
D0              0.00000300 sec
D1              1.00000000 sec
D4              0.00172414 sec
D11             0.03000000 sec
D16             0.00020000 sec
D21             0.00344800 sec
D24             0.00086200 sec
IN0             0.00001801 sec
TDav                       1
ZGOPTNS
SFO1           400.1324708 MHz
NUC1                      1H
P1                      8.00 usec
P2                     16.00 usec
PLW1           25.07999992 W
SFO2           100.6242384 MHz
NUC2                     13C
CPDPRG[2               garp4
P3                      8.00 usec
P14                   500.00 usec
P24                  2000.00 usec
P31                  2119.00 usec
PCPD2                  80.00 usec
PLW0                       0 W
PLW2           99.33999634 W
PLW12           0.99339998 W
SPNAM[3] Crp60,0.5,20.1
SPOAL3                 0.500
SPOFFS3                    0 Hz
SPW3            9.71399975 W
SPNAM[7]     Crp60comp.4
SPOAL7                 0.500
SPOFFS7                    0 Hz
SPW7            9.71399975 W
SPNAM[18   Crp60_xfilt.2
SPOAL18                0.500
SPOFFS18                   0 Hz
SPW18           1.87129998 W
GPNAM[1]      SMSQ10.100
GPZ1                   80.00 %
GPNAM[2]      SMSQ10.100
GPZ2                   20.10 %
GPNAM[3]      SMSQ10.100
GPZ3                   11.00 %
GPNAM[4]      SMSQ10.100
GPZ4                   -5.00 %
P16                  1000.00 usec
P19                   600.00 usec

======== F1 INDIRECT DIMENSION ========
td1      1024
sw_F1    275.989227

F1 - Acquisition parameters
TD                       248
SFO1                100.6242 MHz
FIDRES            223.936584 Hz
SW                   275.959 ppm
FnMODE      Echo-Antiecho

F2 - Processing parameters
SI                      1024
SF               400.1300000 MHz
WDW                    QSINE
SSB                        2
LB                         0 Hz
GB                         0
PC                      1.40

F1 - Processing parameters
SI                      1024
MC2           echo-antiecho
SF               100.6127685 MHz
WDW                    QSINE
SSB                        2
LB                         0 Hz
GB                         0
```

SSK-23-AP-1219-1H

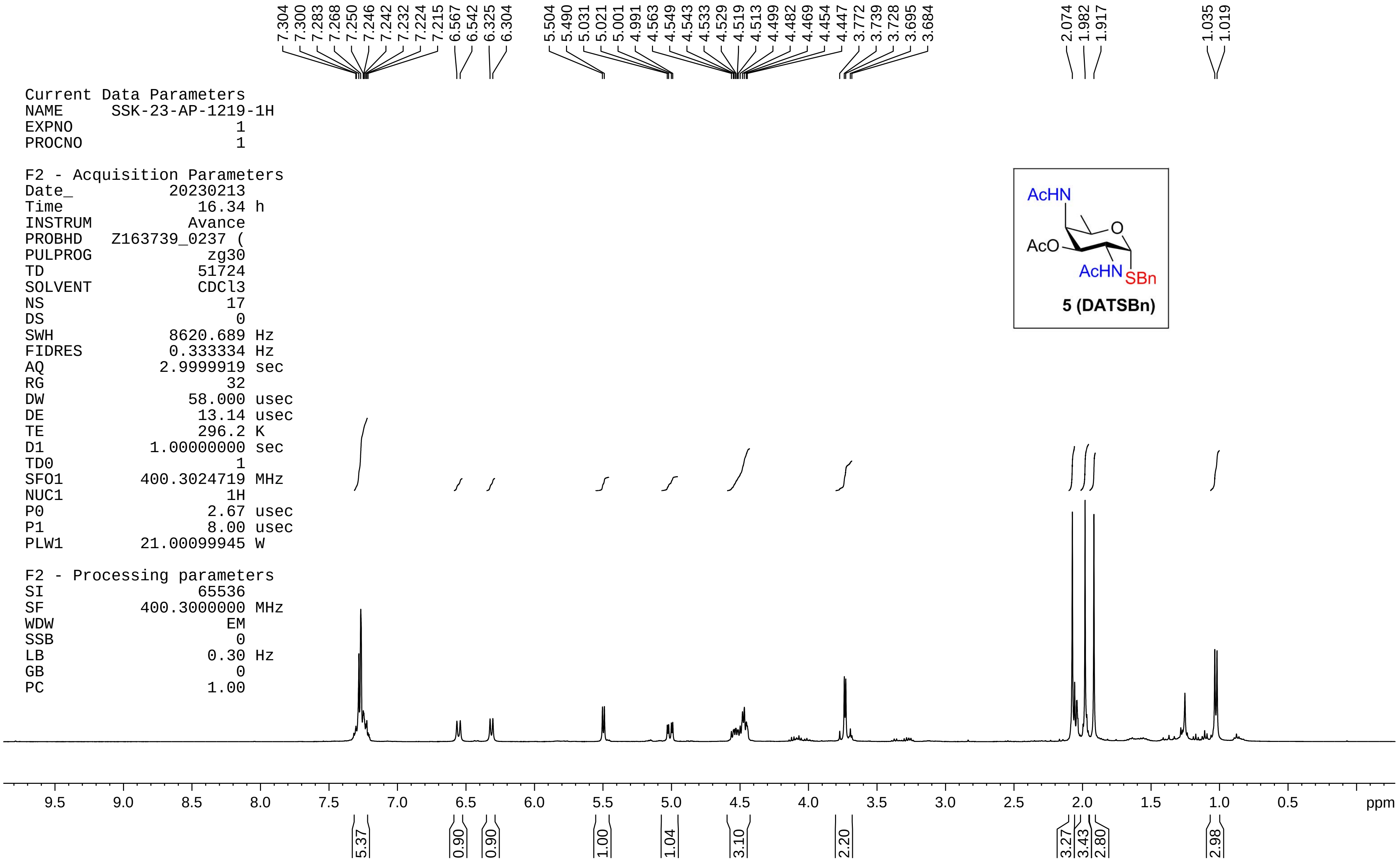

Current Data Parameters
NAME SSK-23-AP-1219-1H
EXPNO 1
PROCNO 1

F2 - Acquisition Parameters
Date_ 20230213
Time 16.34 h
INSTRUM Avance
PROBHD Z163739_0237 (
PULPROG zg30
TD 51724
SOLVENT CDCl3
NS 17
DS 0
SWH 8620.689 Hz
FIDRES 0.333334 Hz
AQ 2.9999919 sec
RG 32
DW 58.000 usec
DE 13.14 usec
TE 296.2 K
D1 1.00000000 sec
TD0 1
SFO1 400.3024719 MHz
NUC1 1H
P0 2.67 usec
P1 8.00 usec
PLW1 21.00099945 W

F2 - Processing parameters
SI 65536
SF 400.3000000 MHz
WDW EM
SSB 0
LB 0.30 Hz
GB 0
PC 1.00

5 (DATSBn)

SSK-23-AP-1219-13C

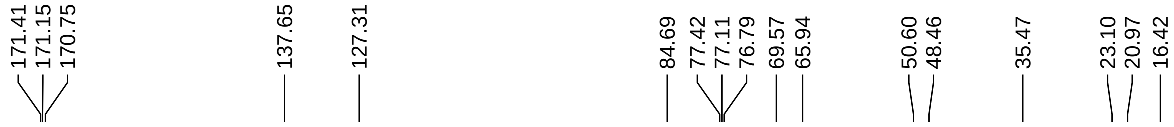


```
Current Data Parameters
NAME      SSK-23-AP-1219-13C
EXPNO                      3
PROCNO                     1

F2 - Acquisition Parameters
Date_             20230213
Time                 16.36 h
INSTRUM             Avance
PROBHD    Z163739_0237 (
PULPROG             zgpg30
TD                   65536
SOLVENT              CDCl3
NS                      21
DS                       0
SWH              27777.777 Hz
FIDRES            0.847710 Hz
AQ               1.1796480 sec
RG                     101
DW                  18.000 usec
DE                    6.50 usec
TE                   296.3 K
D1              1.00000000 sec
D11             0.03000000 sec
TD0                      1
SFO1           100.6669898 MHz
NUC1                   13C
P0                    2.67 usec
P1                    8.00 usec
PLW1           97.90799713 W
SFO2           400.3016012 MHz
NUC2                    1H
CPDPRG[2           waltz65
PCPD2                90.00 usec
PLW2           21.00099945 W
PLW12           0.16593000 W
PLW13           0.08346300 W

F2 - Processing parameters
SI                   32768
SF             100.6555151 MHz
WDW                     EM
SSB                      0
LB                    1.00 Hz
GB                       0
PC                    1.40
```

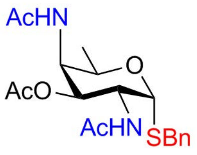

5 (DATSBn)

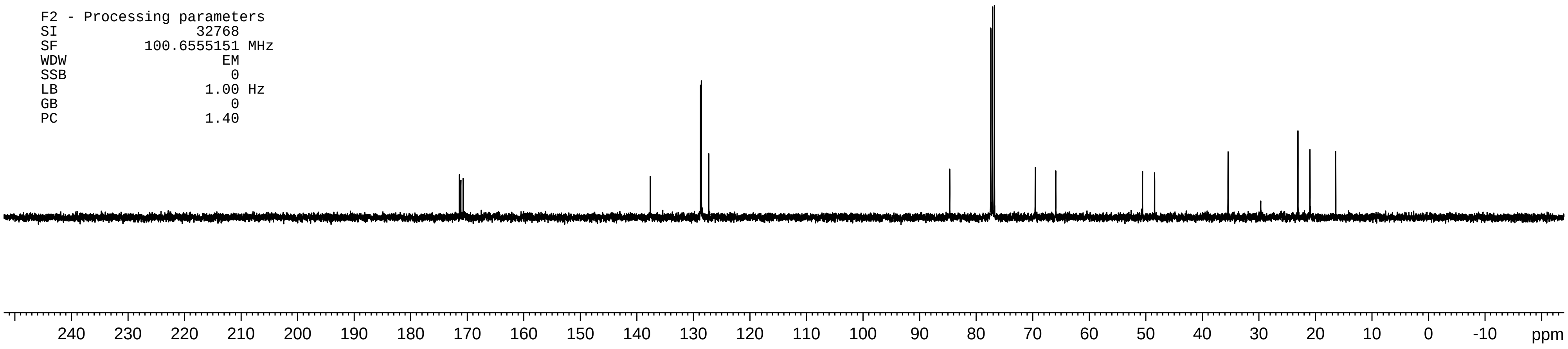

SSK-23-AP-1219-DEPT

Current Data Parameters
NAME SSK-23-AP-1219-DEPT
EXPNO 5
PROCNO 1

F2 - Acquisition Parameters
Date_ 20230213
Time 16.37 h
INSTRUM Avance
PROBHD Z163739_0237 (
PULPROG deptsp135
TD 65536
SOLVENT CDCl3
NS 11
DS 0
SWH 27777.777 Hz
FIDRES 0.847710 Hz
AQ 1.1796480 sec
RG 101
DW 18.000 usec
DE 6.50 usec
TE 296.3 K
CNST2 145.0000000
D1 1.00000000 sec
D2 0.00344828 sec
D12 0.00002000 sec
TD0 1
SFO1 100.6669898 MHz
NUC1 13C
P1 8.00 usec
P13 2000.00 usec
PLW0 0 W
PLW1 97.90799713 W
SPNAM[5] Crp60comp.4
SPOAL5 0.500
SPOFFS5 0 Hz
SPW5 9.57390022 W
SFO2 400.3016012 MHz
NUC2 1H
CPDPRG[2 waltz65
P3 8.00 usec
P4 16.00 usec
PCPD2 90.00 usec
PLW2 21.00099945 W
PLW12 0.16593000 W

F2 - Processing parameters
SI 32768
SF 100.6555151 MHz
WDW EM
SSB 0
LB 1.00 Hz
GB 0
PC 1.40

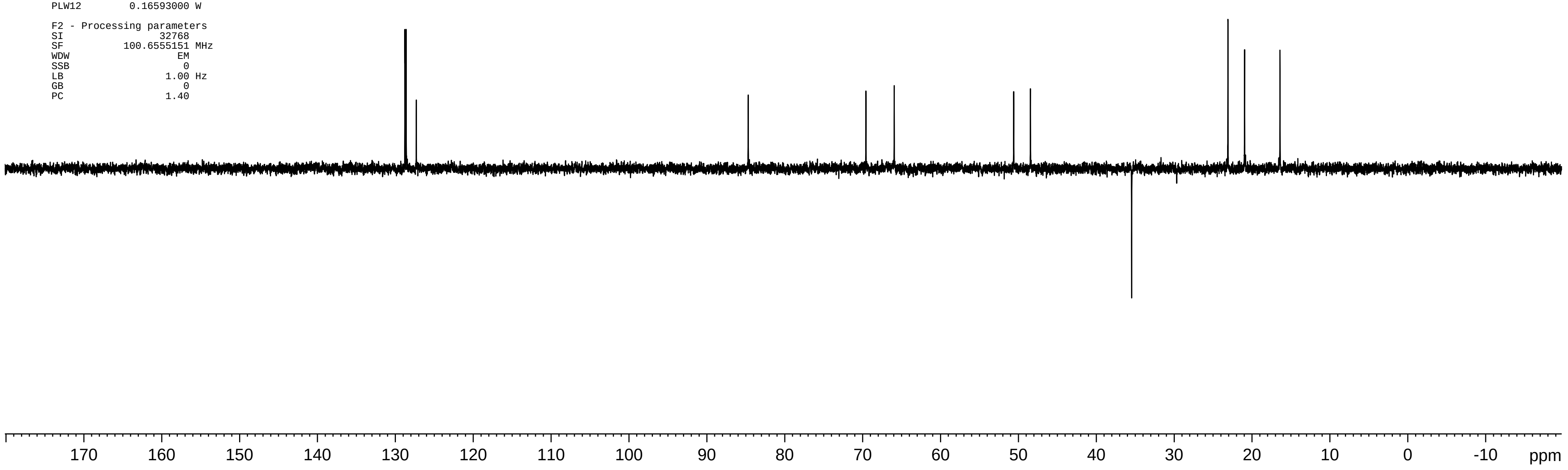

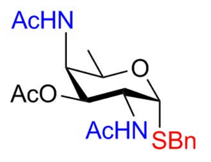

5 (DATSBn)

SSK-23-AP-1219-COSY

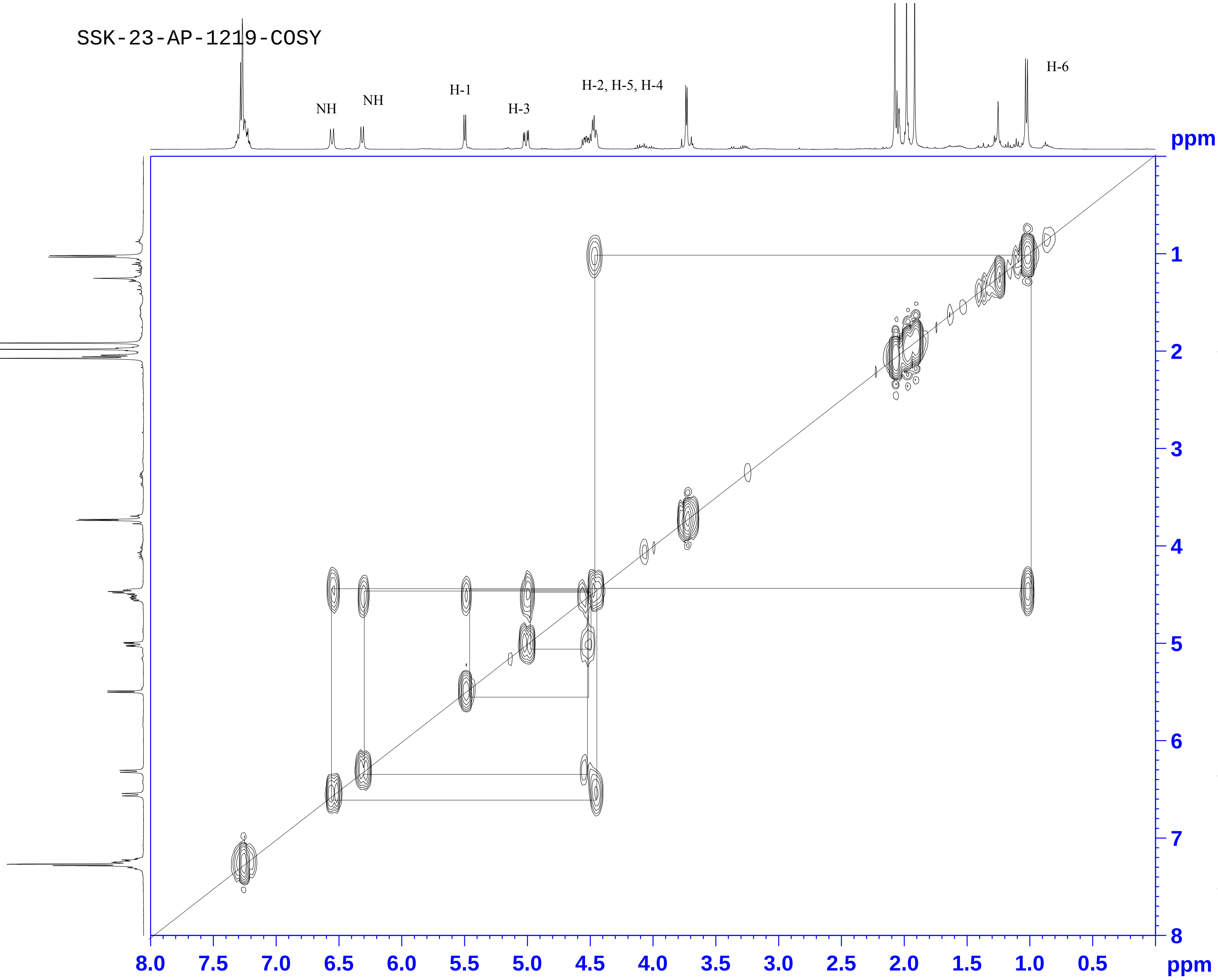

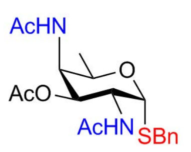

5 (DATSBn)

Current Data Parameters
NAME SSK-23-AP-1219-COSY
EXPNO 7
PROCNO 1

F2 - Acquisition Parameters
Date_ 20230213
Time 16.46 h
INSTRUM Avance
PROBHD Z163739_0237 (
PULPROG cosygpppqf
TD 2048
SOLVENT CDCl3
NS 4
DS 0
SWH 8620.689 Hz
FIDRES 8.418642 Hz
AQ 0.1187840 sec
RG 64
DW 58.000 usec
DE 6.50 usec
TE 296.2 K
D0 0.00000300 sec
D1 1.00000000 sec
D11 0.03000000 sec
D12 0.00002000 sec
D13 0.00000400 sec
D16 0.00020000 sec
IN0 0.00011355 sec
TDav 1
SFO1 400.3024018 MHz
NUC1 1H
P0 8.00 usec
P1 8.00 usec
P17 2500.00 usec
PLW1 21.00099945 W
PLW10 1.49339998 W
GPNAM[1] SMSQ10.100
GPZ1 10.00 %
P16 1000.00 usec

======== F1 INDIRECT DIMENSION ========
td1 128
sw_F1 22.000000

F1 - Acquisition parameters
TD 102
SFO1 400.3024 MHz
FIDRES 172.680252 Hz
SW 22.000 ppm
FnMODE QF

F2 - Processing parameters
SI 1024
SF 400.3000000 MHz
WDW QSINE
SSB 0
LB 0 Hz
GB 0
PC 1.40

F1 - Processing parameters
SI 1024
MC2 QF
SF 400.3000000 MHz
WDW QSINE
SSB 0
LB 0 Hz
GB 0

ssk-23-ap-fuc-n3-sp-1h

Current Data Parameters
NAME   ssk-23-ap-fuc-n3-sp-1h
EXPNO   17
PROCNO   1

F2 - Acquisition Parameters
Date_   20230417
Time   11.39 h
INSTRUM spect
PROBHD   Z119470_0087 (
PULPROG zg30
TD   65536
SOLVENT CDCl3
NS   7
DS   0
SWH   10000.000 Hz
FIDRES   0.305176 Hz
AQ   3.2767999 sec
RG   30.72
DW   50.000 usec
DE   6.50 usec
TE   298.5 K
D1   1.00000000 sec
TD0   1
SFO1   500.1330885 MHz
NUC1   1H
P0   4.45 usec
P1   13.35 usec
PLW1   16.00000000 W

F2 - Processing parameters
SI   65536
SF   500.1300000 MHz
WDW   EM
SSB   0
LB   0.30 Hz
GB   0
PC   1.00

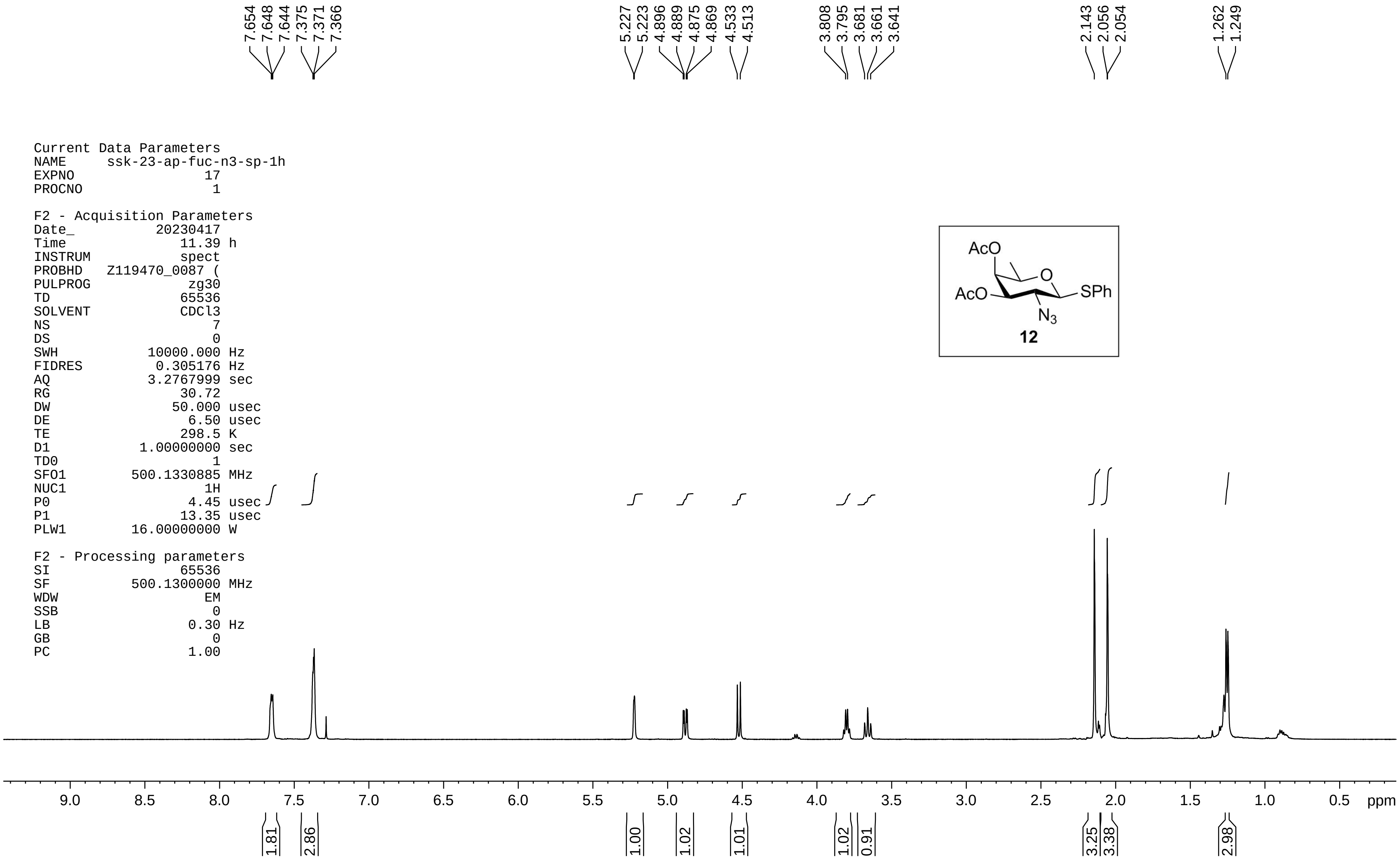

ssk-23-ap-fuc-n3-sp-13c

Current Data Parameters
NAME ssk-23-ap-fuc-n3-sp-13c
EXPNO 18
PROCNO 1

F2 - Acquisition Parameters
Date_ 20230417
Time 11.39 h
INSTRUM spect
PROBHD Z119470_0087 (
PULPROG zgpg30
TD 65536
SOLVENT CDCl3
NS 22
DS 0
SWH 29761.904 Hz
FIDRES 0.908261 Hz
AQ 1.1010048 sec
RG 197.27
DW 16.800 usec
DE 6.50 usec
TE 298.3 K
D1 1.00000000 sec
D11 0.03000000 sec
TD0 1
SFO1 125.7703637 MHz
NUC1 13C
P0 2.97 usec
P1 8.90 usec
PLW1 103.00000000 W
SFO2 500.1320005 MHz
NUC2 1H
CPDPRG[2 waltz16
PCPD2 80.00 usec
PLW2 16.00000000 W
PLW12 0.44556001 W
PLW13 0.22411001 W

F2 - Processing parameters
SI 32768
SF 125.7577890 MHz
WDW EM
SSB 0
LB 1.00 Hz
GB 0
PC 1.40

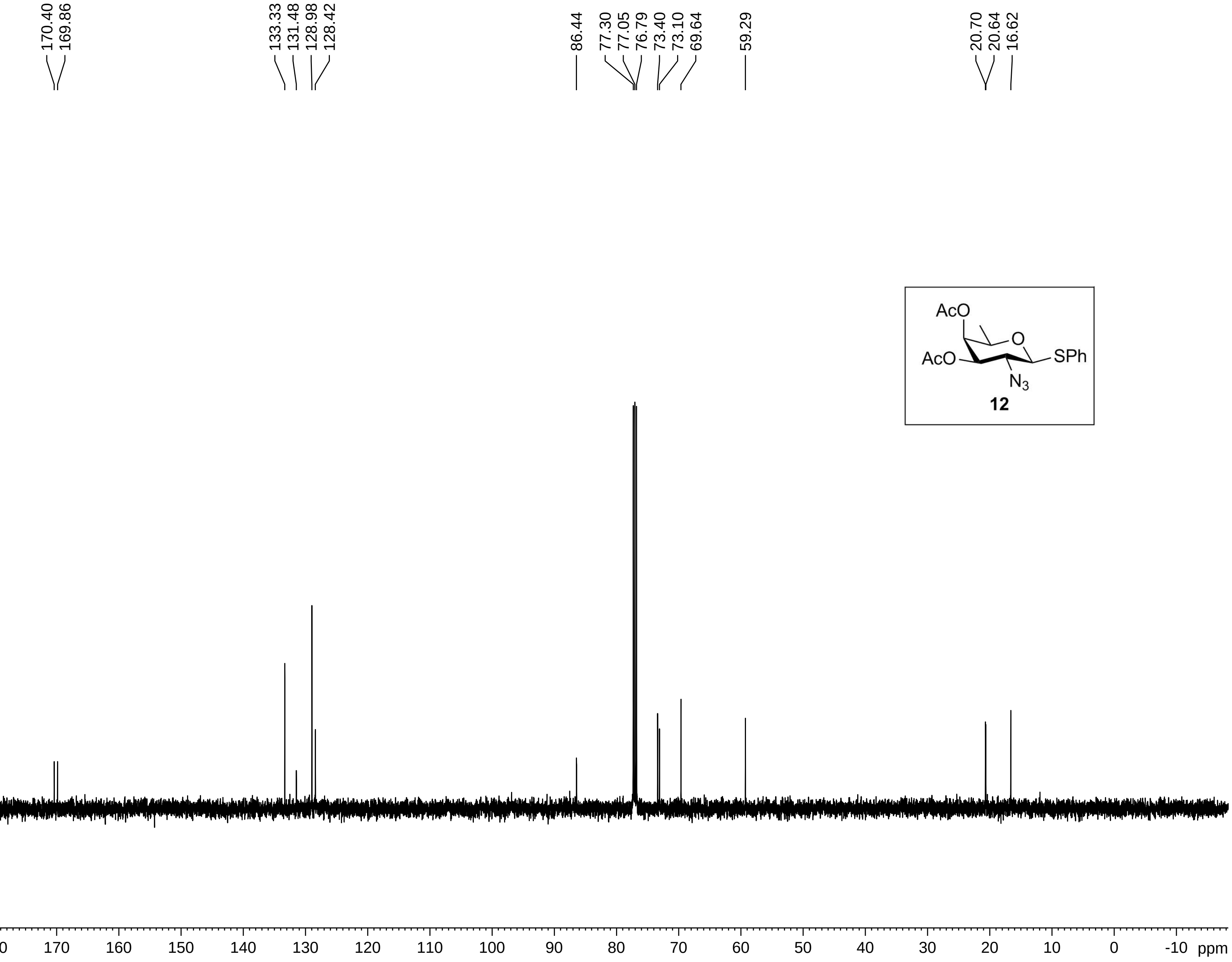

12

SSK-23-AP-1048-1H

Current Data Parameters
NAME SSK-23-AP-1048-1H
EXPNO 10
PROCNO 1

F2 - Acquisition Parameters
Date_ 20220416
Time 8.36 h
INSTRUM Avance Neo 400
PROBHD Z163739_0226 (
PULPROG zg30
TD 51724
SOLVENT CDCl3
NS 18
DS 0
SWH 8620.689 Hz
FIDRES 0.333334 Hz
AQ 2.9999919 sec
RG 32
DW 58.000 usec
DE 13.14 usec
TE 301.1 K
D1 1.00000000 sec
TD0 1
SFO1 400.1324708 MHz
NUC1 1H
P0 2.67 usec
P1 8.00 usec
PLW1 25.07999992 W

F2 - Processing parameters
SI 65536
SF 400.1300000 MHz
WDW EM
SSB 0
LB 0.30 Hz
GB 0
PC 1.00

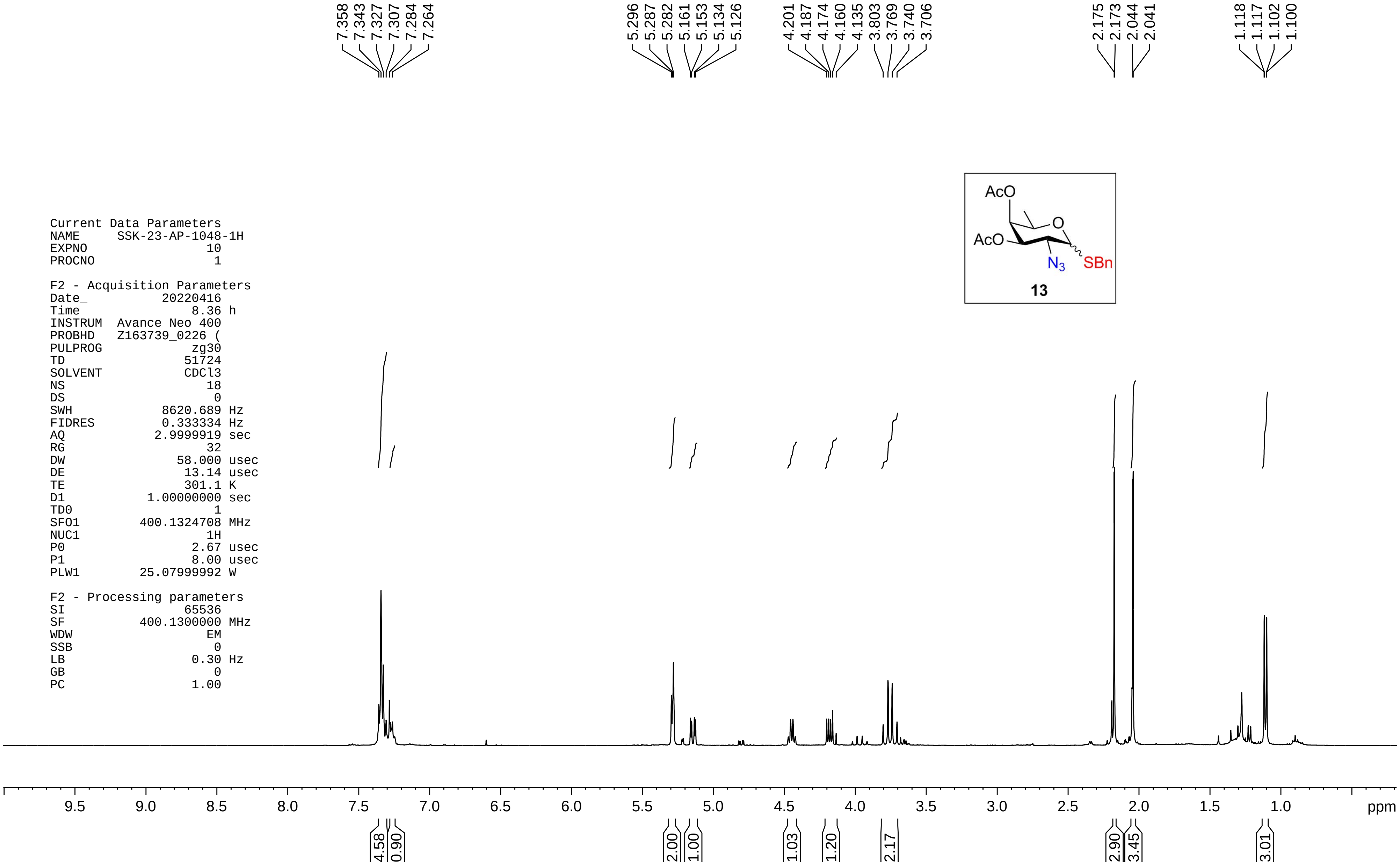

SSK-23-AP-1048-13C

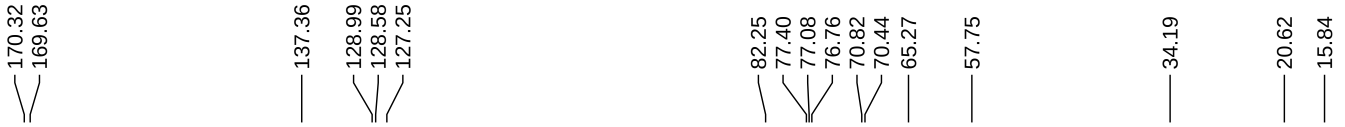


```
Current Data Parameters
NAME     SSK-23-AP-1048-13C
EXPNO                    12
PROCNO                    1

F2 - Acquisition Parameters
Date_          20220416
Time               8.39 h
INSTRUM  Avance Neo 400
PROBHD   Z163739_0226 (
PULPROG          zgpg30
TD                65536
SOLVENT           CDCl3
NS                   44
DS                    2
SWH           27777.777 Hz
FIDRES         0.847710 Hz
AQ            1.1796480 sec
RG                  101
DW               18.000 usec
DE                 6.50 usec
TE                301.2 K
D1           1.00000000 sec
D11          0.03000000 sec
TD0                   1
SFO1        100.6242384 MHz
NUC1                13C
P0                 2.67 usec
P1                 8.00 usec
PLW1        99.33999634 W
SFO2        400.1316005 MHz
NUC2                 1H
CPDPRG[2        waltz65
PCPD2             90.00 usec
PLW2        25.07999992 W
PLW12        0.19815999 W
PLW13        0.09967500 W

F2 - Processing parameters
SI                32768
SF          100.6127685 MHz
WDW                  EM
SSB                   0
LB                 1.00 Hz
GB                    0
PC                 1.40
```

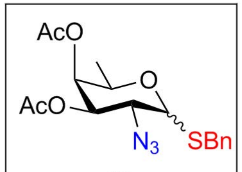

13

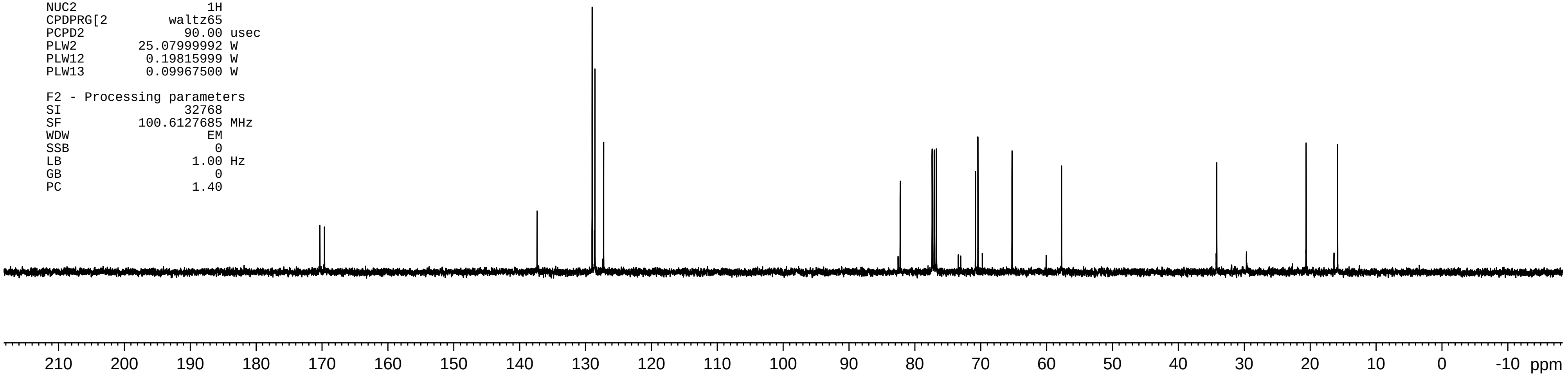

SSK-23-AP-1048-DEPT

```
Current Data Parameters
NAME     SSK-23-AP-1048-DEPT
EXPNO                    14
PROCNO                    1

F2 - Acquisition Parameters
Date_          20220416
Time               8.40 h
INSTRUM  Avance Neo 400
PROBHD   Z163739_0226 (
PULPROG       deptsp135
TD                65536
SOLVENT           CDCl3
NS                   18
DS                    0
SWH           27777.777 Hz
FIDRES         0.847710 Hz
AQ            1.1796480 sec
RG                  101
DW               18.000 usec
DE                 6.50 usec
TE                301.4 K
CNST2       145.0000000
D1           1.00000000 sec
D2           0.00344828 sec
D12          0.00002000 sec
TD0                   1
SFO1        100.6242384 MHz
NUC1                13C
P1                 8.00 usec
P13             2000.00 usec
PLW0                  0 W
PLW1        99.33999634 W
SPNAM[5]    Crp60comp.4
SPOAL5            0.500
SPOFFS5               0 Hz
SPW5         9.71399975 W
SFO2        400.1316005 MHz
NUC2                 1H
CPDPRG[2        waltz65
P3                 8.00 usec
P4                16.00 usec
PCPD2             90.00 usec
PLW2        25.07999992 W
PLW12        0.19815999 W

F2 - Processing parameters
SI                32768
SF          100.6127685 MHz
WDW                  EM
SSB                   0
LB                 1.00 Hz
GB                    0
PC                 1.40
```

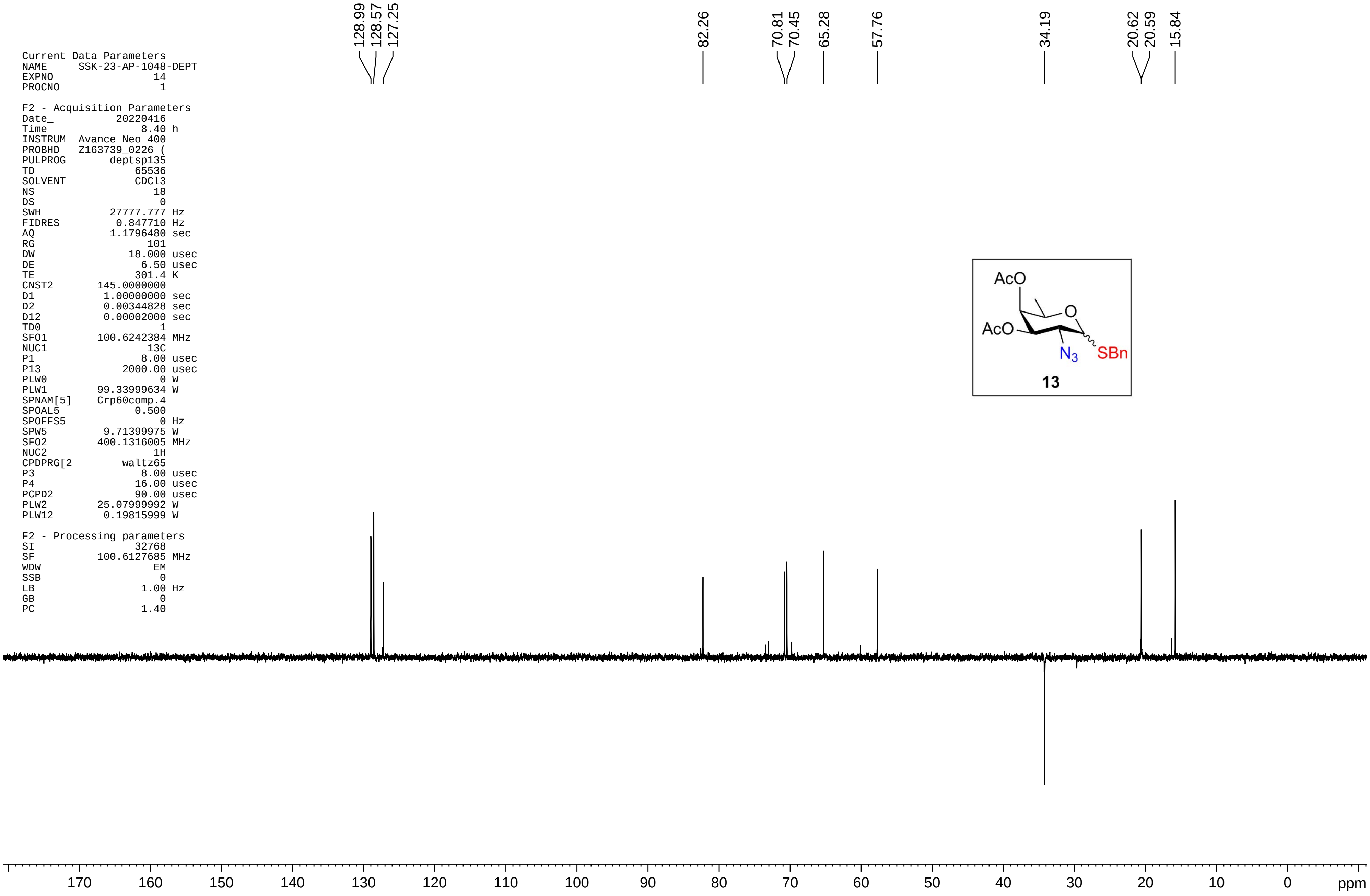

13

SSK-23-AP-1048-COSY

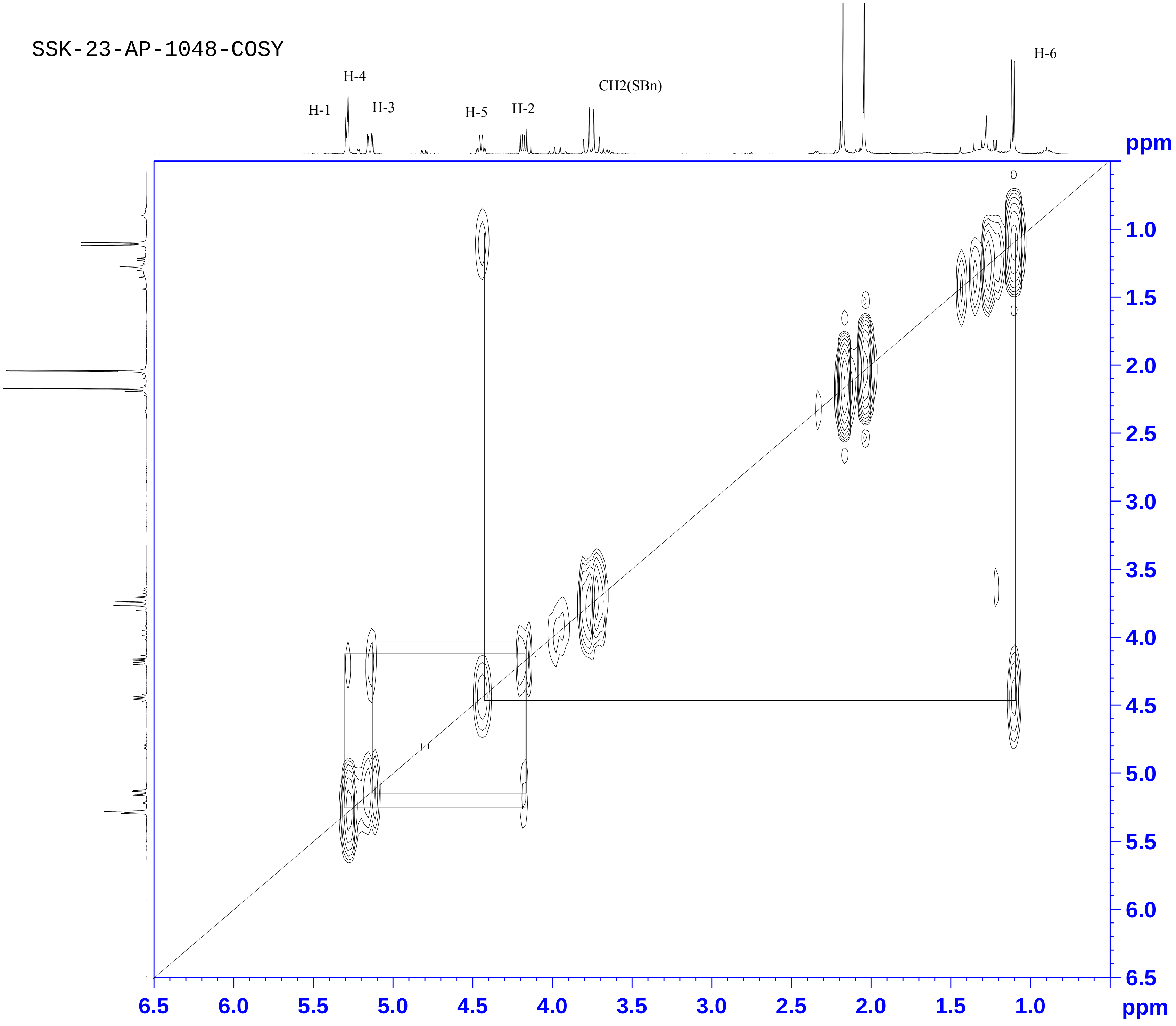

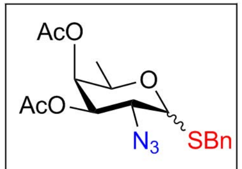

13

```
Current Data Parameters
NAME        SSK-23-AP-1048-COSY
EXPNO                    16
PROCNO                    1

F2 - Acquisition Parameters
Date_              20220416
Time                 8.45 h
INSTRUM    Avance Neo 400
PROBHD     Z163739_0226 (
PULPROG         cosygpppqf
TD                     2048
SOLVENT               CDCl3
NS                        4
DS                        0
SWH             8620.689 Hz
FIDRES          8.418642 Hz
AQ             0.1187840 sec
RG                       64
DW                 58.000 usec
DE                   6.50 usec
TE                  301.4 K
D0            0.00000300 sec
D1            1.00000000 sec
D11           0.03000000 sec
D12           0.00002000 sec
D13           0.00000400 sec
D16           0.00020000 sec
IN0           0.00011360 sec
TDav                      1
SFO1         400.1324708 MHz
NUC1                     1H
P0                   8.00 usec
P1                   8.00 usec
P17               2500.00 usec
PLW1          25.07999992 W
PLW10          1.78349996 W
GPNAM[1]        SMSQ10.100
GPZ1                10.00 %
P16               1000.00 usec

======== F1 INDIRECT DIMENSION ========
td1        128
sw_F1      21.999996

F1 - Acquisition parameters
TD                       55
SFO1           400.1325 MHz
FIDRES       320.102448 Hz
SW                   22.000 ppm
FnMODE                   QF

F2 - Processing parameters
SI                     1024
SF           400.1300000 MHz
WDW                   QSINE
SSB                       0
LB                        0 Hz
GB                        0
PC                     1.40

F1 - Processing parameters
SI                     1024
MC2                      QF
SF           400.1300000 MHz
WDW                   QSINE
SSB                       0
LB                        0 Hz
GB                        0
```

SSK-23-AP-1048-HSQC

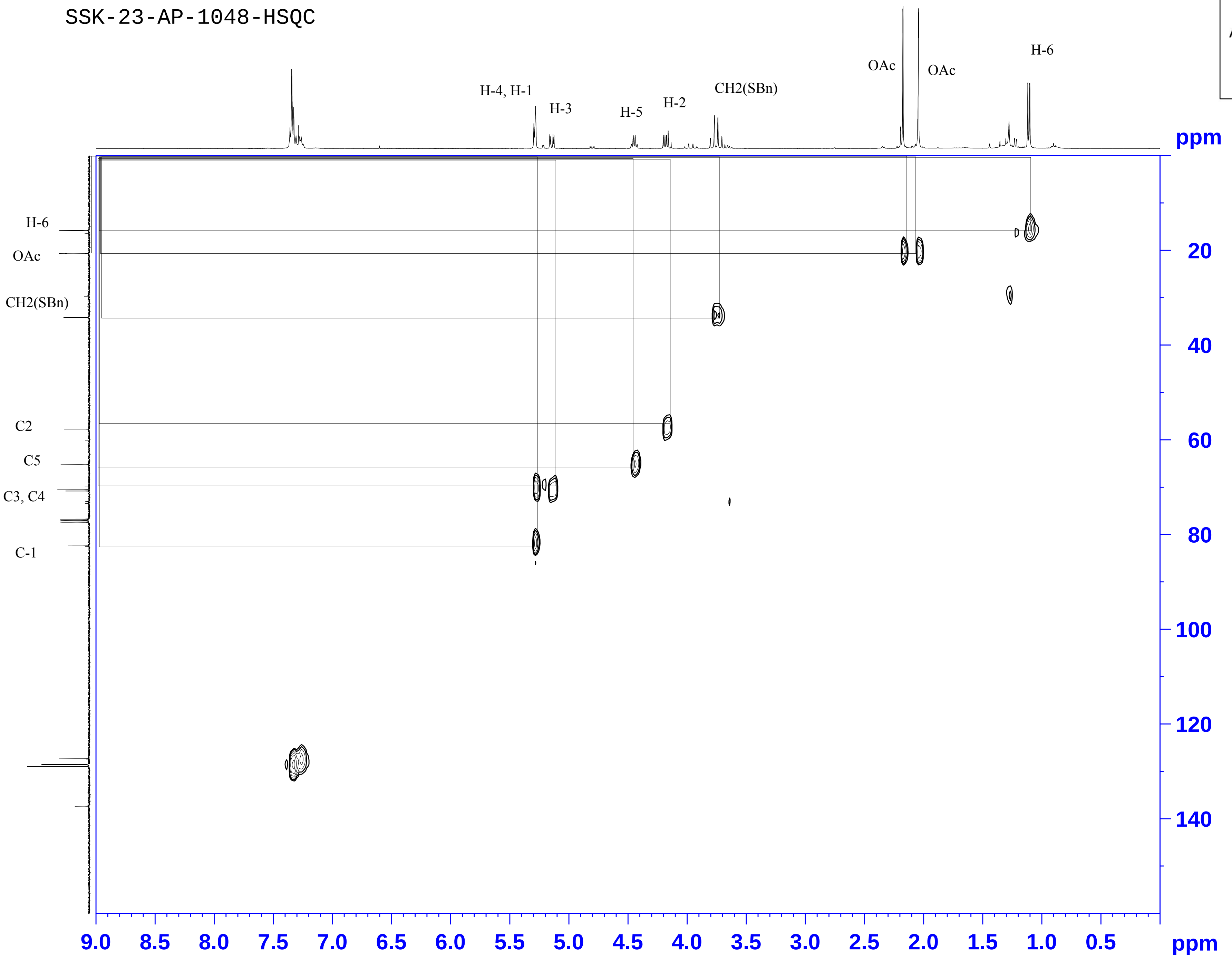

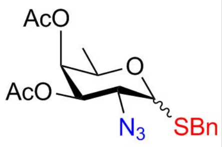

13

```
Current Data Parameters
NAME      SSK-23-AP-1048-HSQC
EXPNO                    18
PROCNO                    1

F2 - Acquisition Parameters
Date_              20220416
Time                8.53 h
INSTRUM   Avance Neo 400
PROBHD    Z163739_0226 (
PULPROG   hsqcedetgpsisp2.3
TD                     2048
SOLVENT               CDCl3
NS                        4
DS                        0
SWH              8620.689 Hz
FIDRES           8.418642 Hz
AQ              0.1187840 sec
RG                      101
DW                 58.000 usec
DE                   6.50 usec
TE                  301.1 K
CNST2         145.0000000
CNST17         -0.5000000
D0             0.00000300 sec
D1             1.00000000 sec
D4             0.00172414 sec
D11            0.03000000 sec
D16            0.00020000 sec
D21            0.00344800 sec
D24            0.00086200 sec
IN0            0.00001801 sec
TDav                      1
ZGOPTNS
SFO1          400.1324708 MHz
NUC1                     1H
P1                     8.00 usec
P2                    16.00 usec
PLW1          25.07999992 W
SFO2          100.6242384 MHz
NUC2                    13C
CPDPRG[2              garp4
P3                     8.00 usec
P14                  500.00 usec
P24                 2000.00 usec
P31                 2119.00 usec
PCPD2                 80.00 usec
PLW0                      0 W
PLW2          99.33999634 W
PLW12          0.99339998 W
SPNAM[3] Crp60,0.5,20.1
SPOAL3                0.500
SPOFFS3                   0 Hz
SPW3           9.71399975 W
SPNAM[7]     Crp60comp.4
SPOAL7                0.500
SPOFFS7                   0 Hz
SPW7           9.71399975 W
SPNAM[18   Crp60_xfilt.2
SPOAL18               0.500
SPOFFS18                  0 Hz
SPW18          1.87129998 W
GPNAM[1]      SMSQ10.100
GPZ1                  80.00 %
GPNAM[2]      SMSQ10.100
GPZ2                  20.10 %
GPNAM[3]      SMSQ10.100
GPZ3                  11.00 %
GPNAM[4]      SMSQ10.100
GPZ4                  -5.00 %
P16                 1000.00 usec
P19                  600.00 usec

======== F1 INDIRECT DIMENSION ========
td1      1024
sw_F1    275.989227

F1 - Acquisition parameters
TD                       82
SFO1               100.6242 MHz
FIDRES           677.271606 Hz
SW                  275.959 ppm
FnMODE      Echo-Antiecho

F2 - Processing parameters
SI                     1024
SF            400.1300000 MHz
WDW                   QSINE
SSB                       2
LB                        0 Hz
GB                        0
PC                     1.40

F1 - Processing parameters
SI                     1024
MC2          echo-antiecho
SF            100.6127685 MHz
WDW                   QSINE
SSB                       2
LB                        0 Hz
GB                        0
```

SSK-23-AP-1049-1H

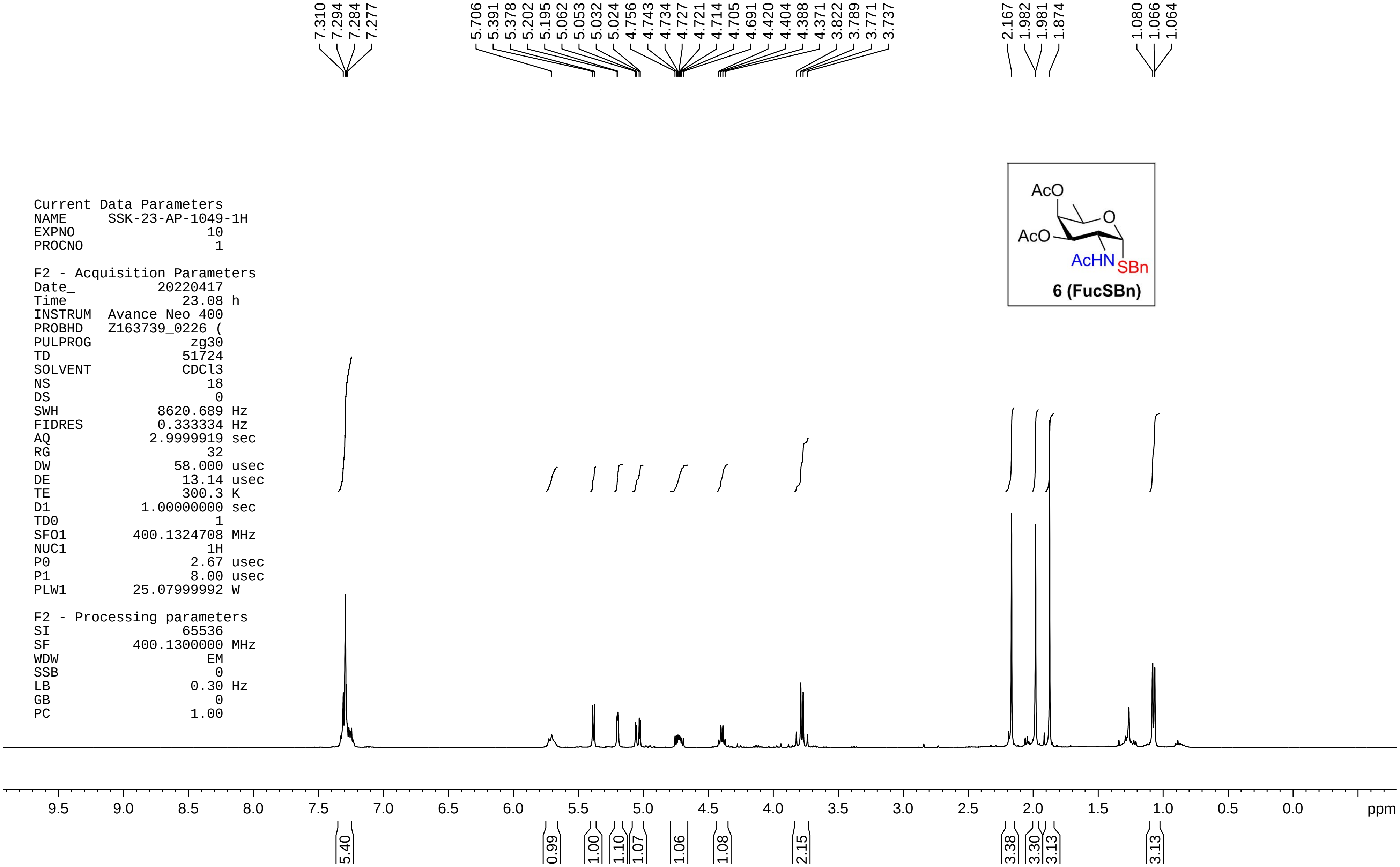


```
Current Data Parameters
NAME      SSK-23-AP-1049-1H
EXPNO                    10
PROCNO                    1

F2 - Acquisition Parameters
Date_            20220417
Time                23.08 h
INSTRUM   Avance Neo 400
PROBHD    Z163739_0226 (
PULPROG              zg30
TD                  51724
SOLVENT             CDCl3
NS                     18
DS                      0
SWH              8620.689 Hz
FIDRES           0.333334 Hz
AQ              2.9999919 sec
RG                     32
DW                 58.000 usec
DE                  13.14 usec
TE                  300.3 K
D1             1.00000000 sec
TD0                     1
SFO1          400.1324708 MHz
NUC1                   1H
P0                   2.67 usec
P1                   8.00 usec
PLW1          25.07999992 W

F2 - Processing parameters
SI                  65536
SF            400.1300000 MHz
WDW                    EM
SSB                     0
LB                   0.30 Hz
GB                      0
PC                   1.00
```

SSK-23-AP-1049-13C

```
Current Data Parameters
NAME      SSK-23-AP-1049-13C
EXPNO                   12
PROCNO                   1

F2 - Acquisition Parameters
Date_           20220417
Time               23.10 h
INSTRUM  Avance Neo 400
PROBHD   Z163739_0226 (
PULPROG          zgpg30
TD                65536
SOLVENT           CDCl3
NS                   20
DS                    2
SWH           27777.777 Hz
FIDRES         0.847710 Hz
AQ            1.1796480 sec
RG                  101
DW               18.000 usec
DE                 6.50 usec
TE                300.3 K
D1           1.00000000 sec
D11          0.03000000 sec
TD0                   1
SFO1        100.6242384 MHz
NUC1                13C
P0                 2.67 usec
P1                 8.00 usec
PLW1        99.33999634 W
SFO2        400.1316005 MHz
NUC2                 1H
CPDPRG[2        waltz65
PCPD2             90.00 usec
PLW2        25.07999992 W
PLW12        0.19815999 W
PLW13        0.09967500 W

F2 - Processing parameters
SI                32768
SF          100.6127685 MHz
WDW                  EM
SSB                   0
LB                 1.00 Hz
GB                    0
PC                 1.40
```

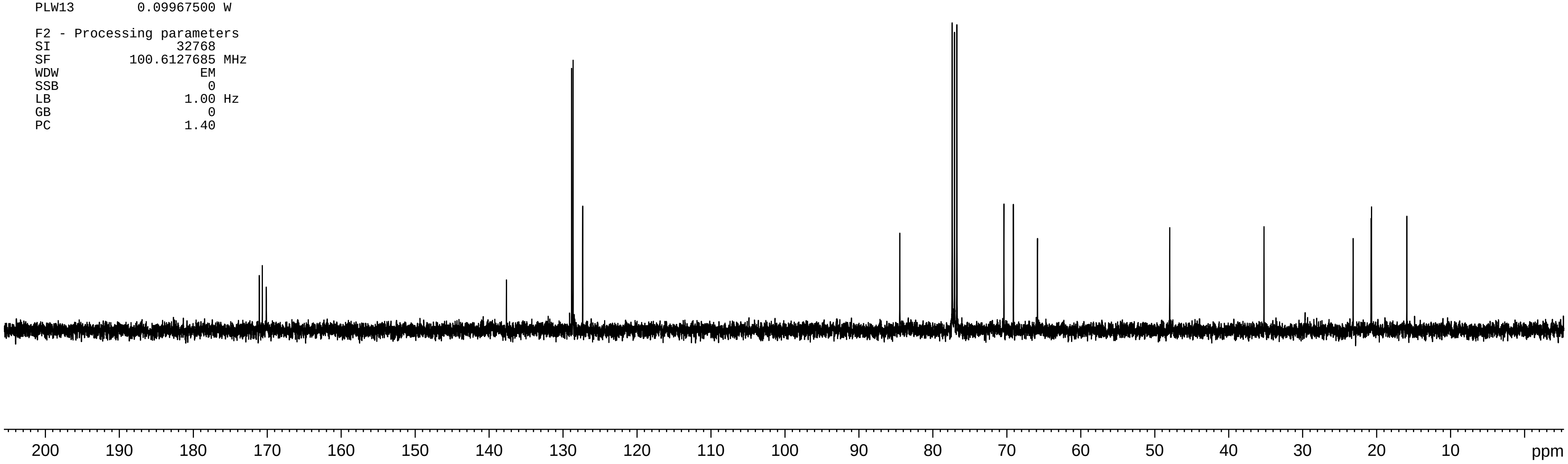

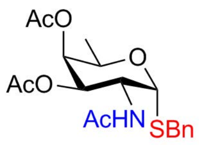

6 (FucSBn)

SSK-23-AP-1049-DEPT

```
Current Data Parameters
NAME     SSK-23-AP-1049-DEPT
EXPNO              16
PROCNO              1

F2 - Acquisition Parameters
Date_          20220417
Time              23.18 h
INSTRUM  Avance Neo 400
PROBHD   Z163739_0226 (
PULPROG       deptsp135
TD                65536
SOLVENT           CDCl3
NS                   11
DS                    4
SWH           27777.777 Hz
FIDRES         0.847710 Hz
AQ            1.1796480 sec
RG                  101
DW               18.000 usec
DE                 6.50 usec
TE                300.4 K
CNST2       145.0000000
D1           1.00000000 sec
D2           0.00344828 sec
D12          0.00002000 sec
TD0                   1
SFO1        100.6242384 MHz
NUC1                13C
P1                 8.00 usec
P13             2000.00 usec
PLW0                  0 W
PLW1        99.33999634 W
SPNAM[5]    Crp60comp.4
SPOAL5            0.500
SPOFFS5               0 Hz
SPW5         9.71399975 W
SFO2        400.1316005 MHz
NUC2                 1H
CPDPRG[2        waltz65
P3                 8.00 usec
P4                16.00 usec
PCPD2             90.00 usec
PLW2        25.07999992 W
PLW12        0.19815999 W

F2 - Processing parameters
SI                32768
SF          100.6127685 MHz
WDW                  EM
SSB                   0
LB                 1.00 Hz
GB                    0
PC                 1.40
```

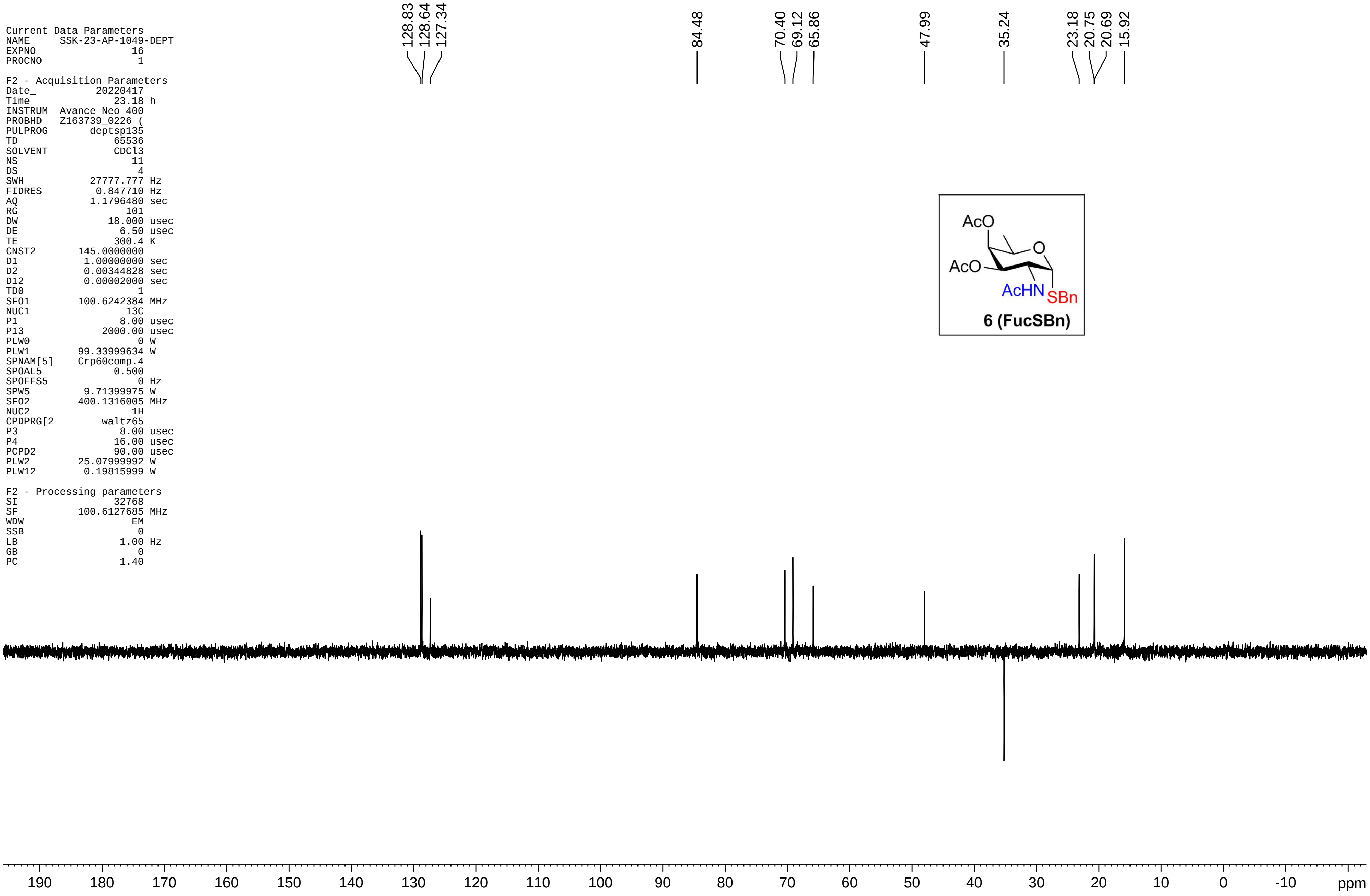

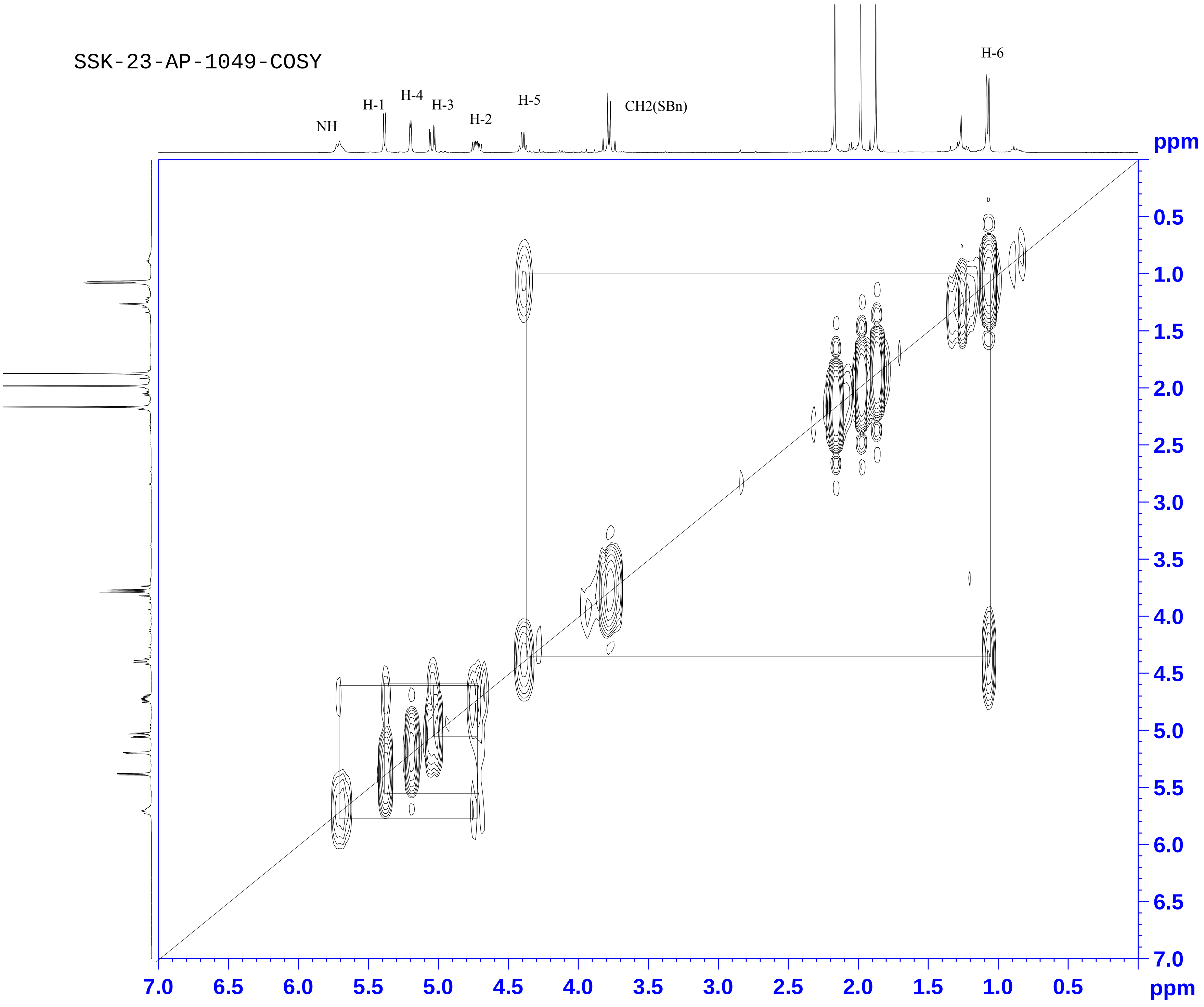

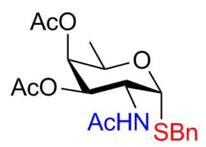

6 (FucSBn)

```
Current Data Parameters
NAME       SSK-23-AP-1049-COSY
EXPNO                    14
PROCNO                    1

F2 - Acquisition Parameters
Date_              20220417
Time                  23.15 h
INSTRUM      Avance Neo 400
PROBHD       Z163739_0226 (
PULPROG          cosygpppqf
TD                     2048
SOLVENT               CDCl3
NS                        4
DS                        0
SWH             8620.689 Hz
FIDRES          8.418642 Hz
AQ             0.1187840 sec
RG                       64
DW                58.000 usec
DE                 6.50 usec
TE                 300.6 K
D0            0.00000300 sec
D1            1.00000000 sec
D11           0.03000000 sec
D12           0.00002000 sec
D13           0.00000400 sec
D16           0.00020000 sec
IN0           0.00011360 sec
TDav                      1
SFO1         400.1324708 MHz
NUC1                     1H
P0                  8.00 usec
P1                  8.00 usec
P17              2500.00 usec
PLW1         25.07999992 W
PLW10         1.78349996 W
GPNAM[1]        SMSQ10.100
GPZ1               10.00 %
P16              1000.00 usec

======== F1 INDIRECT DIMENSION ========
td1        128
sw_F1      21.999996

F1 - Acquisition parameters
TD                       53
SFO1            400.1325 MHz
FIDRES        332.181763 Hz
SW                   22.000 ppm
FnMODE                   QF

F2 - Processing parameters
SI                     1024
SF           400.1300000 MHz
WDW                   QSINE
SSB                       0
LB                        0 Hz
GB                        0
PC                     1.40

F1 - Processing parameters
SI                     1024
MC2                      QF
SF           400.1300000 MHz
WDW                   QSINE
SSB                       0
LB                        0 Hz
GB                        0
```
